# Supplementary material for: Chainsaw: protein domain segmentation with fully convolutional neural networks
Source: Bioinformatics. 2024 May 8;40(5):btae296. doi: 10.1093/bioinformatics/btae296 (PMC11256964; doi:10.1093/bioinformatics/btae296)
Supplement: btae296_Supplementary_Data [file btae296_supplementary_data.pdf]

Supplementary material

A Assessment metrics

A.1 Intersection over union

Following the approach of Merizo (19), for a given protein chain we compute the average intersection over union (IoU) between paired sets of predicted and ground-truth residues assigned to each domain. As a first step, each ground-truth domain is paired with a predicted domain such that the sum of all intersections over unions is maximised while respecting the following constraints: Each ground-truth domain (represented as a set of residue indices) T_i can have, at most, one paired predicted domain P_i. Second, each predicted domain can be assigned at most once. No IoU is computed for the sets of residues that are labelled as, or predicted to be non-domain residues. To generate a final score for the whole chain each domain-level IoU is weighted by the number of residues in the ground-truth domain:

$$\mathrm{IoU}_{\mathrm{chain}} = \sum_{i=1}^{n_{\mathrm{dom}}} \frac{|T_i \cap P_i|}{|T_i \cup P_i|} \cdot \frac{|T_i|}{\sum_{j=1}^{n_{\mathrm{dom}}} |T_j|}\,. \tag{7}$$

We additionally calculate the proportion of correctly parsed domains as the proportion of ground-truth domains where the domain-level IoU is 0.8 or greater.

A.2 Domain boundary distance score

The domain boundary distance score was introduced to assess domain boundary predictions in CASP 7. A detailed description of how the score is calculated is provided in (21). At a high level: each boundary is scored independently. Predicting within 1 residue of the true boundary scores 8 points, within 2 residues scores 7 and so on until the distance is 9 residues or more, at which point the score is 0. Each boundary score is divided by 8 so that scores per boundary are between 0 and 1. The final boundary distance score for the entire chain is then calculated as the sum of individual predicted boundary scores divided by the total number of domain boundaries. In order to ensure that over-prediction is penalized, the number of domain boundaries comes from the maximum number of domains in the target or the number of domains in the prediction (21).

B Proof that pairwise 2D distances specifies 3D points up to isometry

Theorem 1. *Let $\mathbf{x}_0, \ldots, \mathbf{x}_n$ and $\mathbf{y}_0, \ldots, \mathbf{y}_n$ be tuples representing points in the Euclidean space $\mathbb{E}^3$. Assume that for every pair of indices $i, j \in \{0, \ldots, n\}$, we have*

$$D(\mathbf{x}_i, \mathbf{x}_j) = D(\mathbf{y}_i, \mathbf{y}_j), \tag{8}$$

where D denotes the distance function on $\mathbb{E}^3$:

$$D(\mathbf{x}_i, \mathbf{x}_j) = \sqrt{\langle \mathbf{x}_i - \mathbf{x}_j, \mathbf{x}_i - \mathbf{x}_j \rangle}. \tag{9}$$

and $\langle \cdot, \cdot \rangle$ denotes the inner-product of two vectors. Then, there exists a unique bijective isometry $g : \mathbb{E}^3 \to \mathbb{E}^3$ such that $g(\mathbf{x}_i) = \mathbf{y}_i$ for each i.

Proof. **Step 1: Translation to Origin.** First, choose $\mathbf{x}_0$ and $\mathbf{y}_0$ and translate all points $\mathbf{x}_0, \ldots, \mathbf{x}_n$ and $\mathbf{y}_0, \ldots, \mathbf{y}_n$ in $\mathbb{E}^3$ such that $\mathbf{x}_0 = \mathbf{y}_0 = \mathbf{0}$. These translations preserve distances, so

$$D(\mathbf{x}_i, \mathbf{x}_0) = D(\mathbf{y}_i, \mathbf{y}_0), \tag{10}$$

and therefore:

$$||\mathbf{x}_i|| = ||\mathbf{y}_i|| \tag{11}$$

where $|| \cdot ||$ denotes the L2-norm of a vector.

Step 2: Equality of Distance Implies Equality of Inner Product.
From the definition of the distance function, we have

$$D(\mathbf{x}_i, \mathbf{x}_j)^2 = ||\mathbf{x}_i||^2 - 2\langle \mathbf{x}_i, \mathbf{x}_j \rangle + ||\mathbf{x}_j||^2. \tag{12}$$

Using Equations (8) and (11), we deduce

$$||\mathbf{x}_i||^2 - 2\langle \mathbf{x}_i, \mathbf{x}_j \rangle + ||\mathbf{x}_j||^2 = ||\mathbf{y}_i||^2 - 2\langle \mathbf{y}_i, \mathbf{y}_j \rangle + ||\mathbf{y}_j||^2$$

$$2\langle \mathbf{y}_i, \mathbf{y}_j \rangle - 2\langle \mathbf{x}_i, \mathbf{x}_j \rangle = ||\mathbf{y}_i||^2 - ||\mathbf{x}_i||^2 + ||\mathbf{y}_j||^2 - ||\mathbf{x}_j||^2 = 0$$

$$\langle \mathbf{y}_i, \mathbf{y}_j \rangle = \langle \mathbf{x}_i, \mathbf{x}_j \rangle. \tag{13}$$

Step 3: Identifying Basis Vectors.
Without loss of generality, choose vectors $\mathbf{x}_1$, $\mathbf{x}_2$, and $\mathbf{x}_3$ that form a basis for $\mathbb{E}^3$.

Lemma 1. *The Gram matrix of a set of vectors is non-singular if and only if the set of vectors are linearly independent.*

Let $\mathbf{G}$ be the 3×3 Gram matrix of $\mathbf{x}_1$, $\mathbf{x}_2$, $\mathbf{x}_3$, defined by

$$G_{i,j} = \langle \mathbf{x}_i, \mathbf{x}_j \rangle, \quad i, j = 1, 2, 3. \tag{14}$$

Since $\mathbf{y}_1$, $\mathbf{y}_2$, $\mathbf{y}_3$ share the same Gram matrix $\mathbf{G}$ (due to eq. 13), they also form a basis for $\mathbb{E}^3$.

Lemma 2. *If two sets of basis vectors have the same Gram matrix, there exists an isometry that maps one set to the other.*

Let $\mathbf{T}$ be a (3×3) matrix which applies the orthogonal transformation g, mapping $\mathbf{x}_1$, $\mathbf{x}_2$, $\mathbf{x}_3$ to $\mathbf{y}_1$, $\mathbf{y}_2$, $\mathbf{y}_3$.

Step 4: Extension to All Vectors.
Any vector $\mathbf{x}$ in $\mathbb{E}^3$ can be expressed as a linear combination of our basis vectors:

$$\mathbf{x}_i = c_{(i)1}\mathbf{x}_1 + c_{(i)2}\mathbf{x}_2 + c_{(i)3}\mathbf{x}_3, \tag{15}$$

for some $c_{(i)1}, c_{(i)2}, c_{(i)3} \in \mathbb{R}$.
A unique solution for the values $c_{(i)1}, c_{(i)2}, c_{(i)3}$ can be obtained using only the inner products of $\mathbf{x}_1, \mathbf{x}_2, \mathbf{x}_3$ with $\mathbf{x}_i$ We can write this as a system of linear equations in matrix form $\mathbf{G}\mathbf{c}_{(i)} = \mathbf{b}$, where

$$\mathbf{G} = \begin{pmatrix} \langle \mathbf{x}_1, \mathbf{x}_1 \rangle & \langle \mathbf{x}_1, \mathbf{x}_2 \rangle & \langle \mathbf{x}_1, \mathbf{x}_3 \rangle \\ \langle \mathbf{x}_2, \mathbf{x}_1 \rangle & \langle \mathbf{x}_2, \mathbf{x}_2 \rangle & \langle \mathbf{x}_2, \mathbf{x}_3 \rangle \\ \langle \mathbf{x}_3, \mathbf{x}_1 \rangle & \langle \mathbf{x}_3, \mathbf{x}_2 \rangle & \langle \mathbf{x}_3, \mathbf{x}_3 \rangle \end{pmatrix}$$

$$\mathbf{c}_{(i)} = \begin{pmatrix} c_{(i)1} \\ c_{(i)2} \\ c_{(i)3} \end{pmatrix}$$

$$\mathbf{b} = \begin{pmatrix} \langle \mathbf{x}_i, \mathbf{x}_1 \rangle \\ \langle \mathbf{x}_i, \mathbf{x}_2 \rangle \\ \langle \mathbf{x}_i, \mathbf{x}_3 \rangle \end{pmatrix}$$

Since $\mathbf{x}_1, \mathbf{x}_2, \mathbf{x}_3$ form a basis, the matrix $\mathbf{G}$ is invertible. Therefore, the system of equations has a unique solution for $\mathbf{c}_{(i)}$. Given that $\langle \mathbf{y}_i, \mathbf{y}_j \rangle = \langle \mathbf{x}_i, \mathbf{x}_j \rangle$ we see that matrix $\mathbf{G}$ and vector $\mathbf{b}$ will give rise to the same solutions for $c_{(i)1}, c_{(i)2}, c_{(i)3}$ satisfying the equation

$$\mathbf{y}_i = c_{(i)1}\mathbf{y}_1 + c_{(i)2}\mathbf{y}_2 + c_{(i)3}\mathbf{y}_3 \tag{16}$$

Finally, we note that the orthogonal transformation $\mathbf{T}$ maps every vector $\mathbf{x}_i$ to $\mathbf{y}_i$

$$\mathbf{T}\mathbf{x}_i = \mathbf{T}(c_{(i)1}\mathbf{x}_1 + c_{(i)2}\mathbf{x}_2 + c_{(i)3}\mathbf{x}_3) = c_{(i)1}\mathbf{y}_1 + c_{(i)2}\mathbf{y}_2 + c_{(i)3}\mathbf{y}_3 = \mathbf{y}_i \tag{17}$$

which completes the proof.

□

C NDO score

The NDO score is a score with a maximum of 1 which represents the proportion of residues that have been assigned to the correct domain. To understand the calculation, the score can be decomposed into scores for each predicted domain and scores for each true domain. Each predicted domain D_{p_i} gets an un-normalised score S_{p_i} which is the number of residues in the maximum intersection over all intersections with the ground-truth domains D_{t_j} minus the sum of the size of all its other intersections with ground-truth domains (all those that do not include the maximum intersection):

$$S_{p_i} = \max_j(|D_{p_i} \cap D_{t_j}|) - \sum_{k \neq j}(|D_{p_i} \cap D_{t_k}|)$$

Similarly, each ground-truth domain, D_{t_i}, gets an un-normalised score, S_{t_i}, which is the number of residues in the maximum intersection over all intersections with the predicted domains minus the sum of all its other intersections with predicted domains:

$$S_{t_i} = \max_j(|D_{t_i} \cap D_{p_j}|) - \sum_{k \neq j}(|D_{t_i} \cap D_{p_k}|)$$

The individual unnormalised predicted domain scores and ground truth domain scores are summed together, divided by two (to account for counting residues in both the true domain scores and predicted domain scores), before being divided by the maximum unnormalised score N_{res_t} which equals the number of residues assigned to domains in the ground-truth assignment:

$$S_{\text{NDO}} = \frac{1}{2 \cdot N_{\text{res}_t}} \sum_{i=1}^{D_{\text{pred}}} S_{p_i} + \sum_{j=1}^{D_{\text{true}}} S_{t_j}$$

D Additional results

D.1 Predicting CATH domain annotations mapped to AlphaFold models

When predicting domain boundaries on AlphaFold models as opposed to PDB structures we do not have a ground-truth set of labels. To overcome this problem we use SIFTS (31) to map PDB structures to their corresponding predicted structures in the AFDB. This enables us to map a subset of the CATH test set domain annotations onto AlphaFold models. Using this approach we compare Chainsaw's performance on 1039 samples from the CATH test set. Table 2 shows that we do not see any significant decrease in performance when predicting on AlphaFold models as opposed to PDB structures. However, it is important to note that this subset of AlphaFold models is not representative of the AFDB as a whole because it only contains sequences that have experimental PDB structures and have been annotated by CATH. In contrast with the AFDB overall we observe that this subset of AlphaFold models has better modelled structure and contains few proteins with long regions of non-secondary structure residues.

Table 2. Comparing Chainsaw performance on AlphaFold models versus experimental structures from the PDB

Dataset	Chainsaw NDO Score	
	on AlphaFold	on PDB
CATH n. dom. ≥ 1	0.93	0.94
CATH n. dom. ≥ 2	0.91	0.90
CATH n. dom ≥ 3	0.94	0.95

D.2 Performance on CASP 6 domain annotations

To evaluate Chainsaw on CASP 6, we trained a separate model from scratch to ensure no homologous domains were in the training data.

Table 3. Comparison of methods CASP 6 dataset

	Chainsaw	UniDoc	PUU	SWORD2	Merizo
Intersection over union	**0.89**	0.87	0.88	0.88	0.84
Proportion correct domains	**0.78**	0.74	0.72	0.77	0.69

D.3 Ablation analysis: secondary structure features

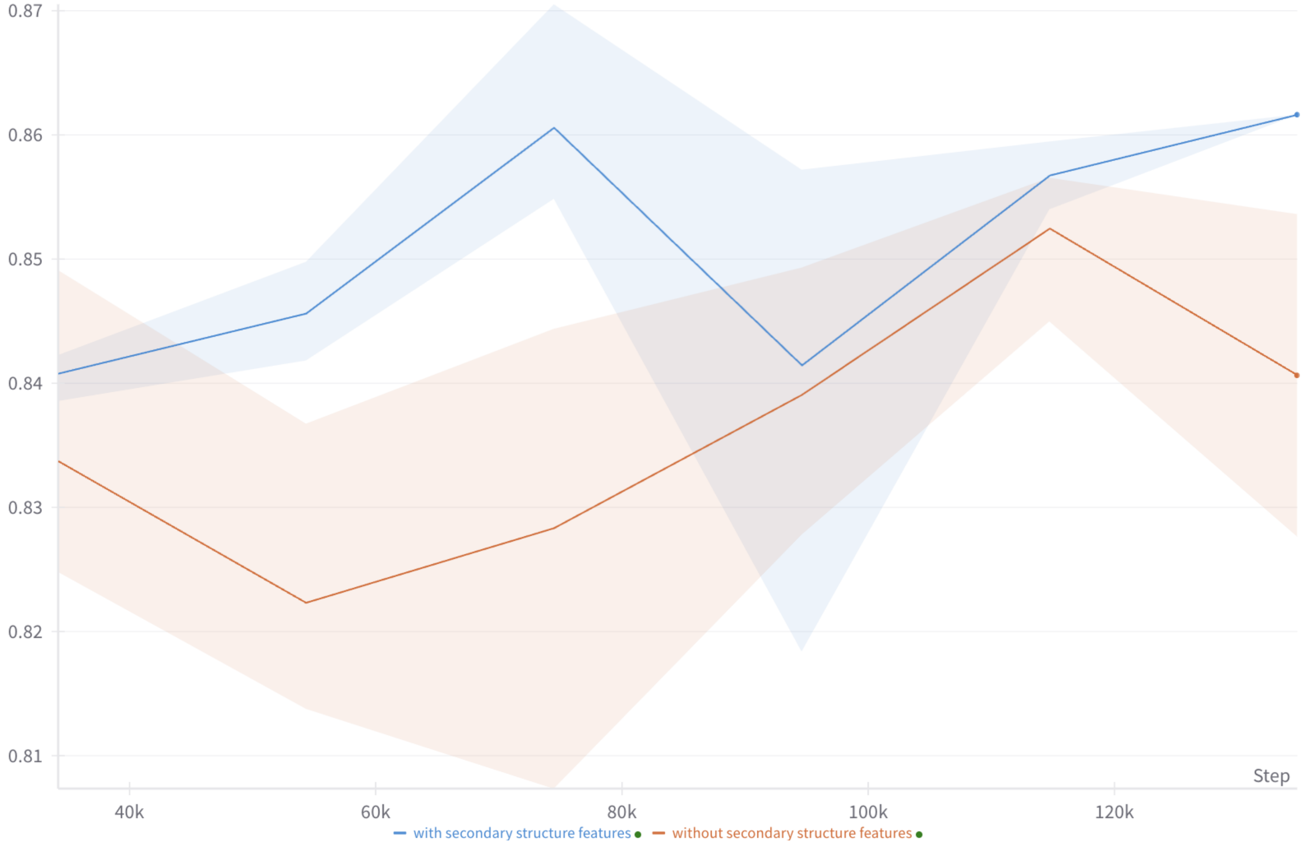

Figure 10. Results from training six Chainsaw models, three with secondary structure features included and three without (distance matrix only). We show the grouped mean IoU score on the validation data. The shaded range covers the minimum and maximum values at each epoch.

D.4 Ablation analysis: alpha distances versus beta distances

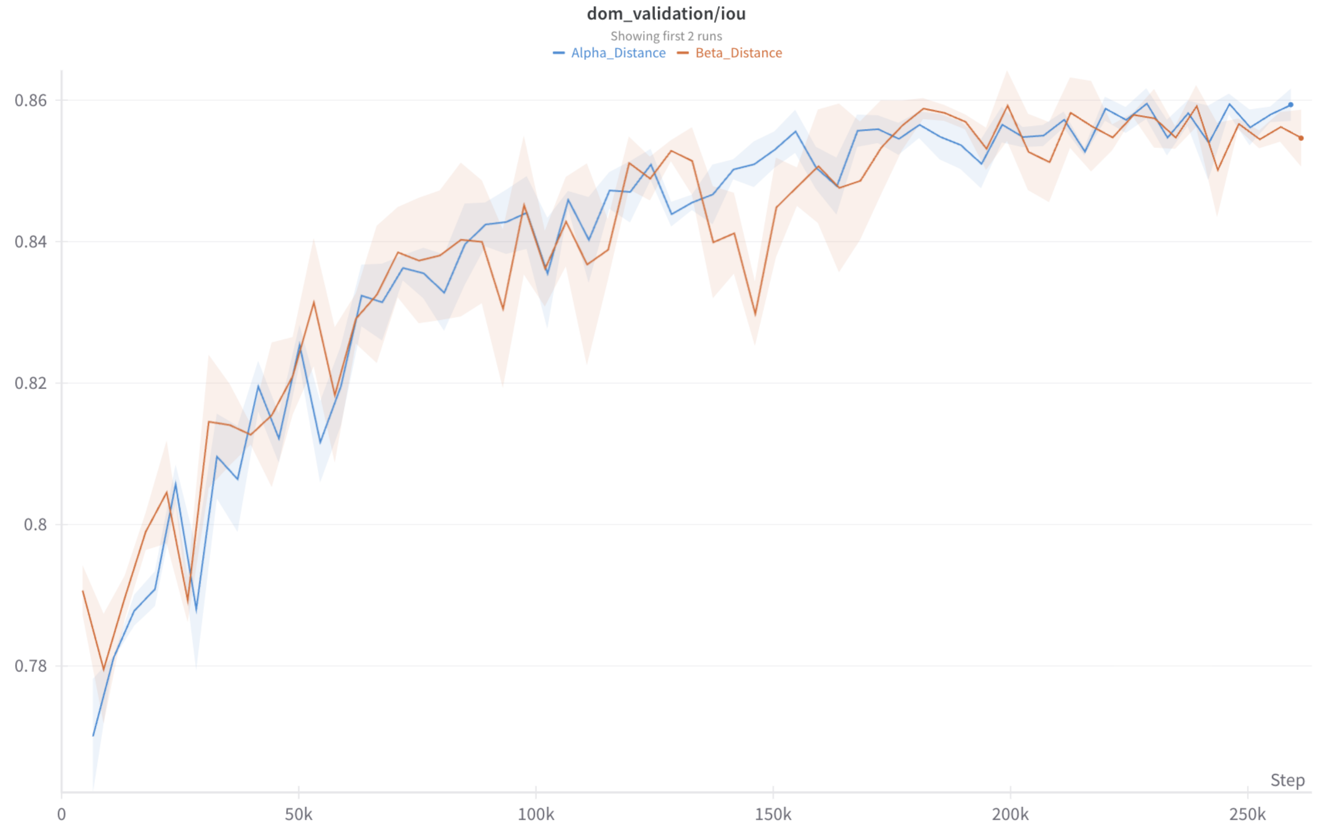

Figure 11. Results from training six Chainsaw models, three with α-carbon distances and three with β-carbon distances. The shaded range covers the minimum and maximum values at each epoch.

D.5 Model confidence and accuracy

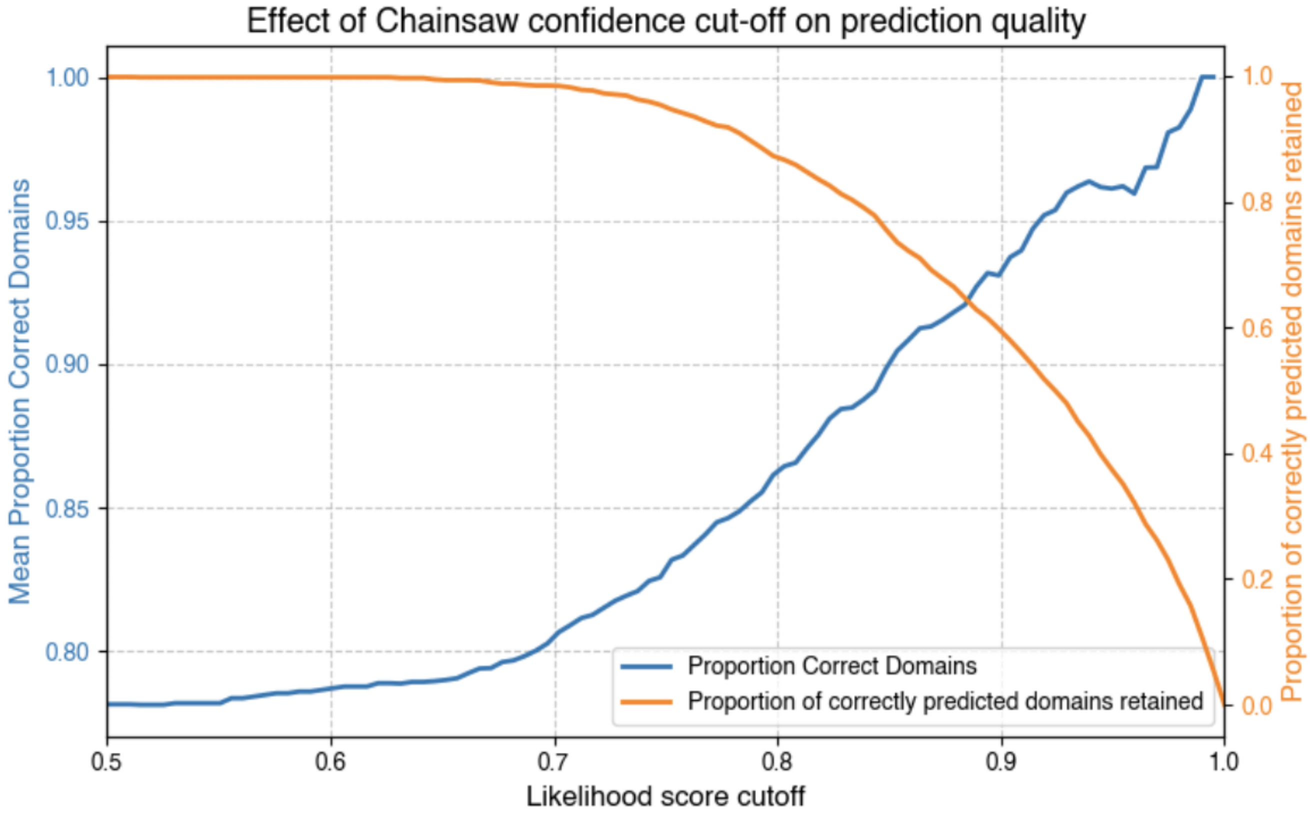

Figure 12. The Chainsaw confidence score reflects the likelihood of $\mathbf{A}'$ (final assignment) under $\hat{\mathbf{A}}$ (output of neural network). We find that this is correlated with the accuracy of the predictions and can therefore be used as a filter to increase the precision of Chainsaw domain predictions albeit at the expense of recall.

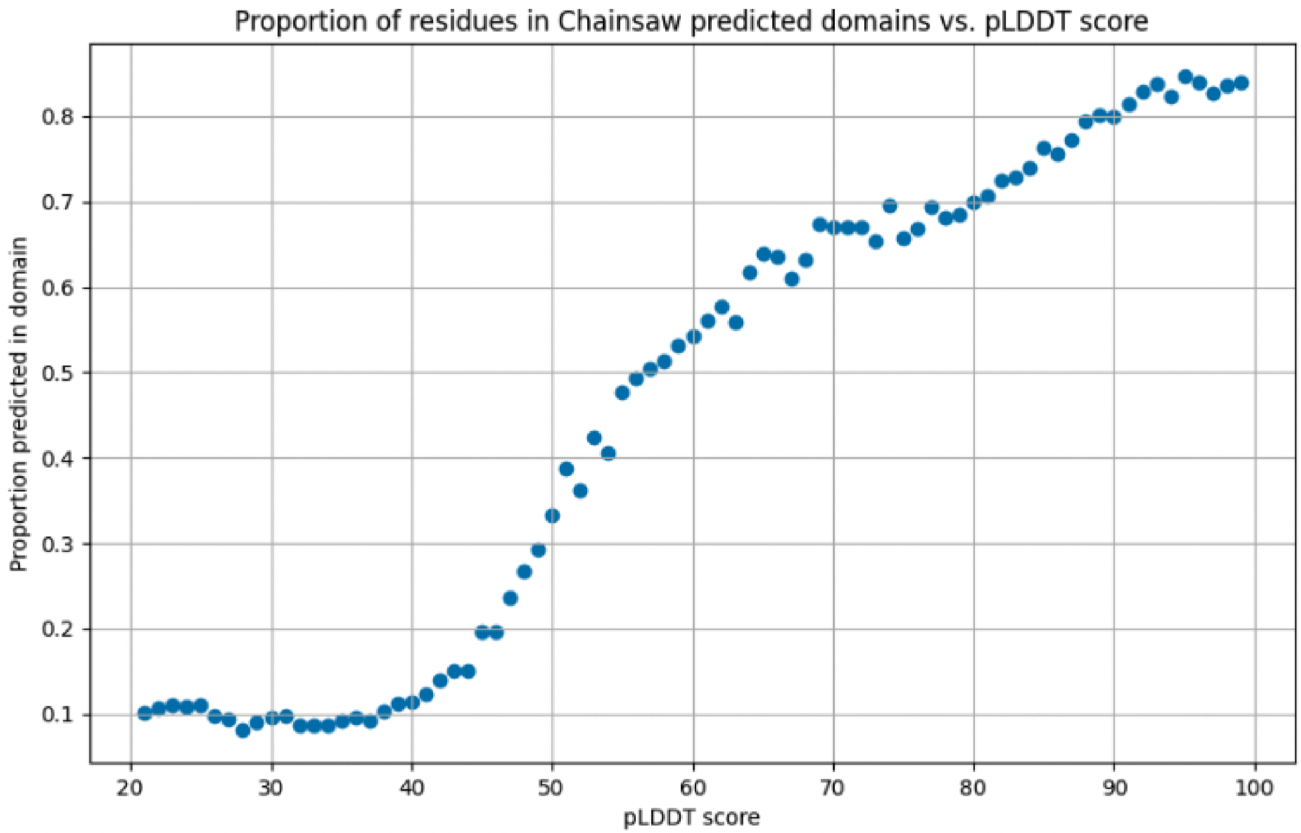

Figure 13. We calculate the proportion of residues that are predicted to be in a domain for each binned pLDDT score. Results were generated for 200 random AFDB models from the human proteome.

Chainsaw vs. UniDoc 200 human blind comparison

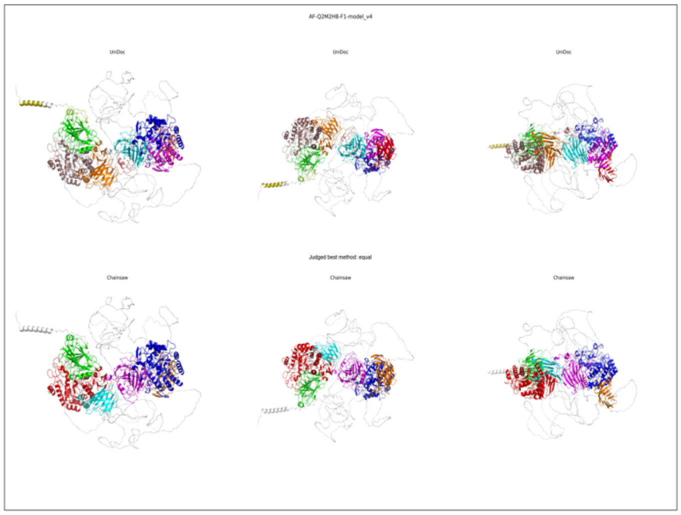

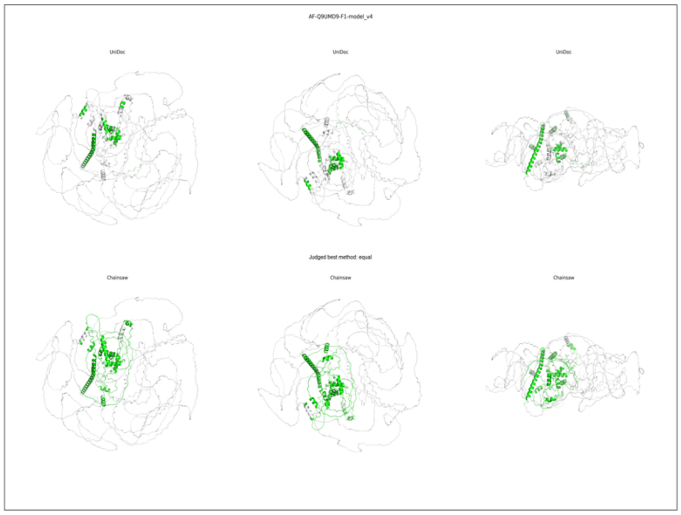

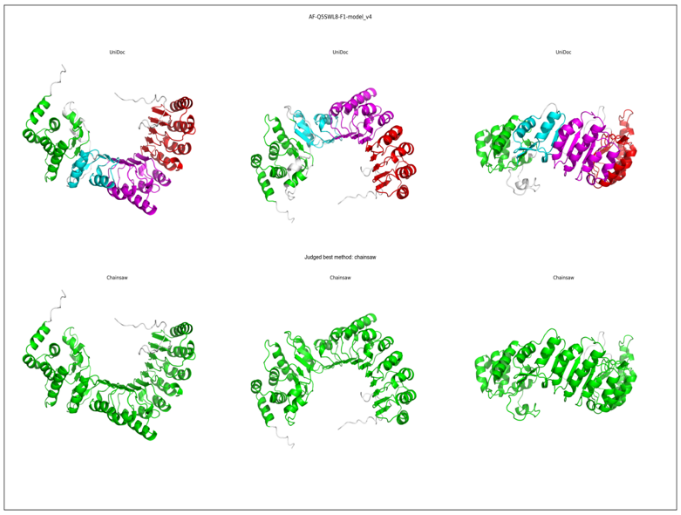

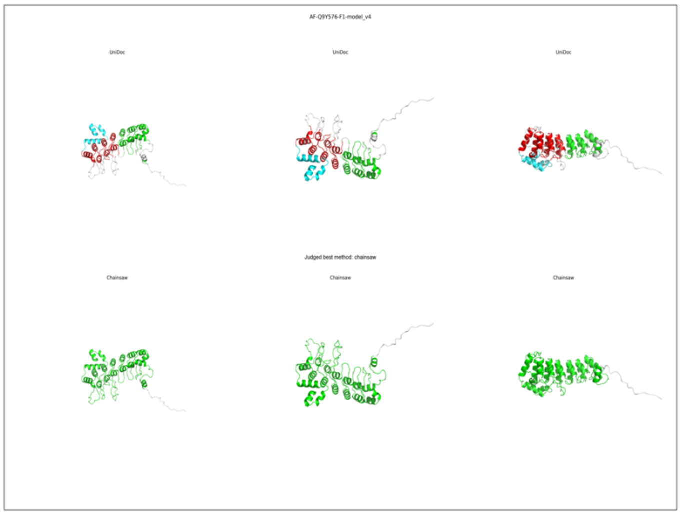

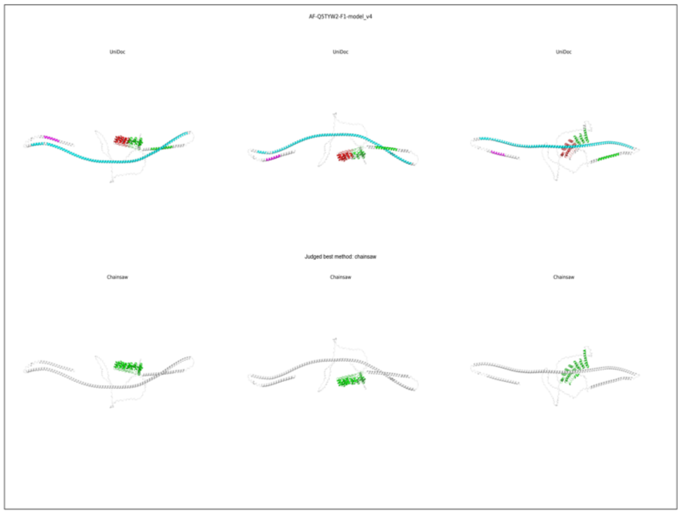

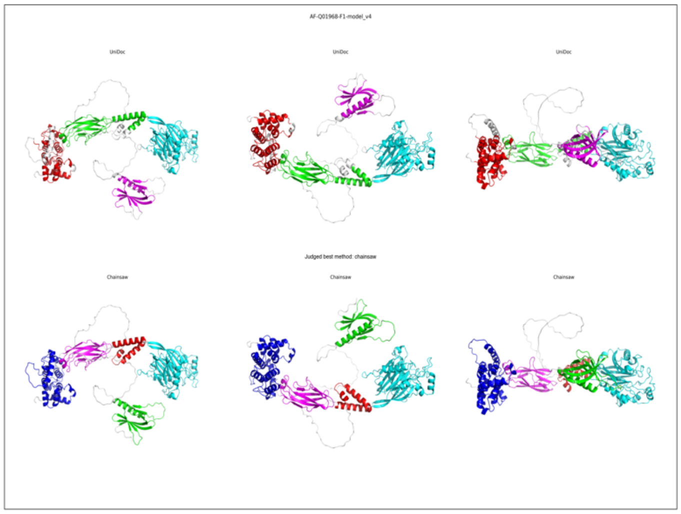

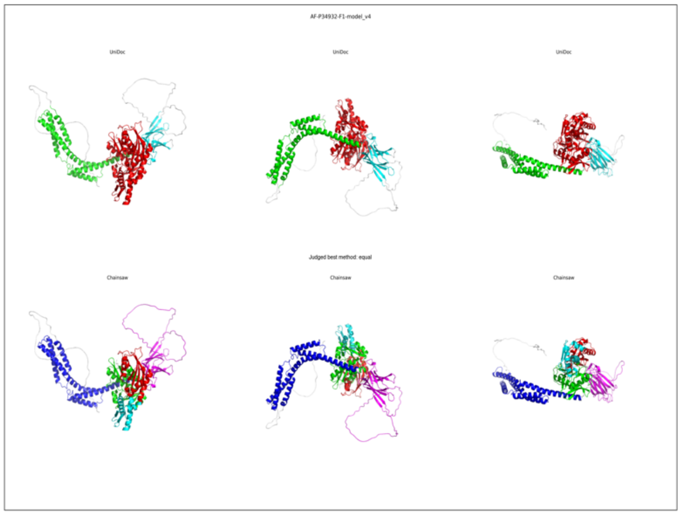
AF-P34932-F1-model_v4
UniDoc
UniDoc
UniDoc
Judged best method: equal
Chainsaw
Chainsaw
Chainsaw

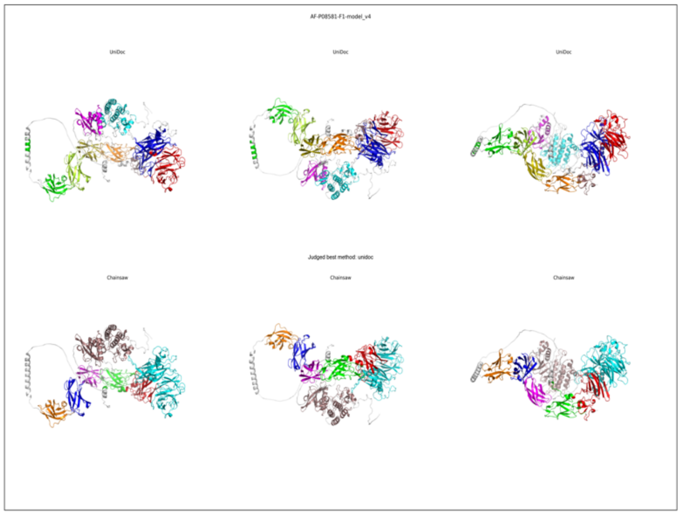
AF-P08581-F1-model_v4
UniDoc
UniDoc
UniDoc
Judged best method: unidoc
Chainsaw
Chainsaw
Chainsaw

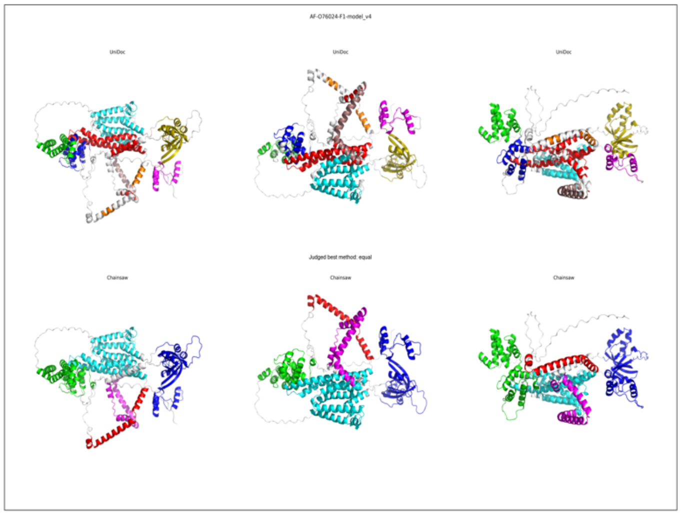
AF-O76024-F1-model_v4
UniDoc
UniDoc
UniDoc
Judged best method: equal
Chainsaw
Chainsaw
Chainsaw

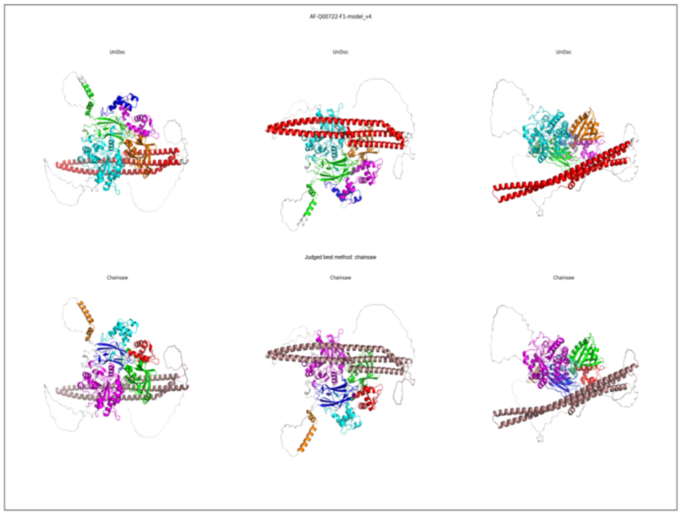
AF-Q00722-F1-model_v4
UniDoc
UniDoc
UniDoc
Judged best method: chainsaw
Chainsaw
Chainsaw
Chainsaw

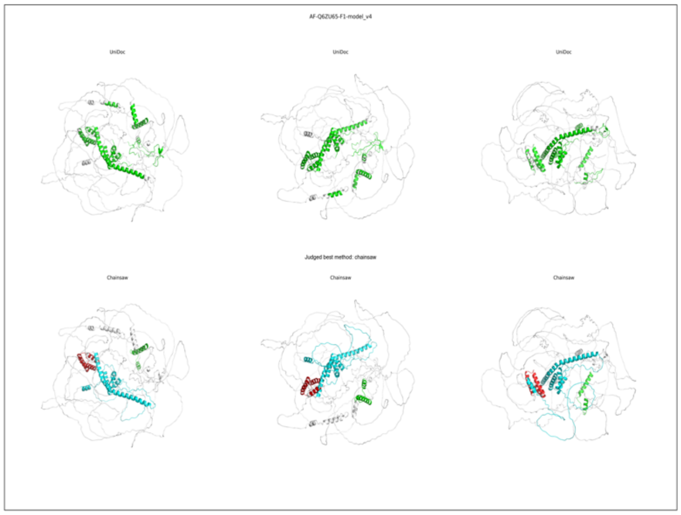
AF-Q6ZU65-F1-model_v4
UniDoc
UniDoc
UniDoc
Judged best method: chainsaw
Chainsaw
Chainsaw
Chainsaw

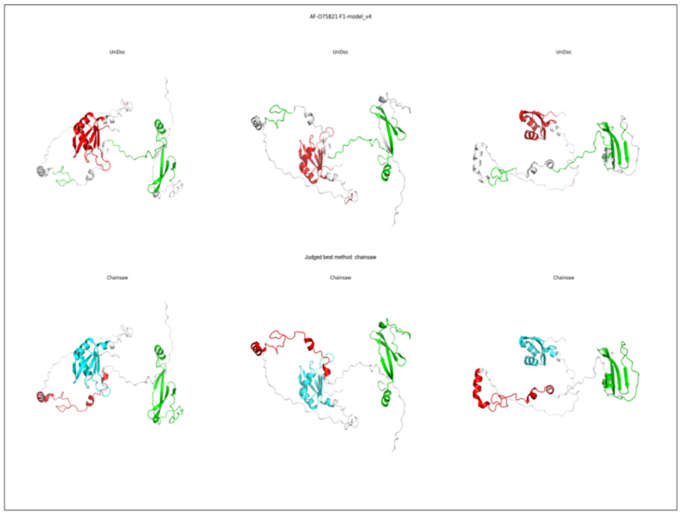
AF-O75821-F1-model_v4
UniDoc
UniDoc
UniDoc
Judged best method: chainsaw
Chainsaw
Chainsaw
Chainsaw

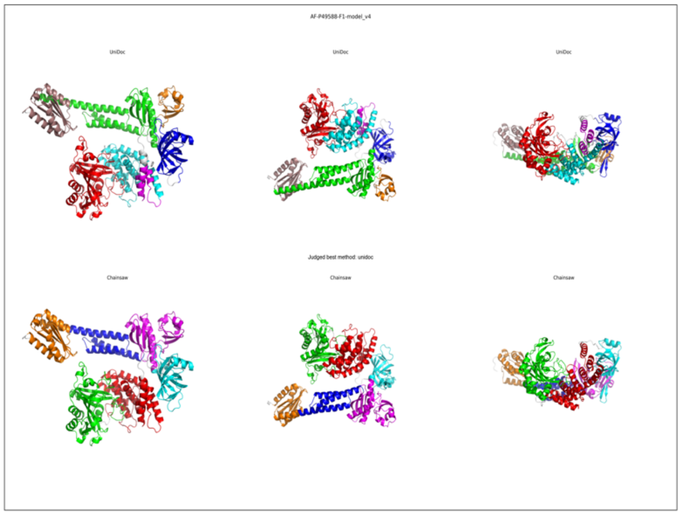
AF-P49588-F1-model_v4
UniDoc
UniDoc
UniDoc
Judged best method: unidoc
Chainsaw
Chainsaw
Chainsaw

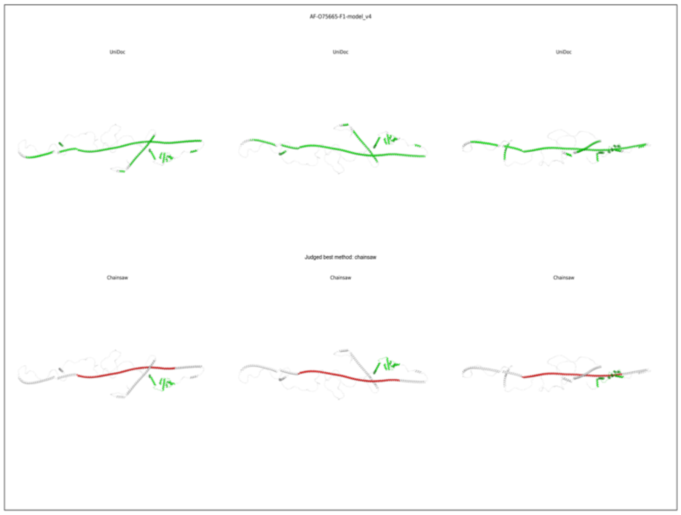
AF-O75665-F1-model_v4
UniDoc
UniDoc
UniDoc
Judged best method: chainsaw
Chainsaw
Chainsaw
Chainsaw

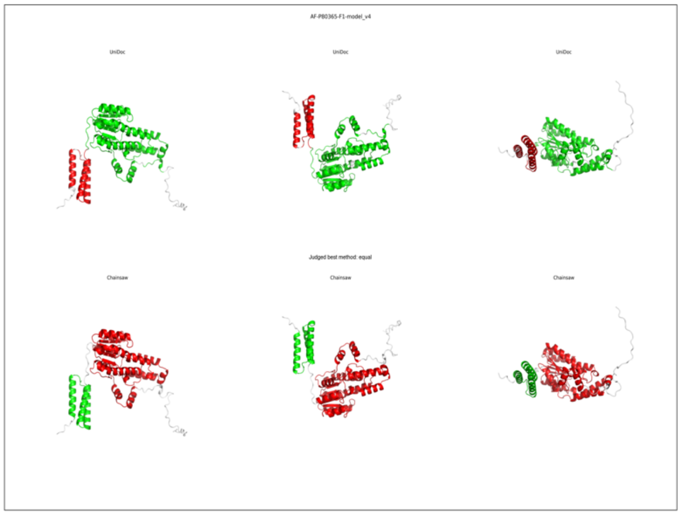
AF-P80365-F1-model_v4
UniDoc
UniDoc
UniDoc
Judged best method: equal
Chainsaw
Chainsaw
Chainsaw

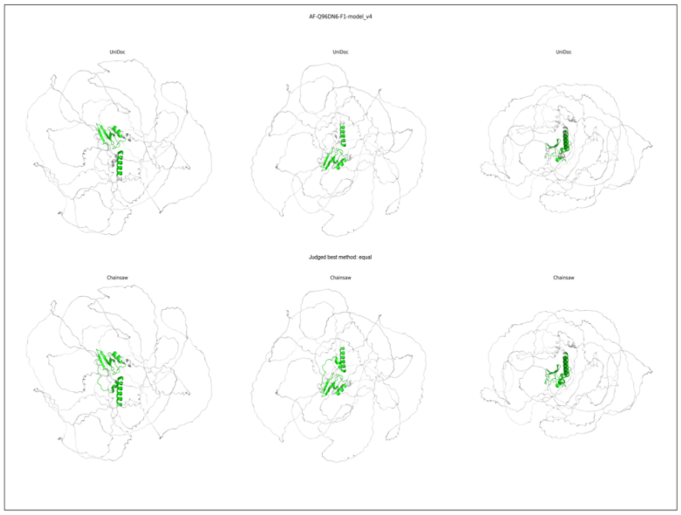
AF-Q96DN6-F1-model_v4
UniDoc
UniDoc
UniDoc
Judged best method: equal
Chainsaw
Chainsaw
Chainsaw

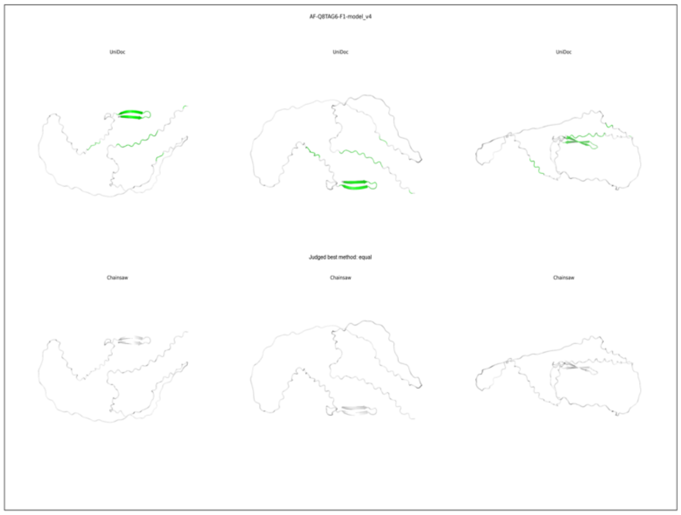
AF-Q8TAG6-F1-model_v4
UniDoc
UniDoc
UniDoc
Judged best method: equal
Chainsaw
Chainsaw
Chainsaw

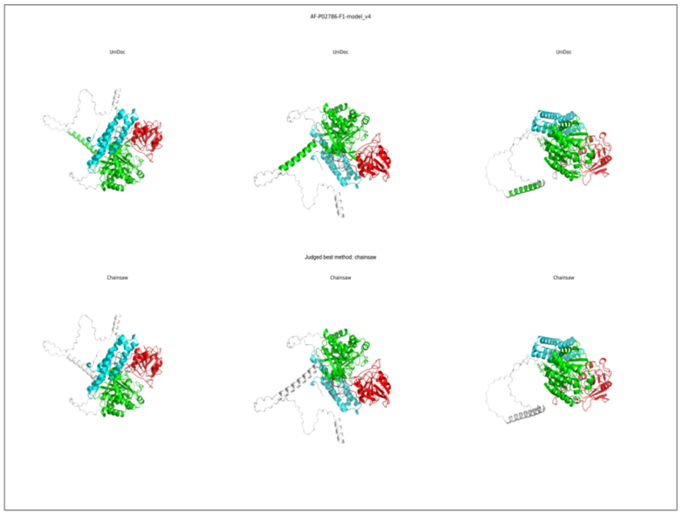
AF-P02786-F1-model_v4
UniDoc
UniDoc
UniDoc
Judged best method: chainsaw
Chainsaw
Chainsaw
Chainsaw

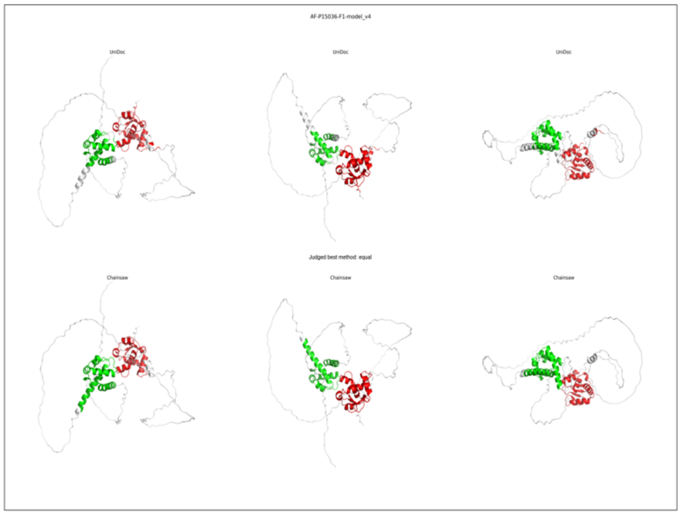
AF-P15036-F1-model_v4
UniDoc
UniDoc
UniDoc
Judged best method: equal
Chainsaw
Chainsaw
Chainsaw

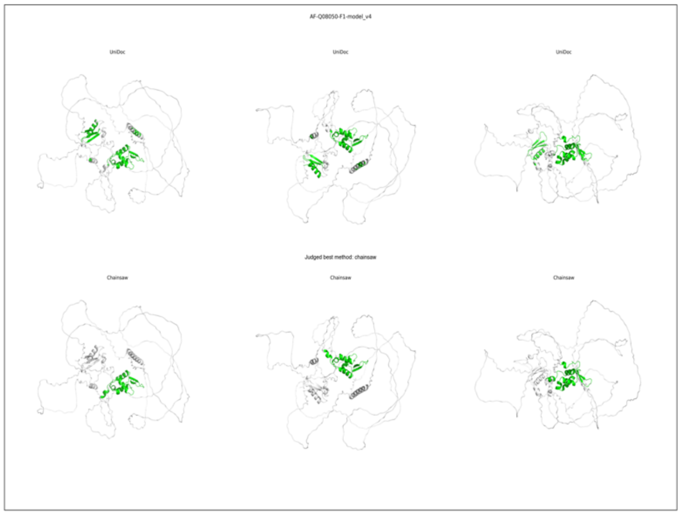
AF-Q08050-F1-model_v4
UniDoc
UniDoc
UniDoc
Judged best method: chainsaw
Chainsaw
Chainsaw
Chainsaw

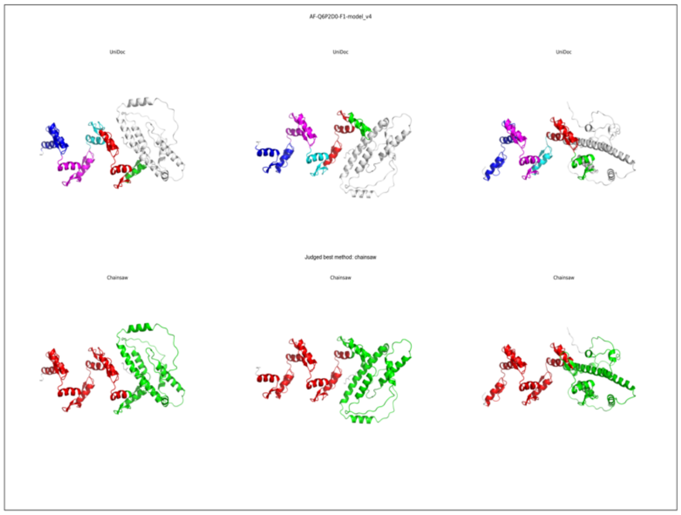
AF-Q6P2D0-F1-model_v4
UniDoc
UniDoc
UniDoc
Judged best method: chainsaw
Chainsaw
Chainsaw
Chainsaw

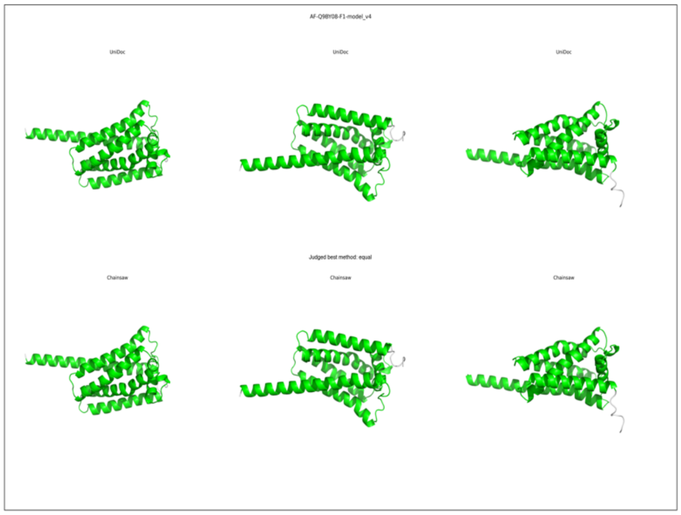
AF-Q9BY08-F1-model_v4
UniDoc
UniDoc
UniDoc
Judged best method: equal
Chainsaw
Chainsaw
Chainsaw

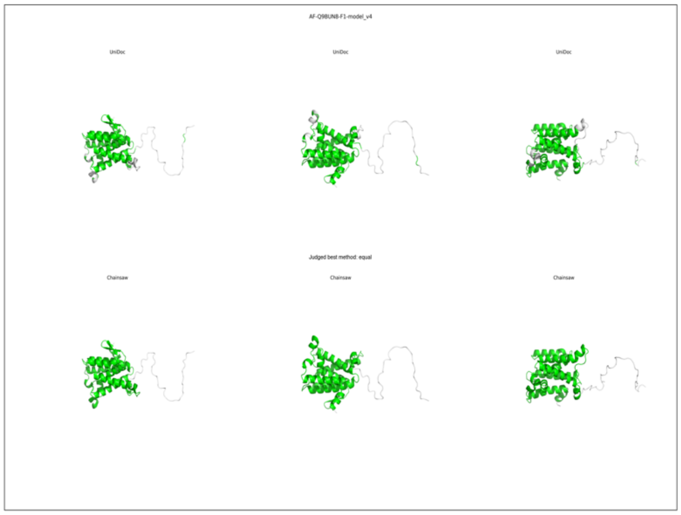
AF-Q9BUN8-F1-model_v4
UniDoc
UniDoc
UniDoc
Judged best method: equal
Chainsaw
Chainsaw
Chainsaw

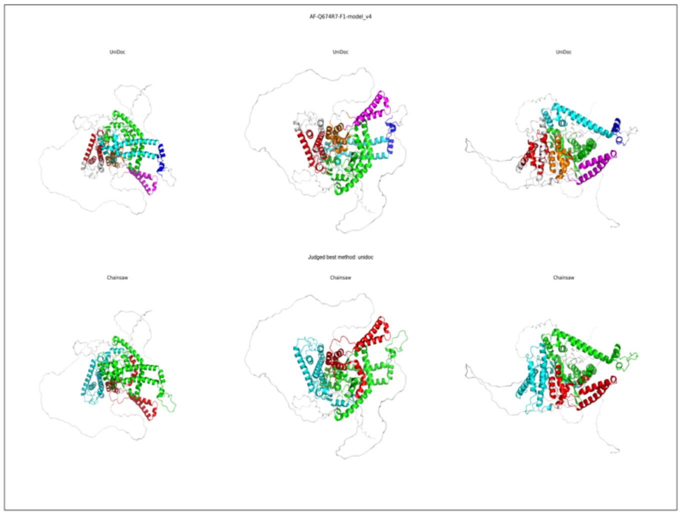
AF-Q674R7-F1-model_v4
UniDoc
UniDoc
UniDoc
Judged best method: unidoc
Chainsaw
Chainsaw
Chainsaw

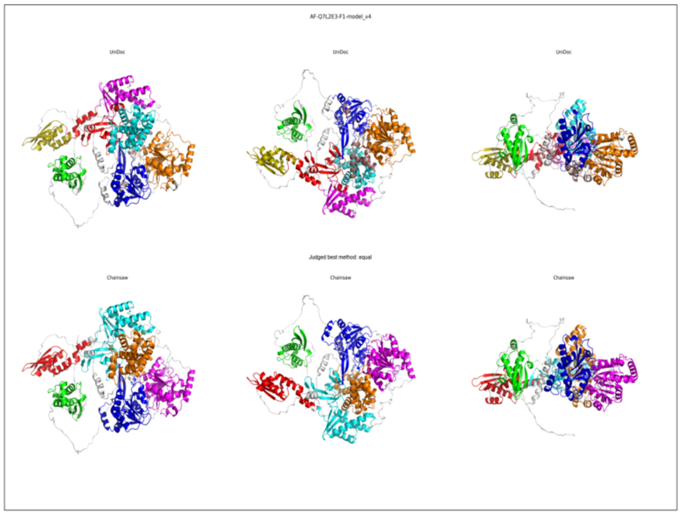
AF-Q7L2E3-F1-model_v4
UniDoc
UniDoc
UniDoc
Judged best method: equal
Chainsaw
Chainsaw
Chainsaw

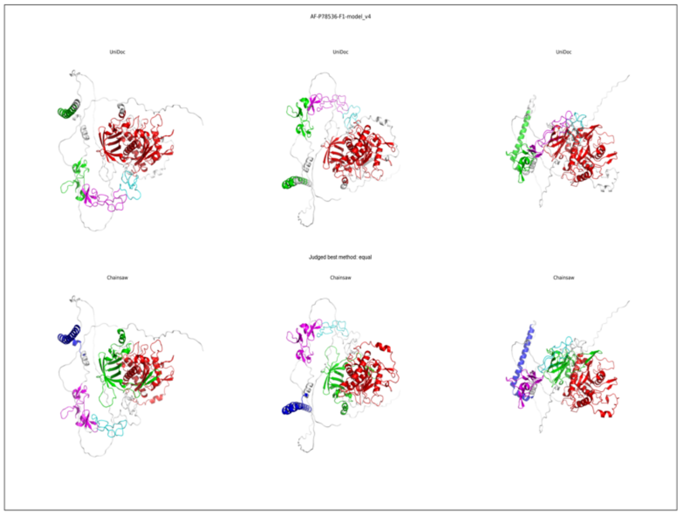
AF-P78536-F1-model_v4
UniDoc
UniDoc
UniDoc
Judged best method: equal
Chainsaw
Chainsaw
Chainsaw

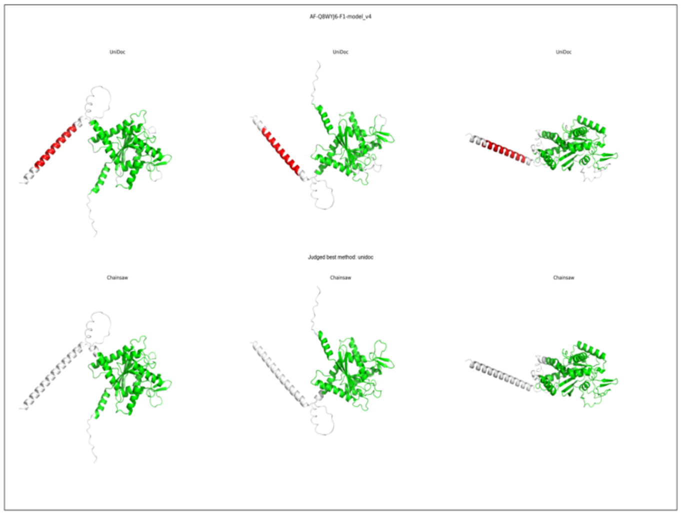
AF-Q8WYJ6-F1-model_v4
UniDoc
UniDoc
UniDoc
Judged best method: unidoc
Chainsaw
Chainsaw
Chainsaw

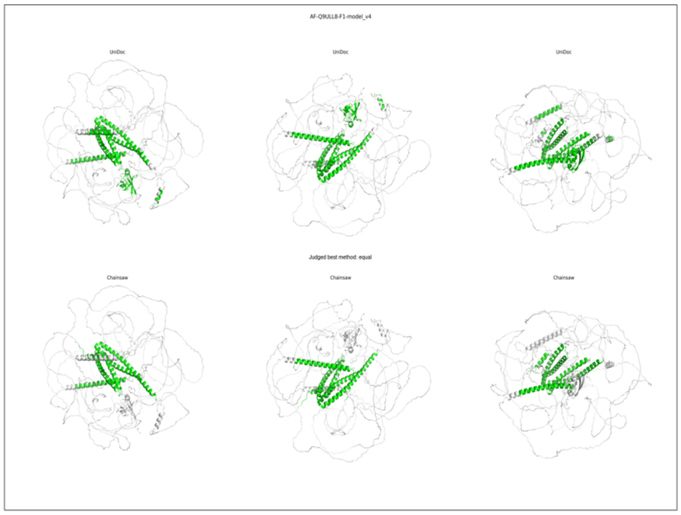
AF-Q9ULL8-F1-model_v4
UniDoc
UniDoc
UniDoc
Judged best method: equal
Chainsaw
Chainsaw
Chainsaw

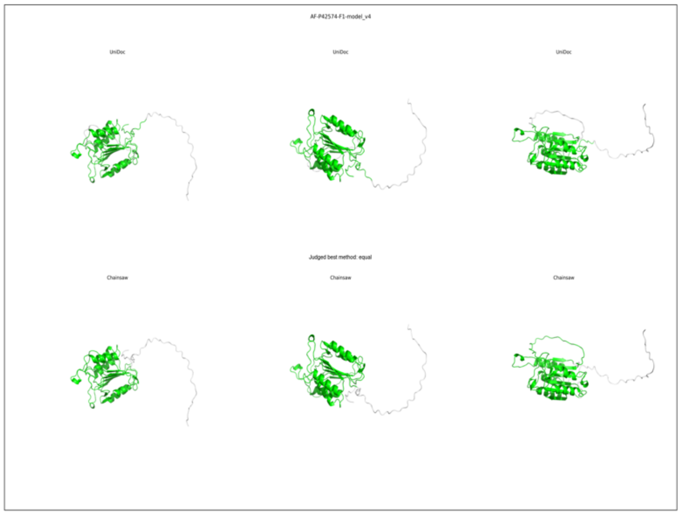
AF-P42574-F1-model_v4
UniDoc
UniDoc
UniDoc
Judged best method: equal
Chainsaw
Chainsaw
Chainsaw

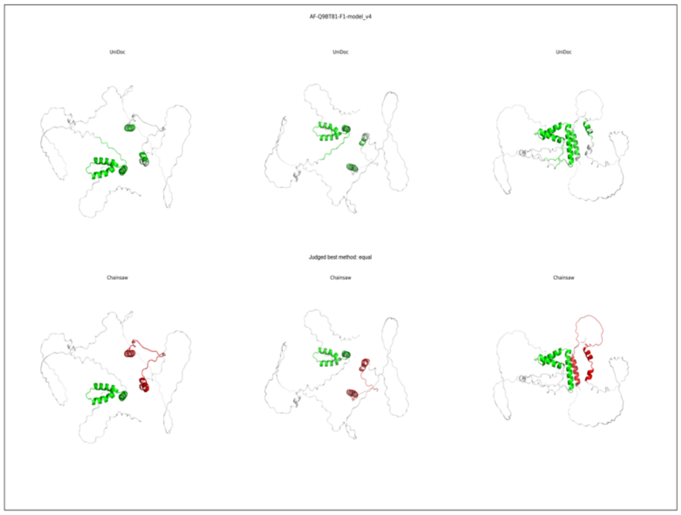
AF-Q9BT81-F1-model_v4
UniDoc
UniDoc
UniDoc
Judged best method: equal
Chainsaw
Chainsaw
Chainsaw

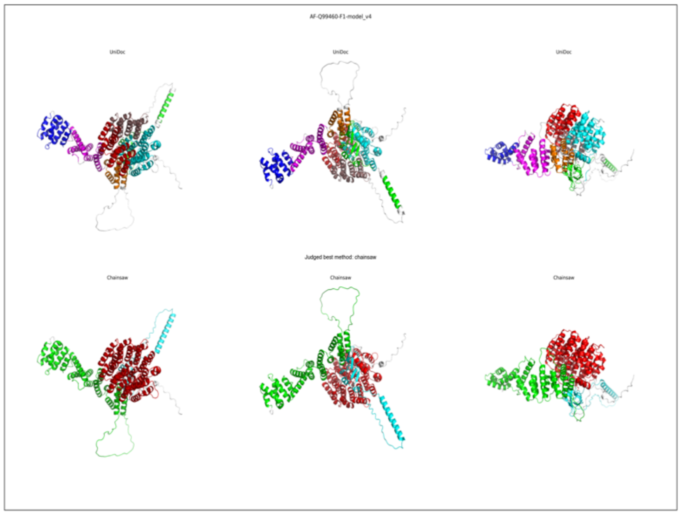
AF-Q99460-F1-model_v4
UniDoc
UniDoc
UniDoc
Judged best method: chainsaw
Chainsaw
Chainsaw
Chainsaw

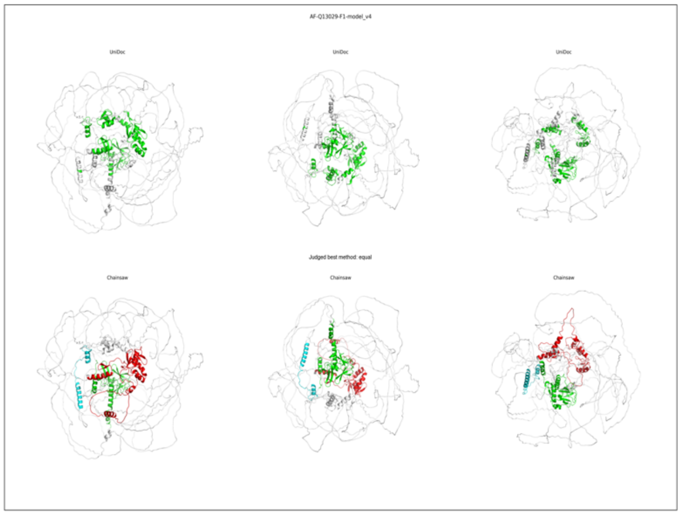
AF-Q13029-F1-model_v4
UniDoc
UniDoc
UniDoc
Judged best method: equal
Chainsaw
Chainsaw
Chainsaw

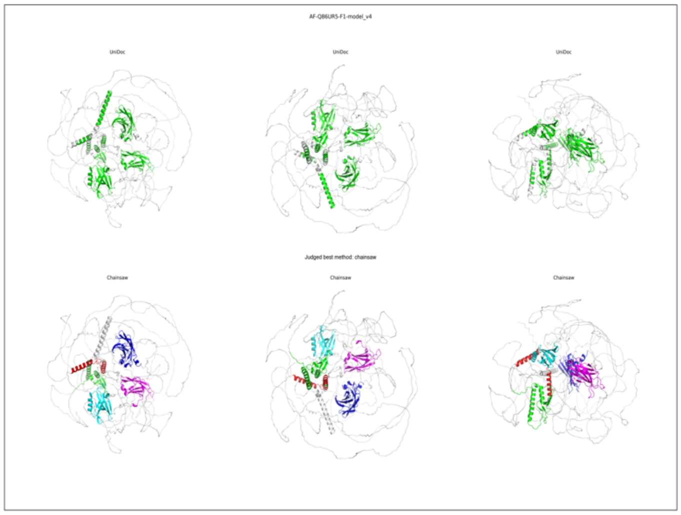
AF-Q86UR5-F1-model_v4
UniDoc
UniDoc
UniDoc
Judged best method: chainsaw
Chainsaw
Chainsaw
Chainsaw

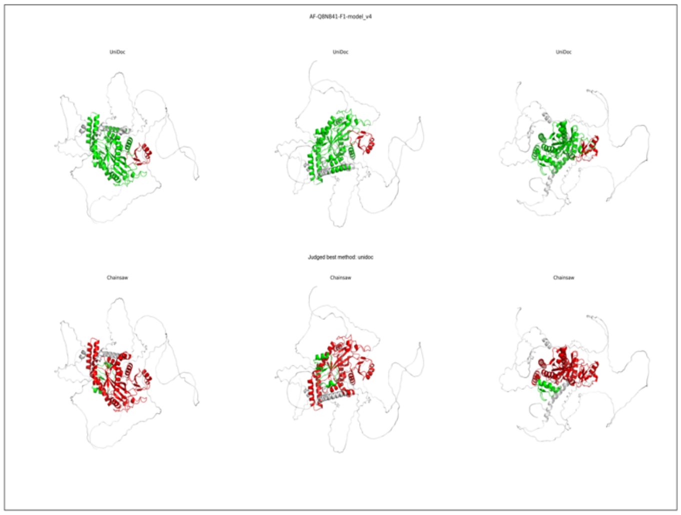
AF-Q8N841-F1-model_v4
UniDoc
UniDoc
UniDoc
Judged best method: unidoc
Chainsaw
Chainsaw
Chainsaw

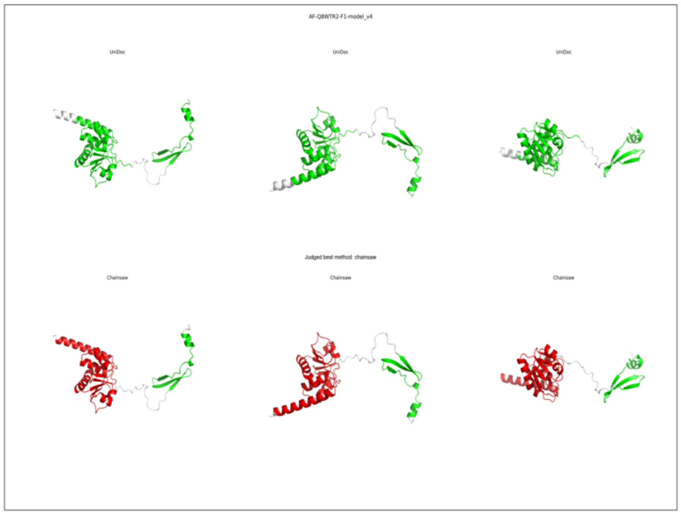
AF-Q8WTR2-F1-model_v4
UniDoc
UniDoc
UniDoc
Judged best method: chainsaw
Chainsaw
Chainsaw
Chainsaw

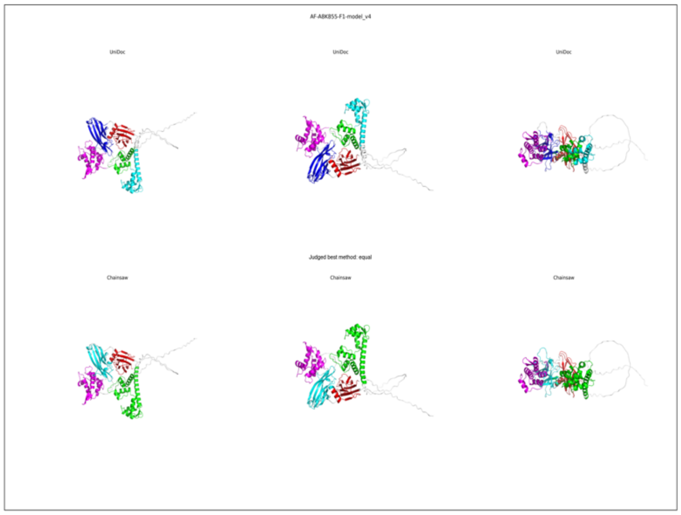
AF-A8K855-F1-model_v4
UniDoc
UniDoc
UniDoc
Judged best method: equal
Chainsaw
Chainsaw
Chainsaw

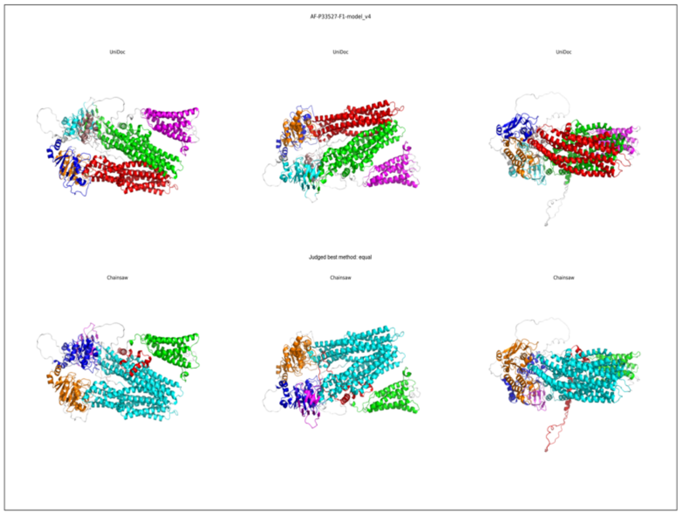
AF-P33527-F1-model_v4
UniDoc
UniDoc
UniDoc
Judged best method: equal
Chainsaw
Chainsaw
Chainsaw

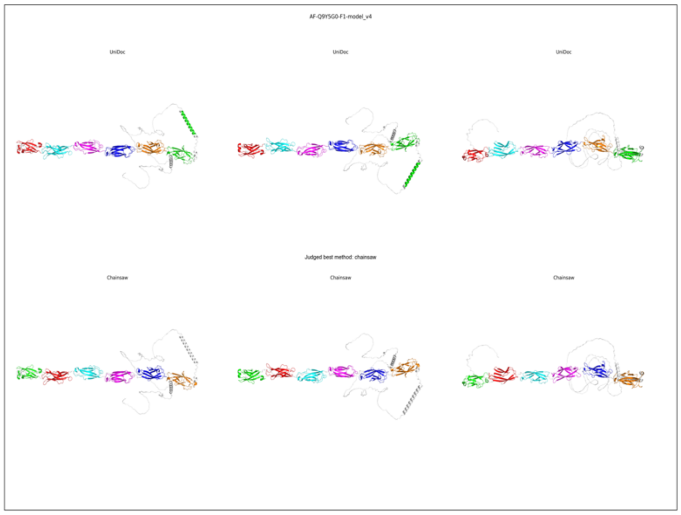
AF-Q9Y5G0-F1-model_v4
UniDoc
UniDoc
UniDoc
Judged best method: chainsaw
Chainsaw
Chainsaw
Chainsaw

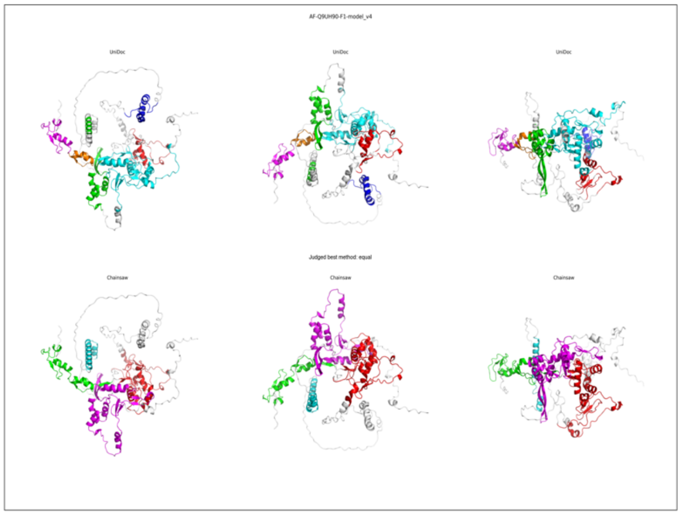
AF-Q9UH90-F1-model_v4
UniDoc
UniDoc
UniDoc
Judged best method: equal
Chainsaw
Chainsaw
Chainsaw

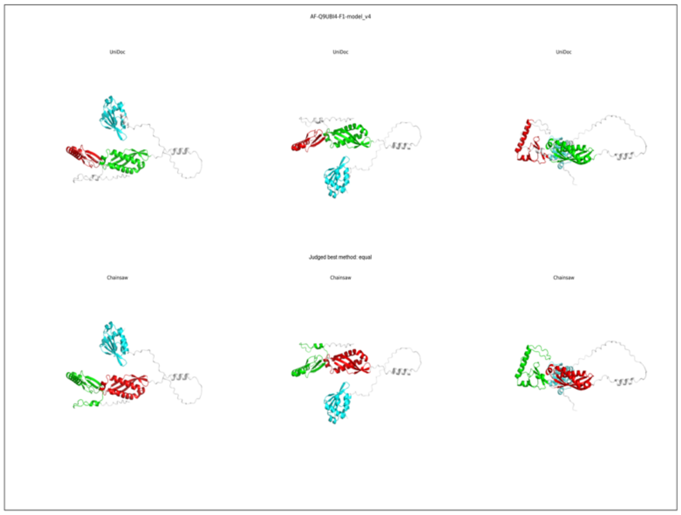
AF-Q9UBI4-F1-model_v4
UniDoc
UniDoc
UniDoc
Judged best method: equal
Chainsaw
Chainsaw
Chainsaw

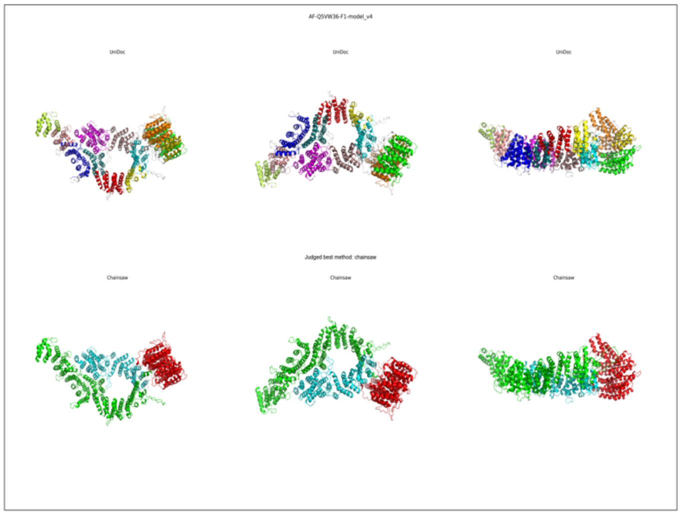
AF-Q5VW36-F1-model_v4
UniDoc
UniDoc
UniDoc
Judged best method: chainsaw
Chainsaw
Chainsaw
Chainsaw

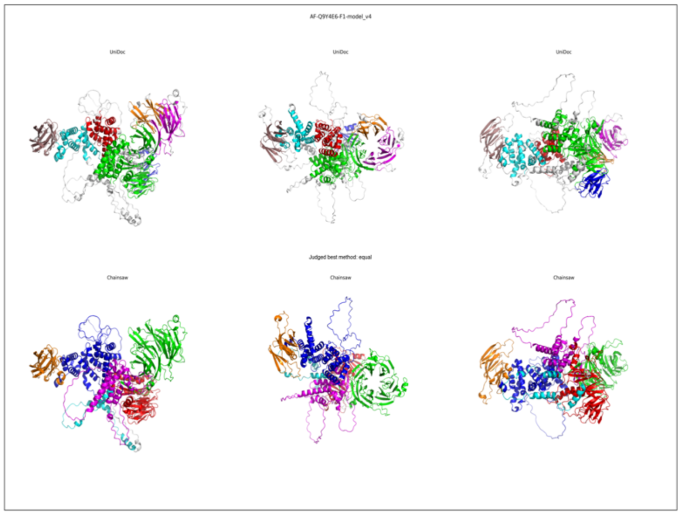
AF-Q9Y4E6-F1-model_v4
UniDoc
UniDoc
UniDoc
Judged best method: equal
Chainsaw
Chainsaw
Chainsaw

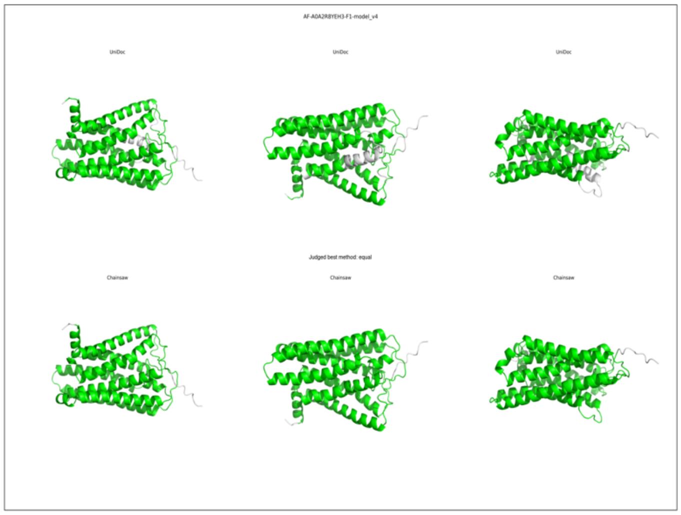
AF-A0A2R8YEH3-F1-model_v4
UniDoc
UniDoc
UniDoc
Judged best method: equal
Chainsaw
Chainsaw
Chainsaw

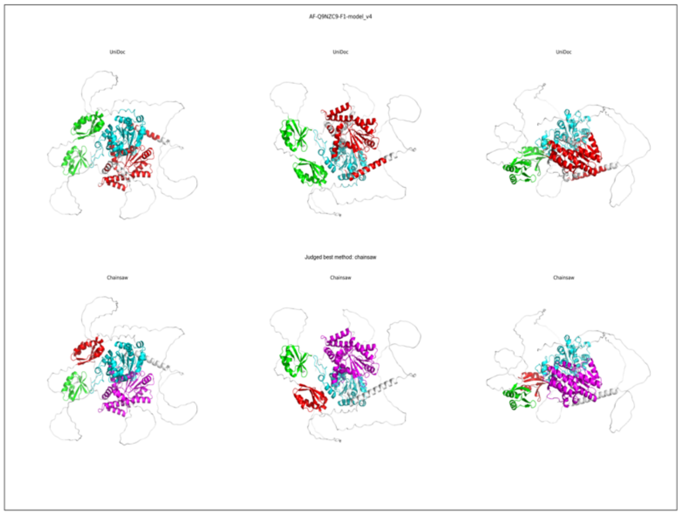
AF-Q9NZC9-F1-model_v4
UniDoc
UniDoc
UniDoc
Judged best method: chainsaw
Chainsaw
Chainsaw
Chainsaw

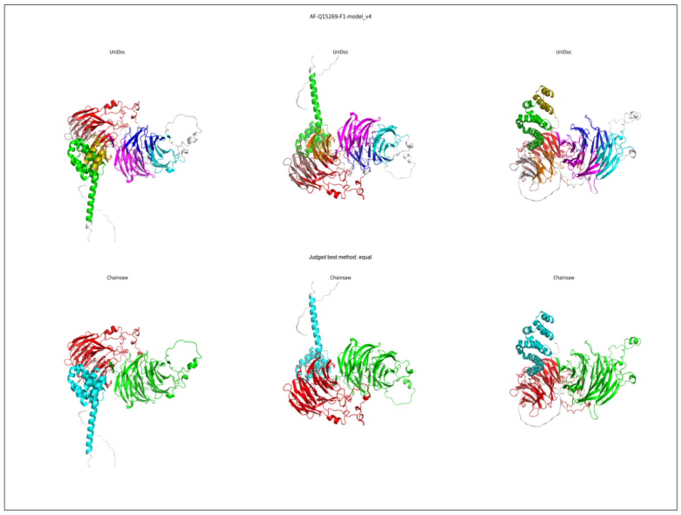
AF-Q15269-F1-model_v4
UniDoc
UniDoc
UniDoc
Judged best method: equal
Chainsaw
Chainsaw
Chainsaw

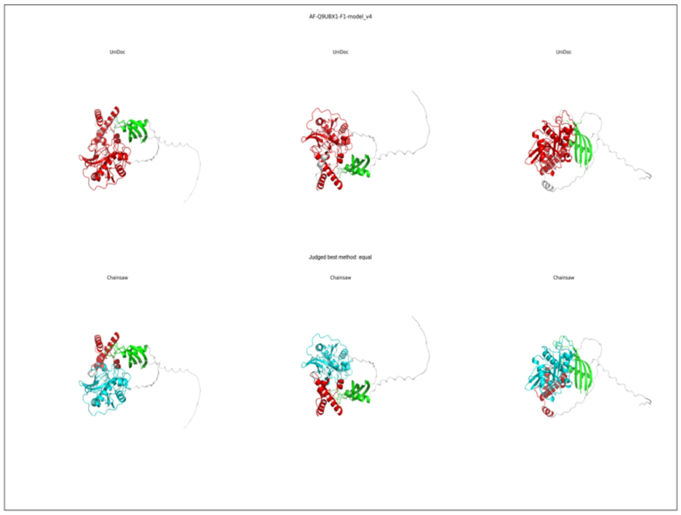
AF-Q9UBX1-F1-model_v4
UniDoc
UniDoc
UniDoc
Judged best method: equal
Chainsaw
Chainsaw
Chainsaw

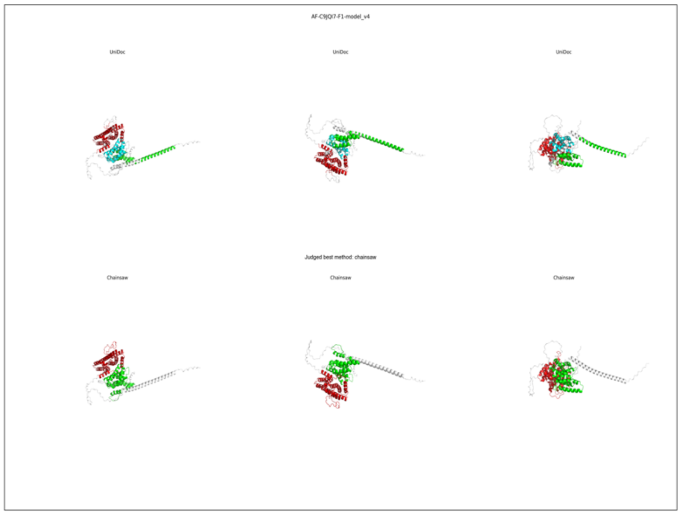
AF-C9JQI7-F1-model_v4
UniDoc
UniDoc
UniDoc
Judged best method: chainsaw
Chainsaw
Chainsaw
Chainsaw

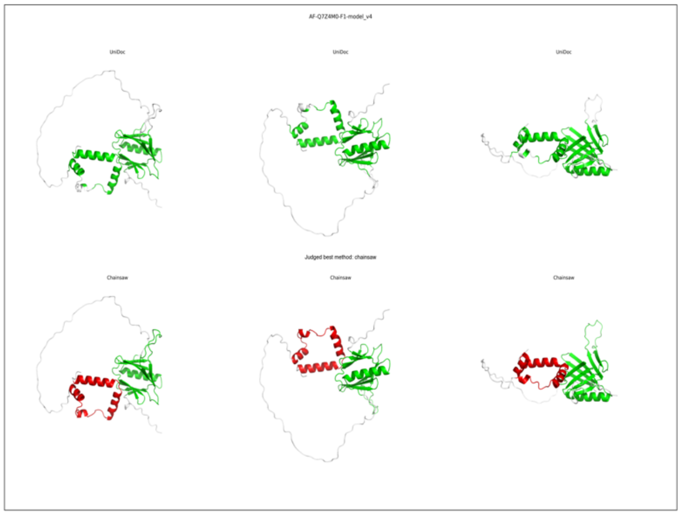
AF-Q7Z4M0-F1-model_v4
UniDoc
UniDoc
UniDoc
Judged best method: chainsaw
Chainsaw
Chainsaw
Chainsaw

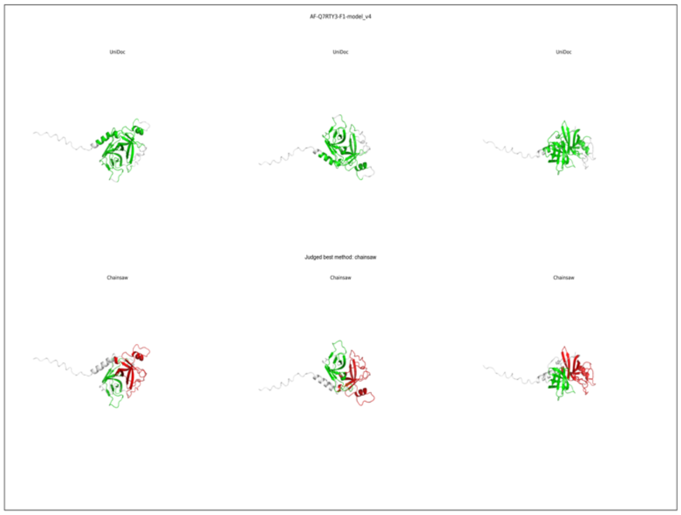
AF-Q7RTY3-F1-model_v4
UniDoc
UniDoc
UniDoc
Judged best method: chainsaw
Chainsaw
Chainsaw
Chainsaw

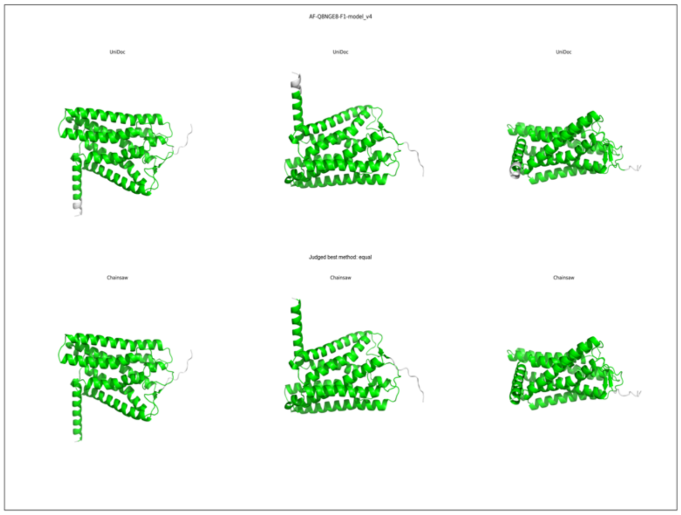
AF-Q8NGE8-F1-model_v4
UniDoc
UniDoc
UniDoc
Judged best method: equal
Chainsaw
Chainsaw
Chainsaw

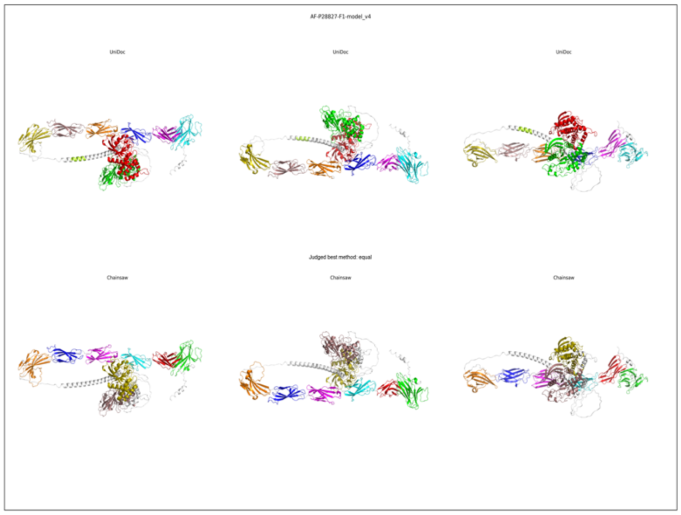
AF-P28827-F1-model_v4
UniDoc
UniDoc
UniDoc
Judged best method: equal
Chainsaw
Chainsaw
Chainsaw

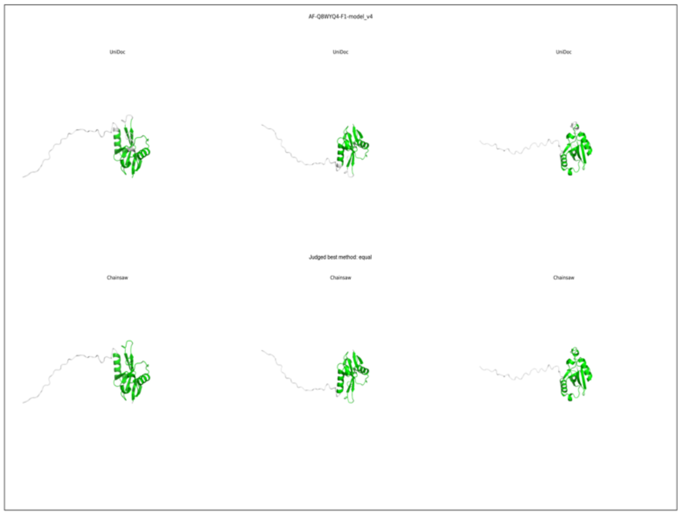
AF-Q8WYQ4-F1-model_v4
UniDoc
UniDoc
UniDoc
Judged best method: equal
Chainsaw
Chainsaw
Chainsaw

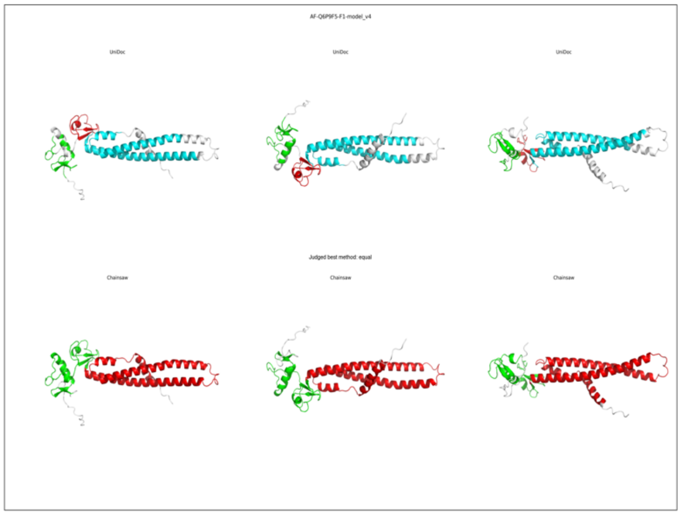
AF-Q6P9F5-F1-model_v4
UniDoc
UniDoc
UniDoc
Judged best method: equal
Chainsaw
Chainsaw
Chainsaw

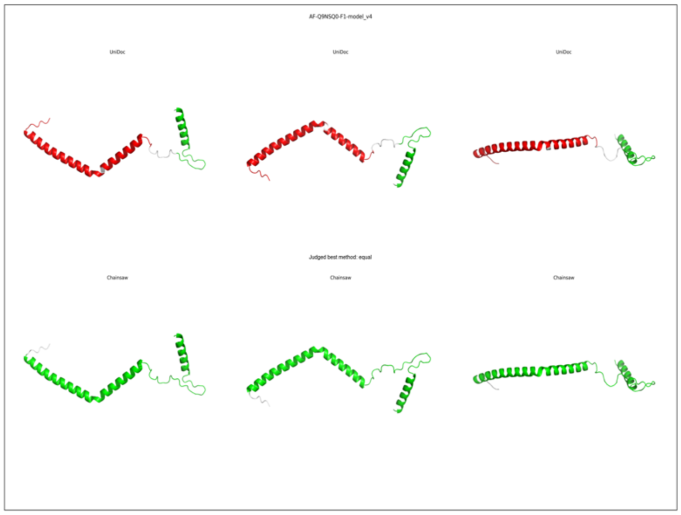
AF-Q9NSQ0-F1-model_v4
UniDoc
UniDoc
UniDoc
Judged best method: equal
Chainsaw
Chainsaw
Chainsaw

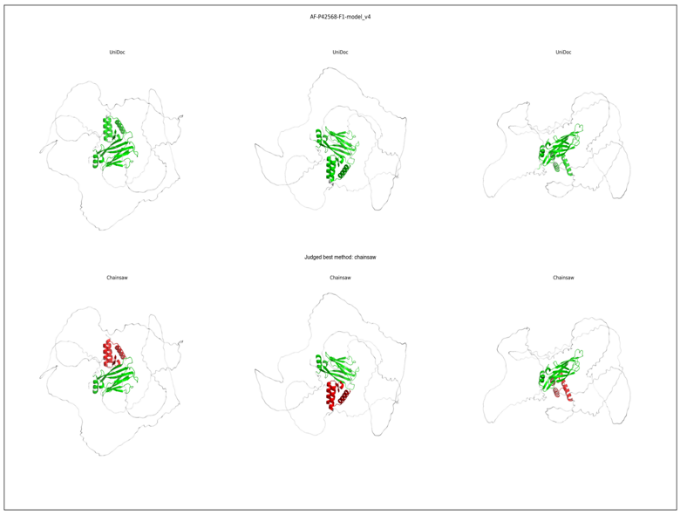
AF-P42568-F1-model_v4
UniDoc
UniDoc
UniDoc
Judged best method: chainsaw
Chainsaw
Chainsaw
Chainsaw

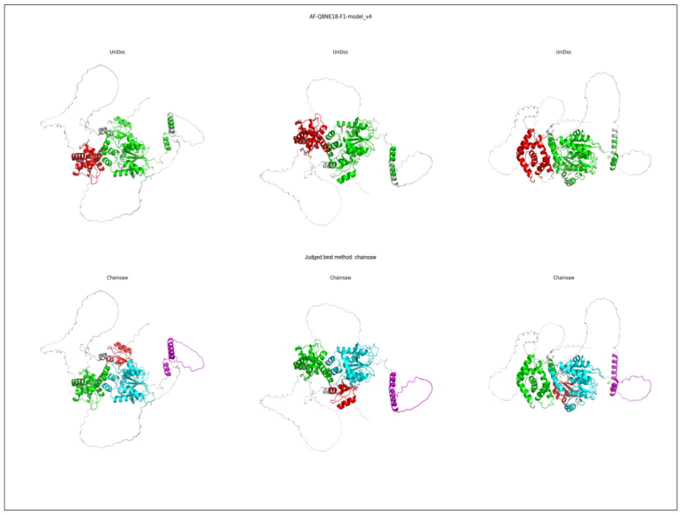
AF-Q8NE18-F1-model_v4
UniDoc
UniDoc
UniDoc
Judged best method: chainsaw
Chainsaw
Chainsaw
Chainsaw

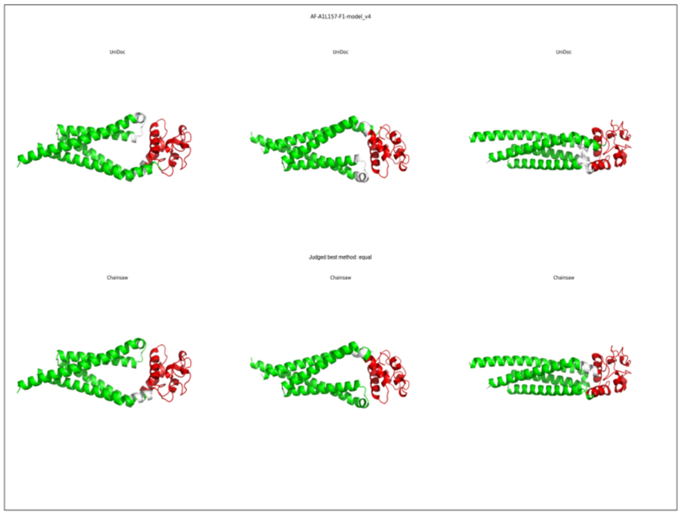
AF-A1L157-F1-model_v4
UniDoc
UniDoc
UniDoc
Judged best method: equal
Chainsaw
Chainsaw
Chainsaw

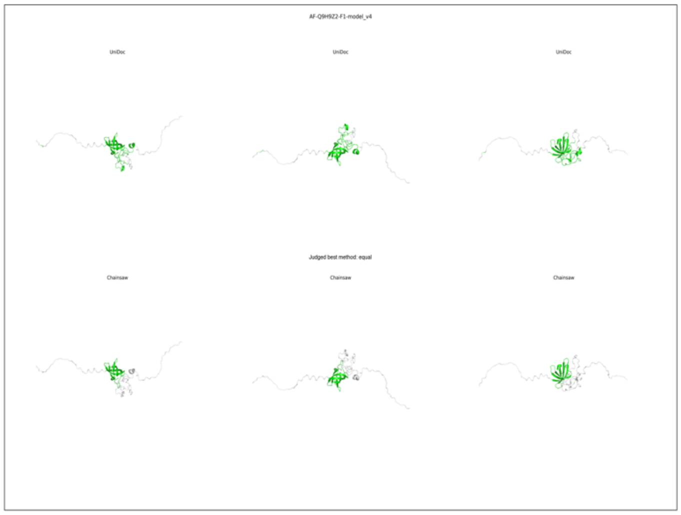
AF-Q9H9Z2-F1-model_v4
UniDoc
UniDoc
UniDoc
Judged best method: equal
Chainsaw
Chainsaw
Chainsaw

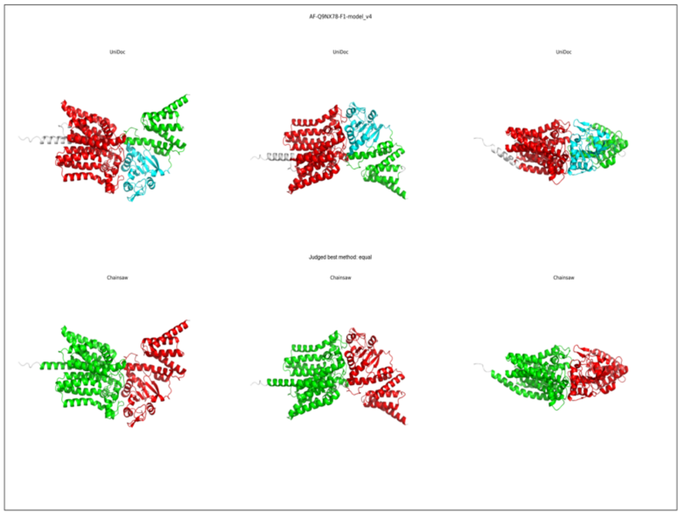
AF-Q9NX78-F1-model_v4
UniDoc
UniDoc
UniDoc
Judged best method: equal
Chainsaw
Chainsaw
Chainsaw

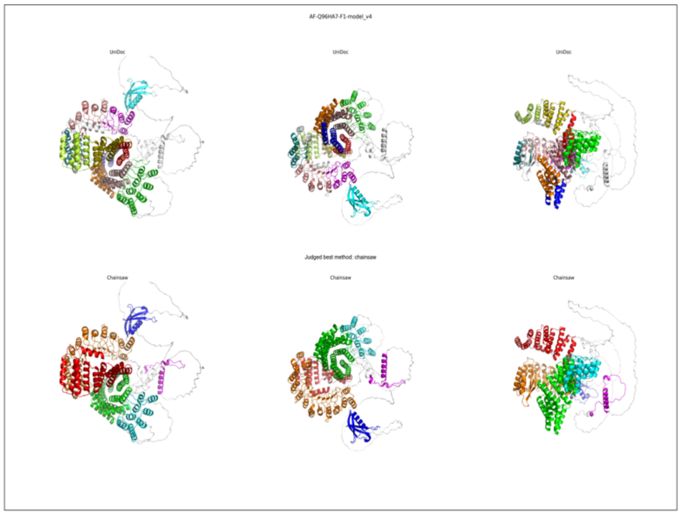
AF-Q96HA7-F1-model_v4
UniDoc
UniDoc
UniDoc
Judged best method: chainsaw
Chainsaw
Chainsaw
Chainsaw

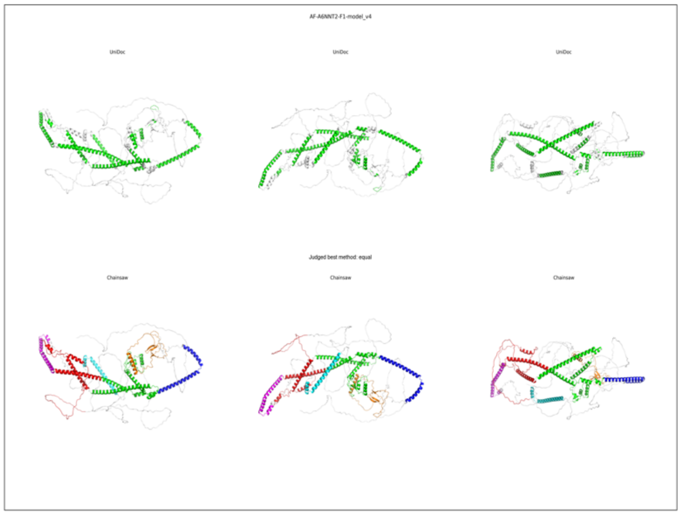
AF-A6NNT2-F1-model_v4
UniDoc
UniDoc
UniDoc
Judged best method: equal
Chainsaw
Chainsaw
Chainsaw

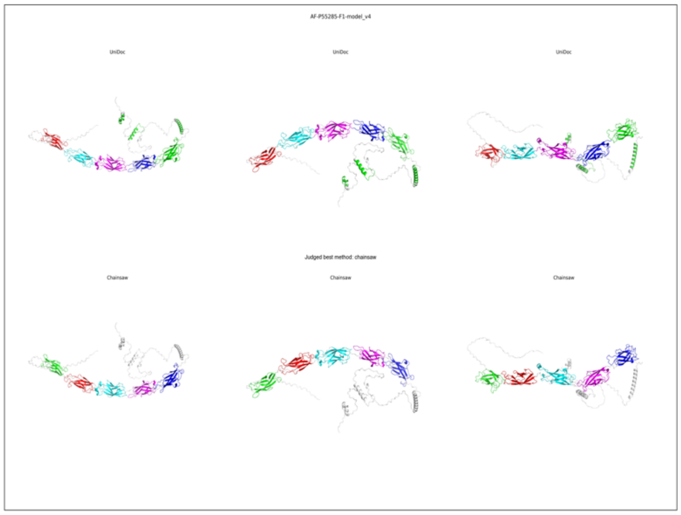
AF-P55285-F1-model_v4
UniDoc
UniDoc
UniDoc
Judged best method: chainsaw
Chainsaw
Chainsaw
Chainsaw

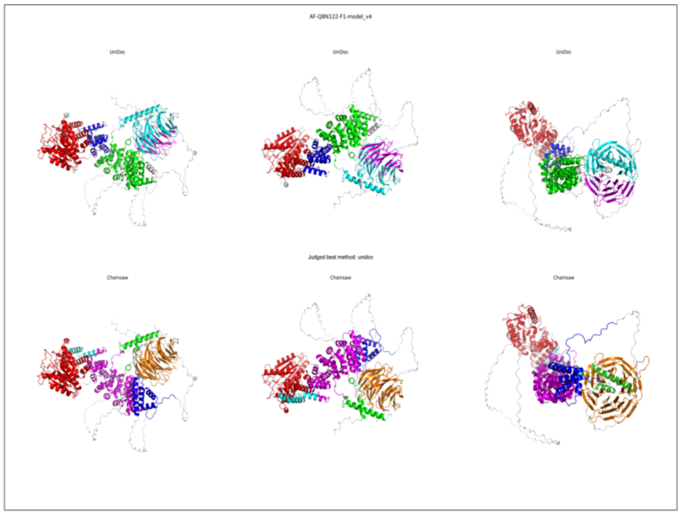
AF-Q8N122-F1-model_v4
UniDoc
UniDoc
UniDoc
Judged best method: unidoc
Chainsaw
Chainsaw
Chainsaw

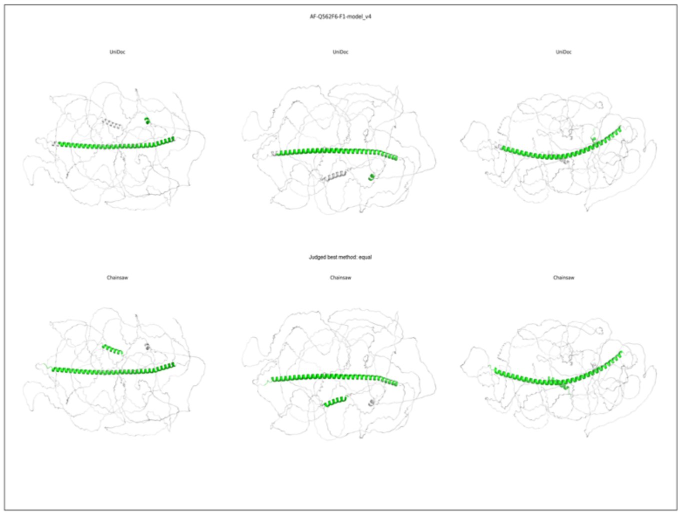
AF-Q562F6-F1-model_v4
UniDoc
UniDoc
UniDoc
Judged best method: equal
Chainsaw
Chainsaw
Chainsaw

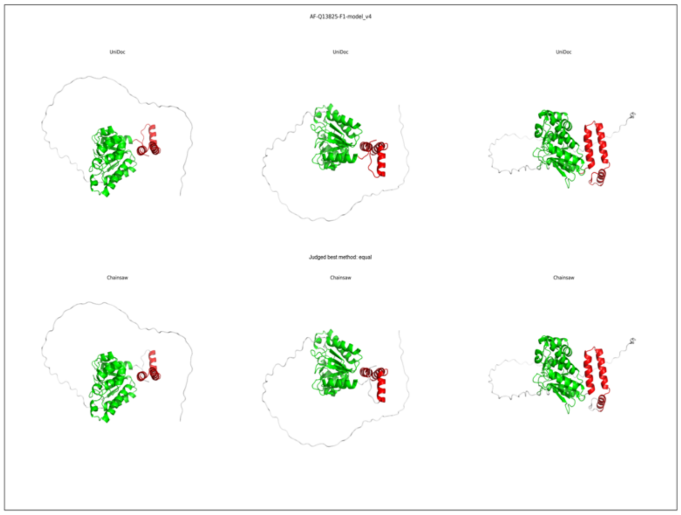
AF-Q13825-F1-model_v4
UniDoc
UniDoc
UniDoc
Judged best method: equal
Chainsaw
Chainsaw
Chainsaw

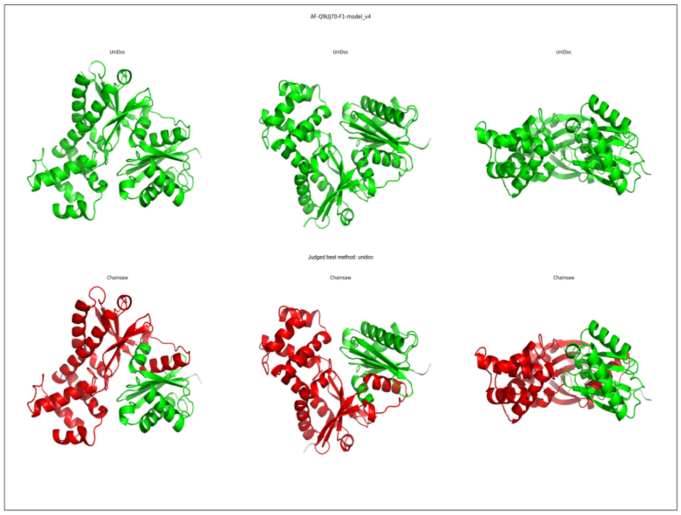
AF-Q9UJ70-F1-model_v4
UniDoc
UniDoc
UniDoc
Judged best method: unidoc
Chainsaw
Chainsaw
Chainsaw

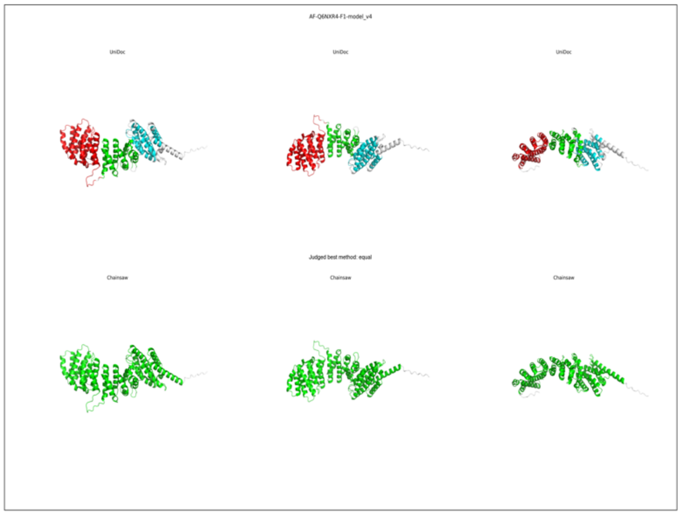

AF-Q6NXR4-F1-model_v4
UniDoc
UniDoc
UniDoc
Judged best method: equal
Chainsaw
Chainsaw
Chainsaw

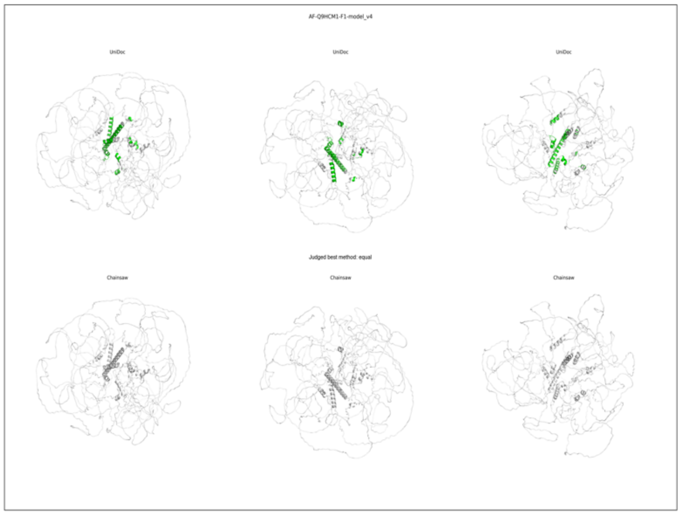

AF-Q9HCM1-F1-model_v4
UniDoc
UniDoc
UniDoc
Judged best method: equal
Chainsaw
Chainsaw
Chainsaw

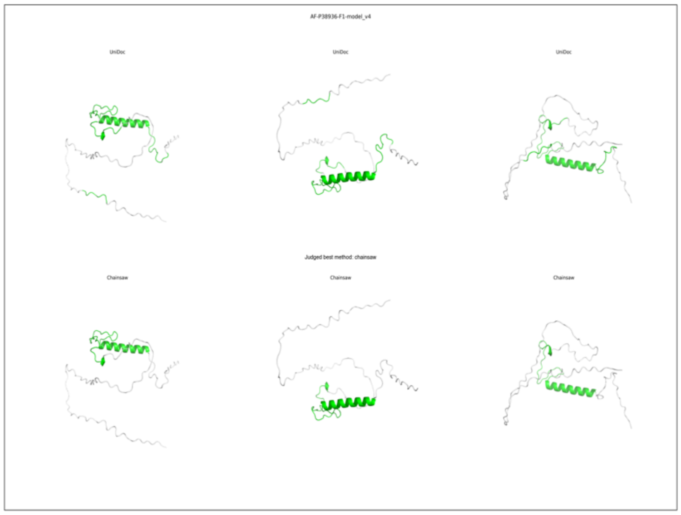

AF-P38936-F1-model_v4
UniDoc
UniDoc
UniDoc
Judged best method: chainsaw
Chainsaw
Chainsaw
Chainsaw

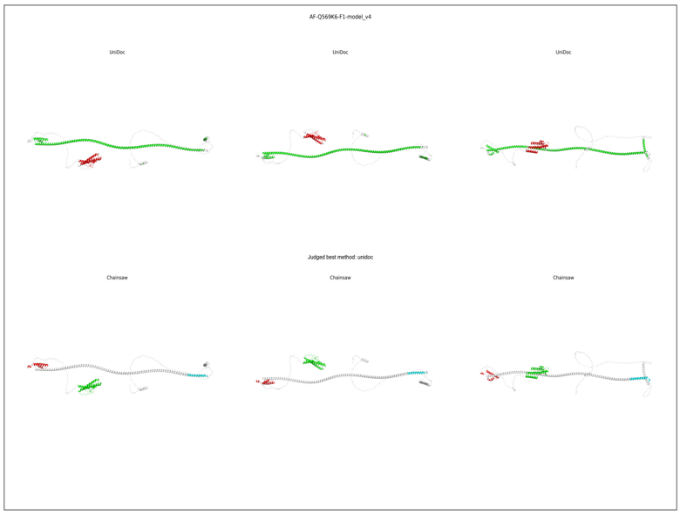

AF-Q569K6-F1-model_v4
UniDoc
UniDoc
UniDoc
Judged best method: unidoc
Chainsaw
Chainsaw
Chainsaw

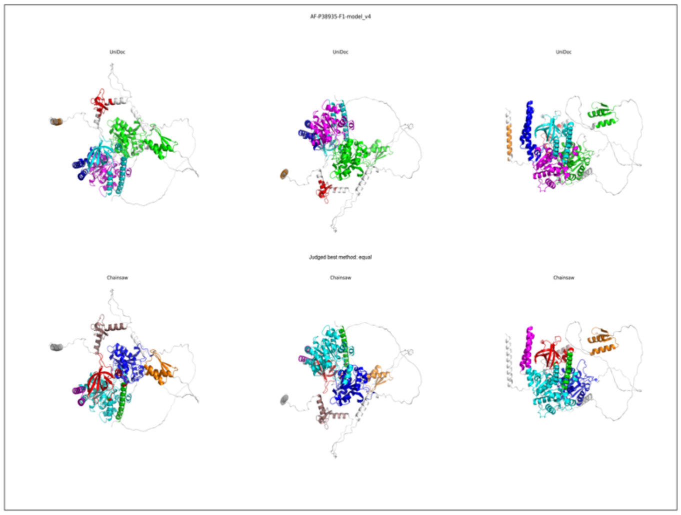

AF-P38935-F1-model_v4
UniDoc
UniDoc
UniDoc
Judged best method: equal
Chainsaw
Chainsaw
Chainsaw

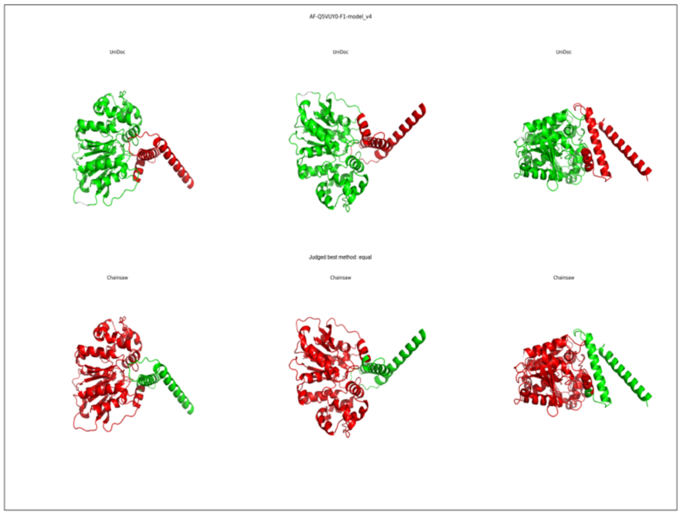

AF-Q5VUY0-F1-model_v4
UniDoc
UniDoc
UniDoc
Judged best method: equal
Chainsaw
Chainsaw
Chainsaw

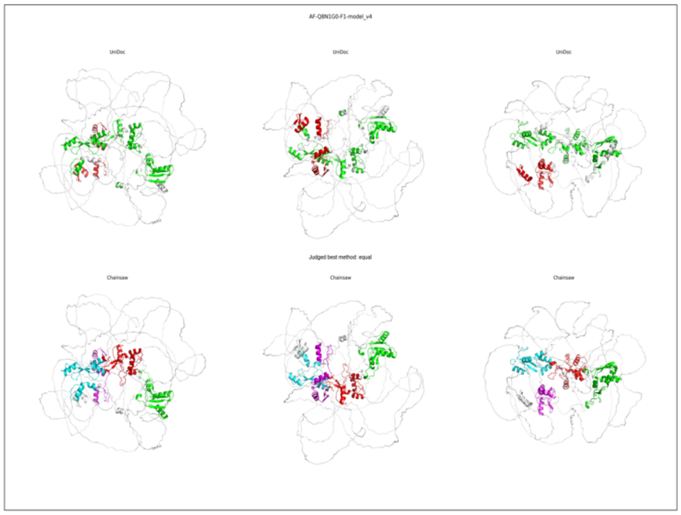
AF-Q8N1G0-F1-model_v4
UniDoc
UniDoc
UniDoc
Judged best method: equal
Chainsaw
Chainsaw
Chainsaw

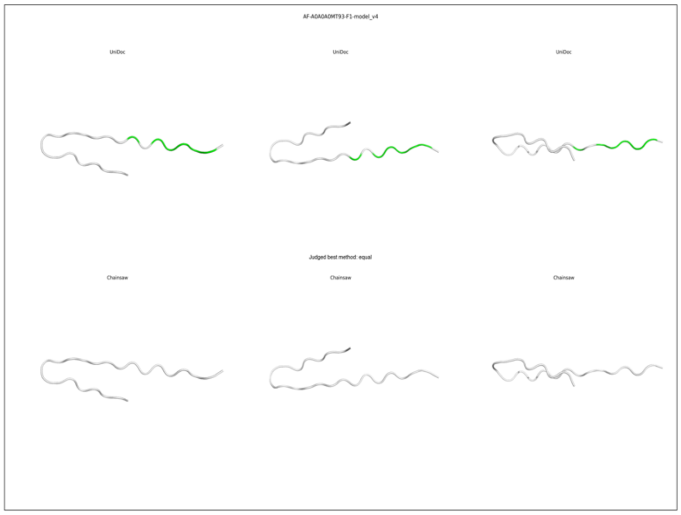
AF-A0A0A0MT93-F1-model_v4
UniDoc
UniDoc
UniDoc
Judged best method: equal
Chainsaw
Chainsaw
Chainsaw

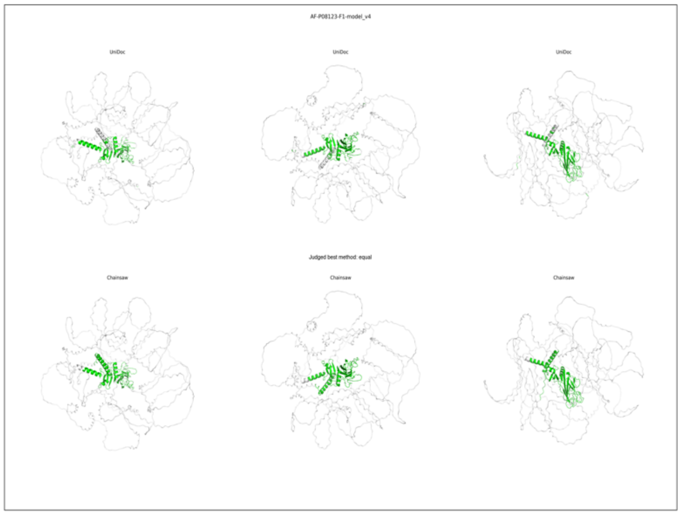
AF-P08123-F1-model_v4
UniDoc
UniDoc
UniDoc
Judged best method: equal
Chainsaw
Chainsaw
Chainsaw

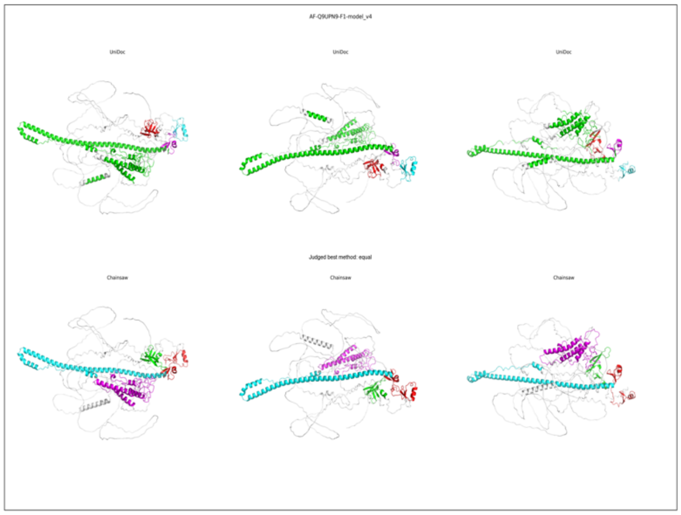
AF-Q9UPN9-F1-model_v4
UniDoc
UniDoc
UniDoc
Judged best method: equal
Chainsaw
Chainsaw
Chainsaw

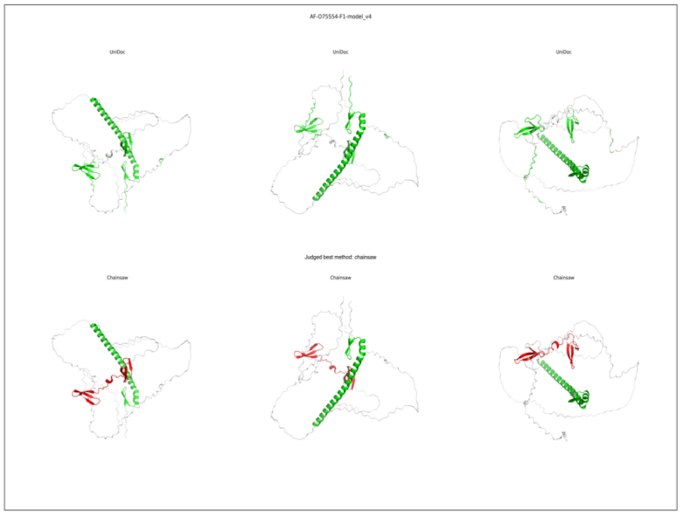
AF-O75554-F1-model_v4
UniDoc
UniDoc
UniDoc
Judged best method: chainsaw
Chainsaw
Chainsaw
Chainsaw

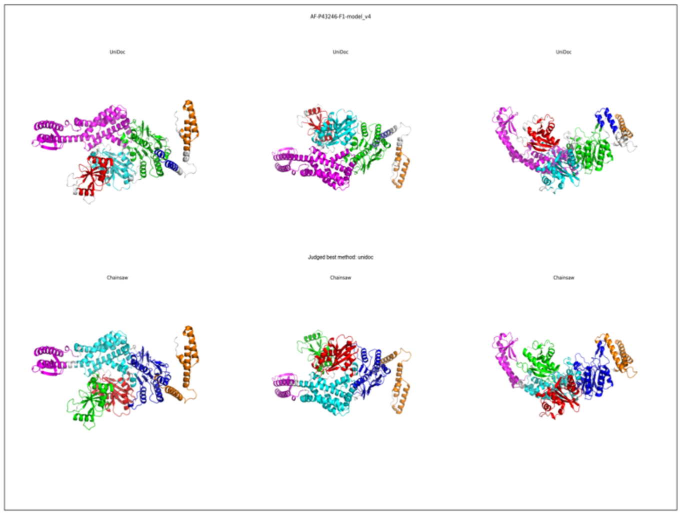
AF-P43246-F1-model_v4
UniDoc
UniDoc
UniDoc
Judged best method: unidoc
Chainsaw
Chainsaw
Chainsaw

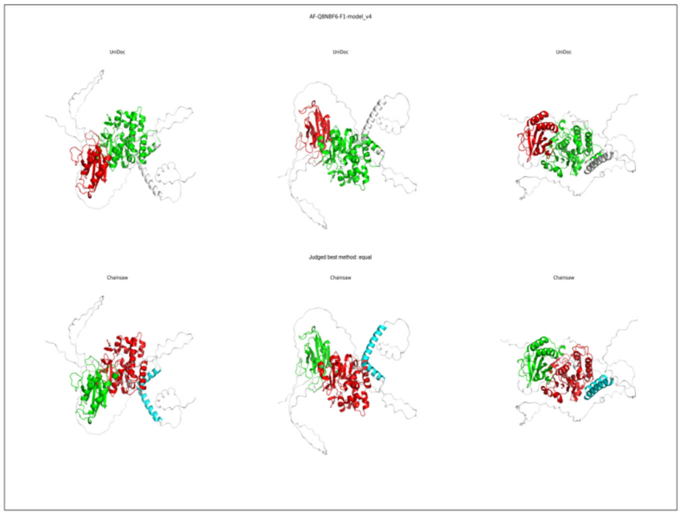
AF-Q8NBF6-F1-model_v4
UniDoc
UniDoc
UniDoc
Judged best method: equal
Chainsaw
Chainsaw
Chainsaw

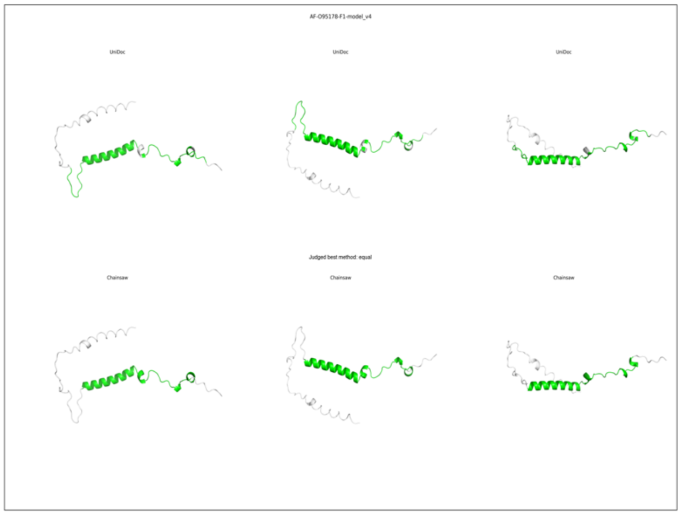
AF-O95178-F1-model_v4
UniDoc
UniDoc
UniDoc
Judged best method: equal
Chainsaw
Chainsaw
Chainsaw

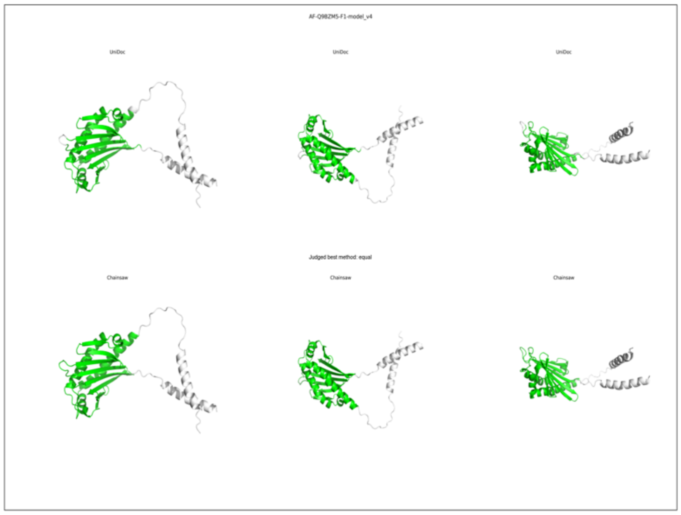
AF-Q9BZM5-F1-model_v4
UniDoc
UniDoc
UniDoc
Judged best method: equal
Chainsaw
Chainsaw
Chainsaw

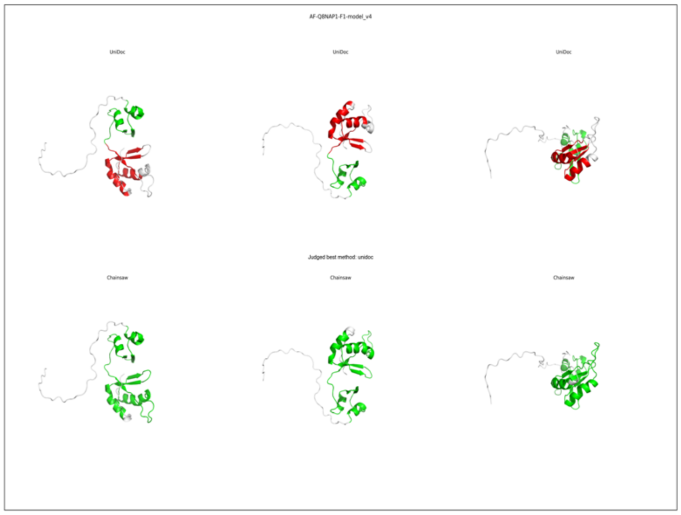
AF-Q8NAP1-F1-model_v4
UniDoc
UniDoc
UniDoc
Judged best method: unidoc
Chainsaw
Chainsaw
Chainsaw

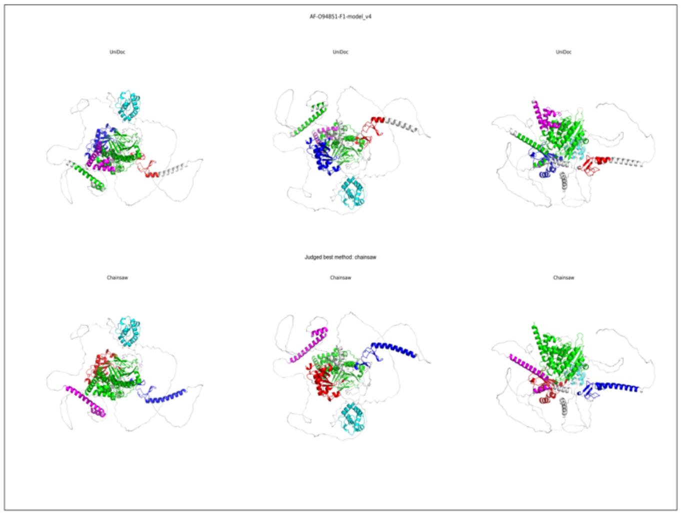
AF-O94851-F1-model_v4
UniDoc
UniDoc
UniDoc
Judged best method: chainsaw
Chainsaw
Chainsaw
Chainsaw

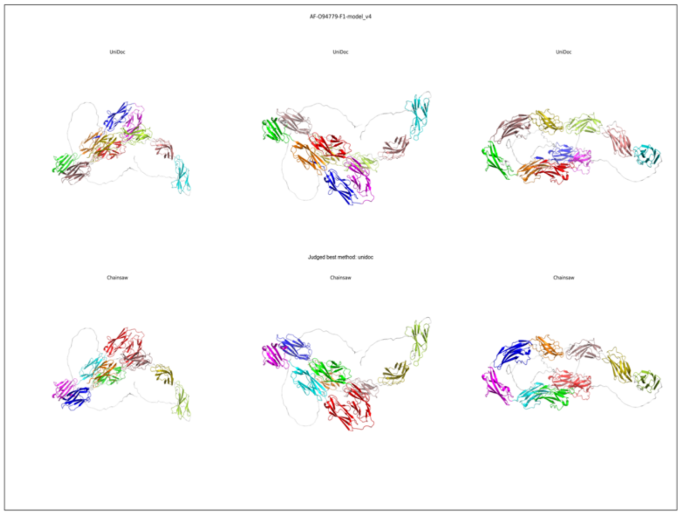
AF-O94779-F1-model_v4
UniDoc
UniDoc
UniDoc
Judged best method: unidoc
Chainsaw
Chainsaw
Chainsaw

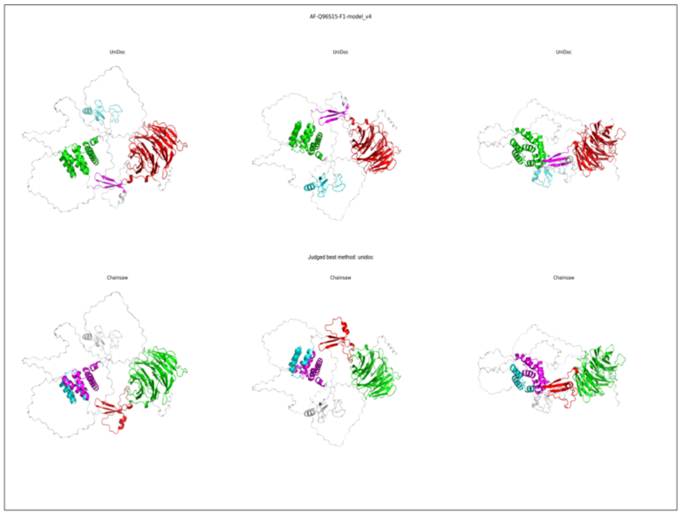
AF-Q96515-F1-model_v4
UniDoc
UniDoc
UniDoc
Judged best method: unidoc
Chainsaw
Chainsaw
Chainsaw

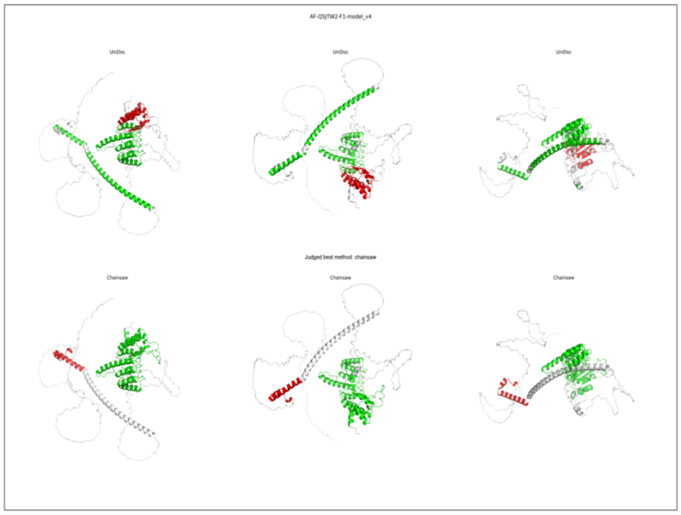
AF-Q5JTW2-F1-model_v4
UniDoc
UniDoc
UniDoc
Judged best method: chainsaw
Chainsaw
Chainsaw
Chainsaw

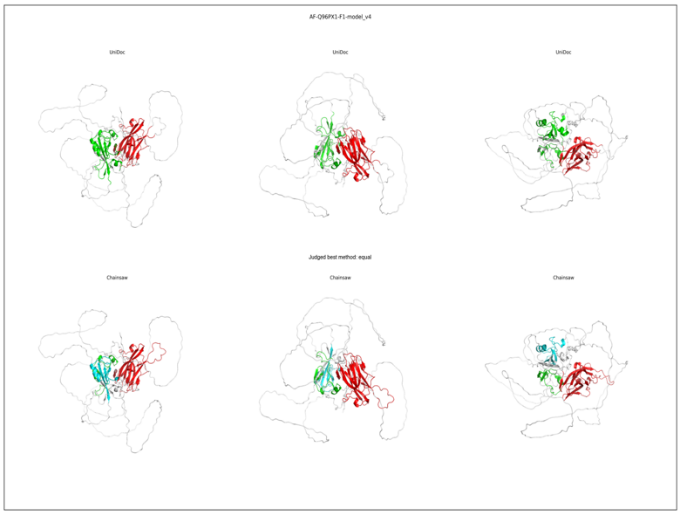
AF-Q96PX1-F1-model_v4
UniDoc
UniDoc
UniDoc
Judged best method: equal
Chainsaw
Chainsaw
Chainsaw

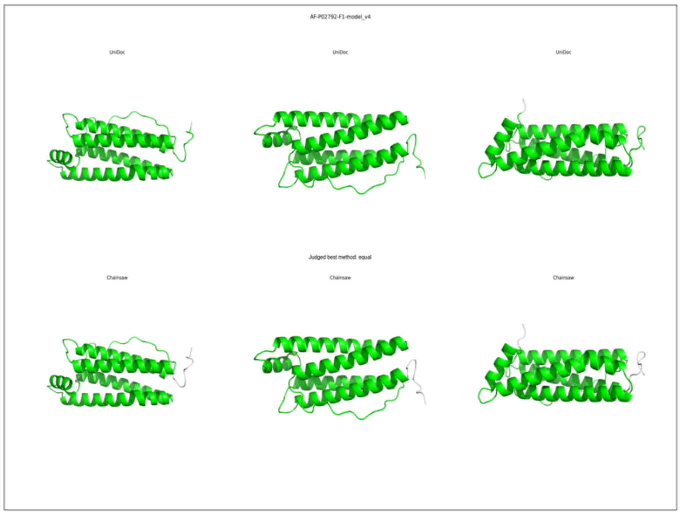
AF-P02792-F1-model_v4
UniDoc
UniDoc
UniDoc
Judged best method: equal
Chainsaw
Chainsaw
Chainsaw

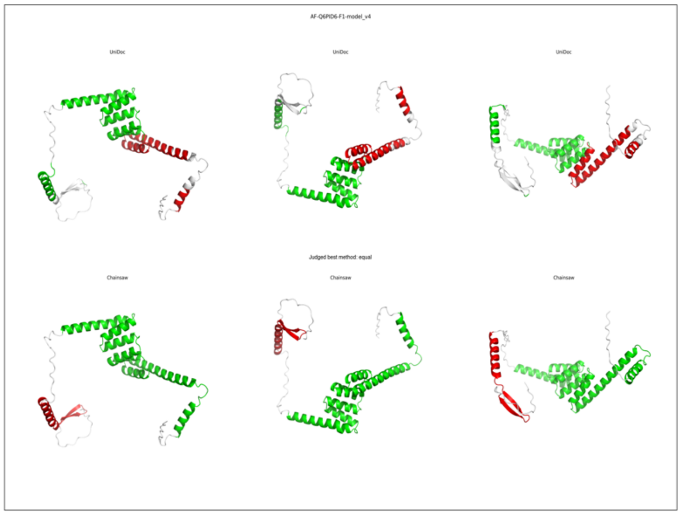
AF-Q6PID6-F1-model_v4
UniDoc
UniDoc
UniDoc
Judged best method: equal
Chainsaw
Chainsaw
Chainsaw

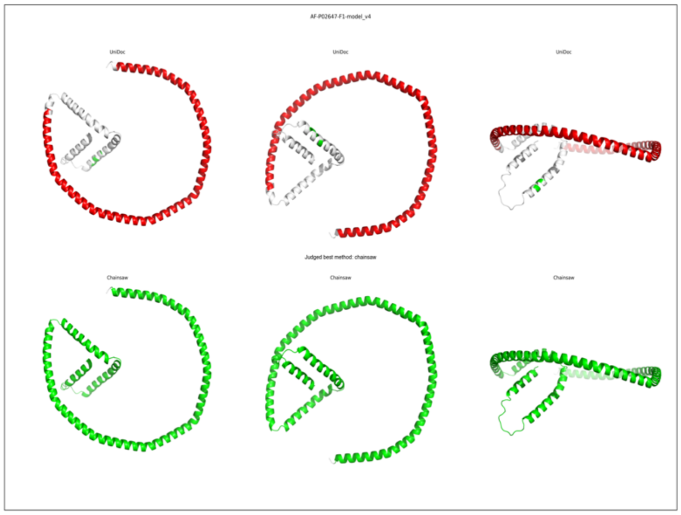
AF-P02647-F1-model_v4
UniDoc
UniDoc
UniDoc
Judged best method: chainsaw
Chainsaw
Chainsaw
Chainsaw

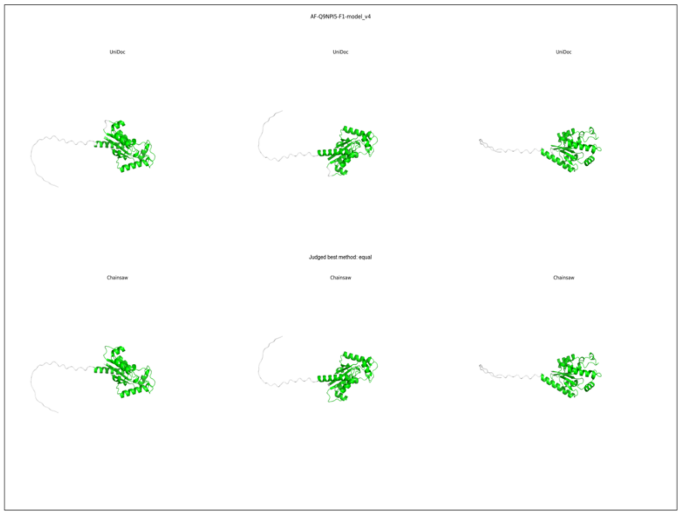
AF-Q9NPI5-F1-model_v4
UniDoc
UniDoc
UniDoc
Judged best method: equal
Chainsaw
Chainsaw
Chainsaw

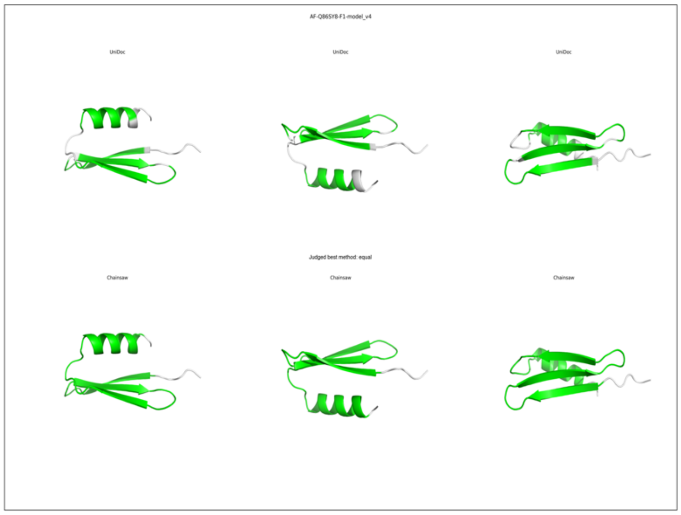
AF-Q86SY8-F1-model_v4
UniDoc
UniDoc
UniDoc
Judged best method: equal
Chainsaw
Chainsaw
Chainsaw

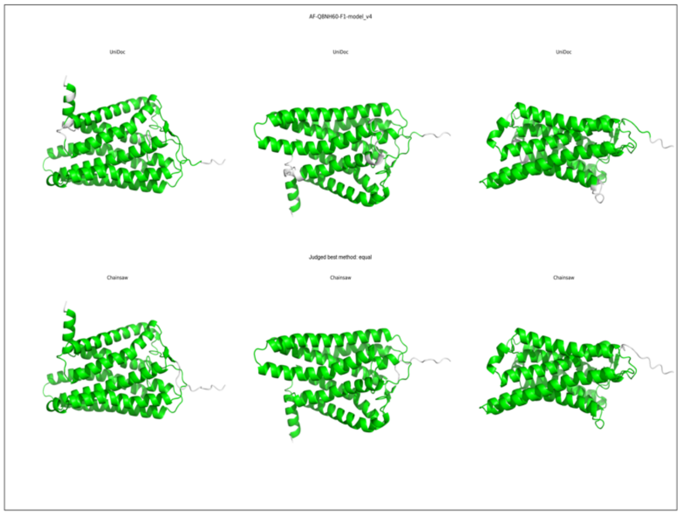
AF-Q8NH60-F1-model_v4
UniDoc
UniDoc
UniDoc
Judged best method: equal
Chainsaw
Chainsaw
Chainsaw

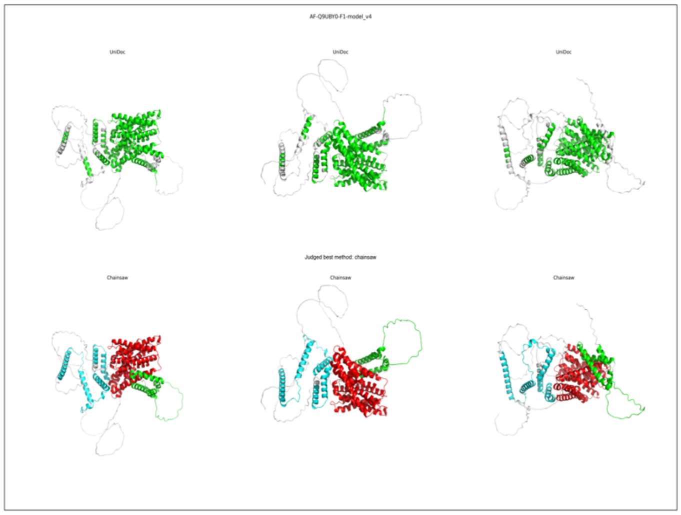
AF-Q9UBY0-F1-model_v4
UniDoc
UniDoc
UniDoc
Judged best method: chainsaw
Chainsaw
Chainsaw
Chainsaw

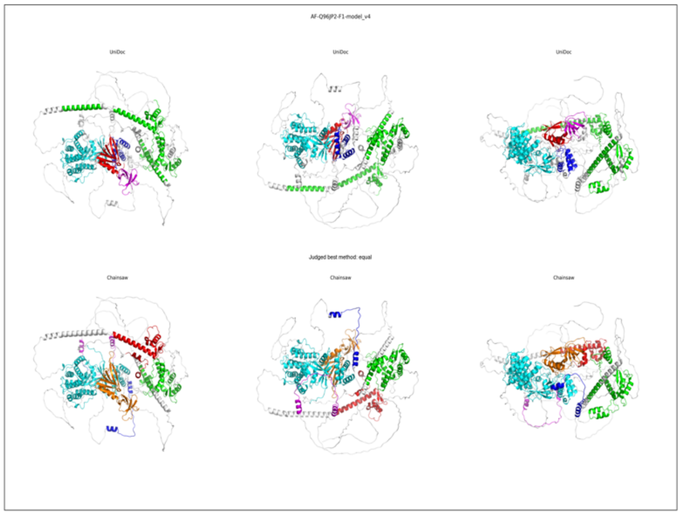
AF-Q96JP2-F1-model_v4
UniDoc
UniDoc
UniDoc
Judged best method: equal
Chainsaw
Chainsaw
Chainsaw

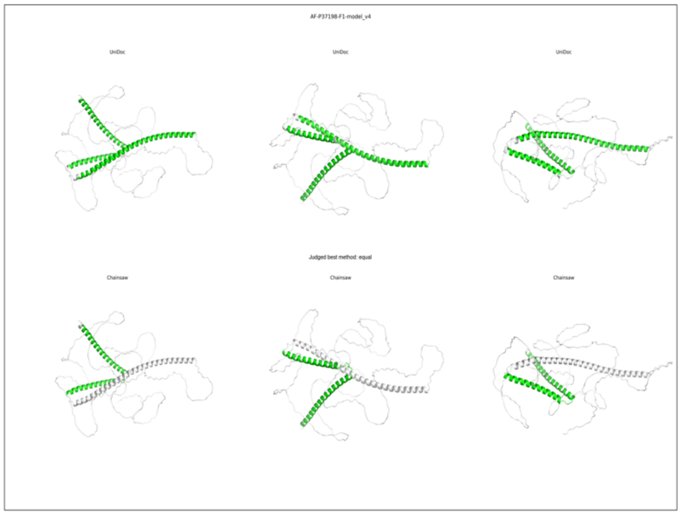
AF-P37198-F1-model_v4
UniDoc
UniDoc
UniDoc
Judged best method: equal
Chainsaw
Chainsaw
Chainsaw

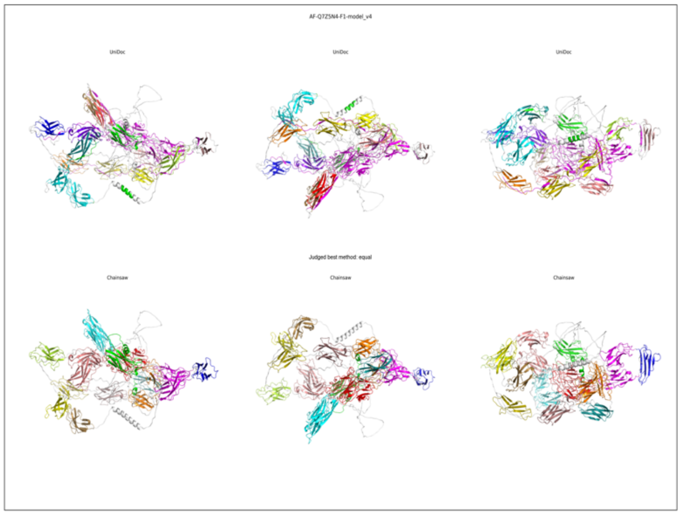
AF-Q7Z5N4-F1-model_v4
UniDoc
UniDoc
UniDoc
Judged best method: equal
Chainsaw
Chainsaw
Chainsaw

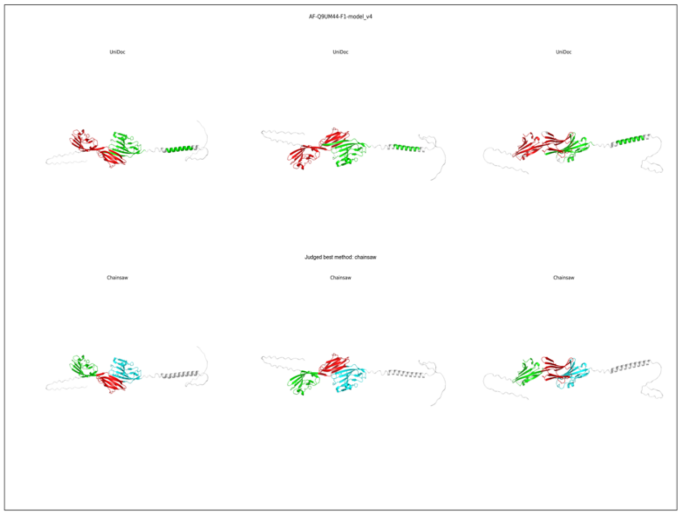
AF-Q9UM44-F1-model_v4
UniDoc
UniDoc
UniDoc
Judged best method: chainsaw
Chainsaw
Chainsaw
Chainsaw

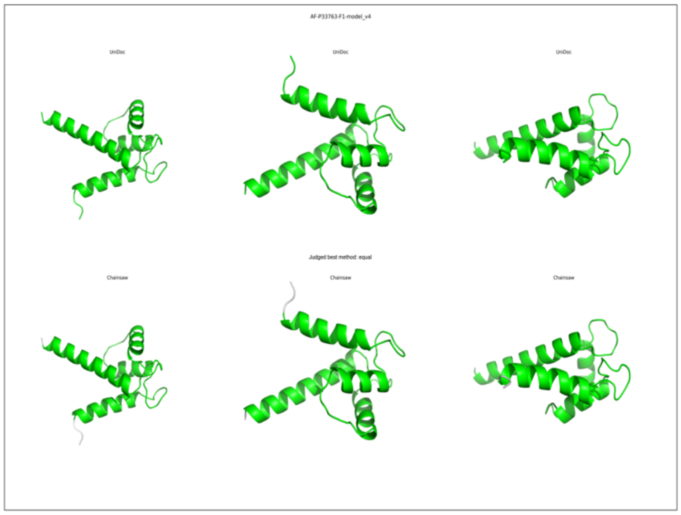
AF-P33763-F1-model_v4
UniDoc
UniDoc
UniDoc
Judged best method: equal
Chainsaw
Chainsaw
Chainsaw

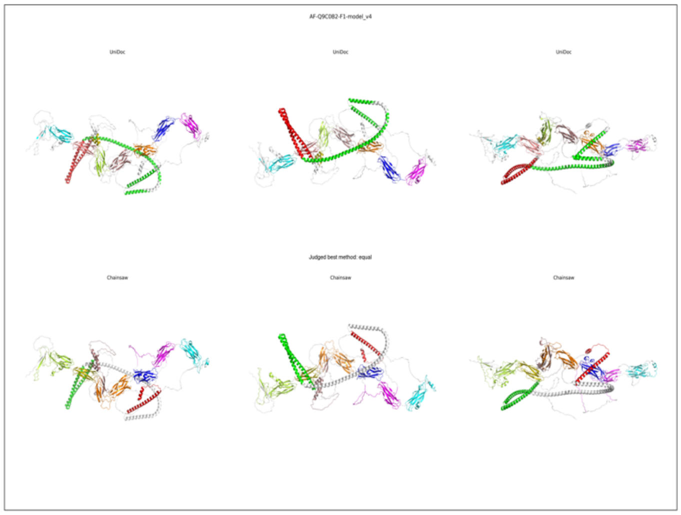
AF-Q9C0B2-F1-model_v4
UniDoc
UniDoc
UniDoc
Judged best method: equal
Chainsaw
Chainsaw
Chainsaw

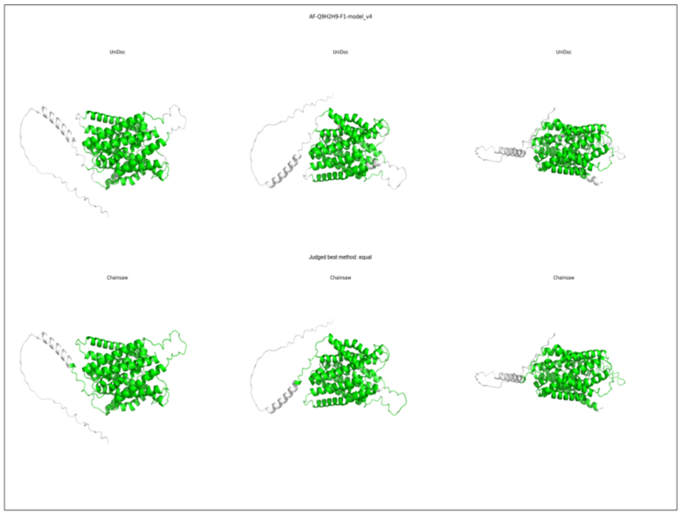
AF-Q9H2H9-F1-model_v4
UniDoc
UniDoc
UniDoc
Judged best method: equal
Chainsaw
Chainsaw
Chainsaw

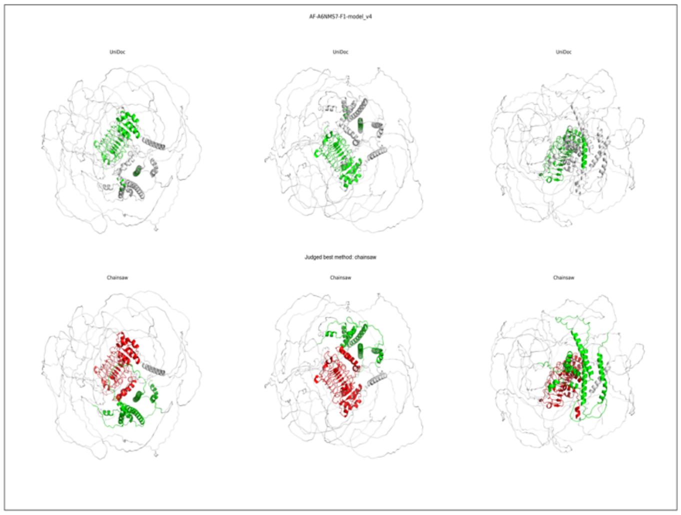
AF-A6NMS7-F1-model_v4
UniDoc
UniDoc
UniDoc
Judged best method: chainsaw
Chainsaw
Chainsaw
Chainsaw

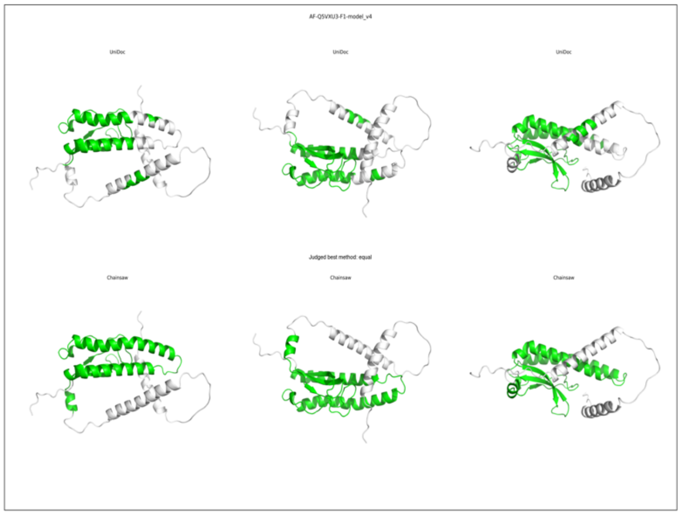
AF-Q5VXU3-F1-model_v4
UniDoc
UniDoc
UniDoc
Judged best method: equal
Chainsaw
Chainsaw
Chainsaw

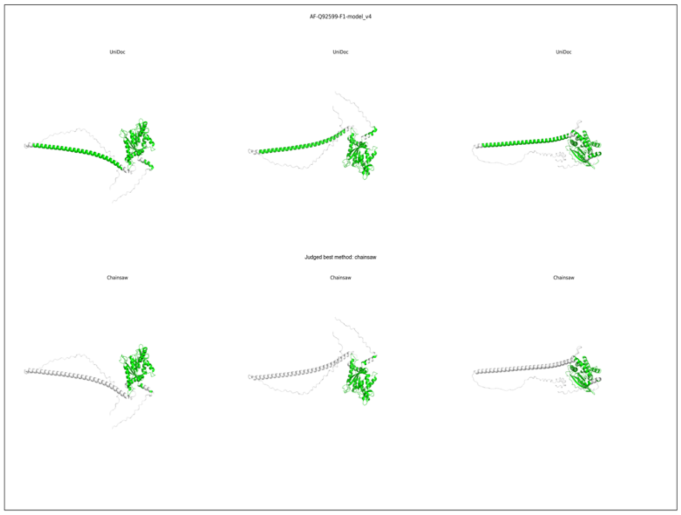
AF-Q92599-F1-model_v4
UniDoc
UniDoc
UniDoc
Judged best method: chainsaw
Chainsaw
Chainsaw
Chainsaw

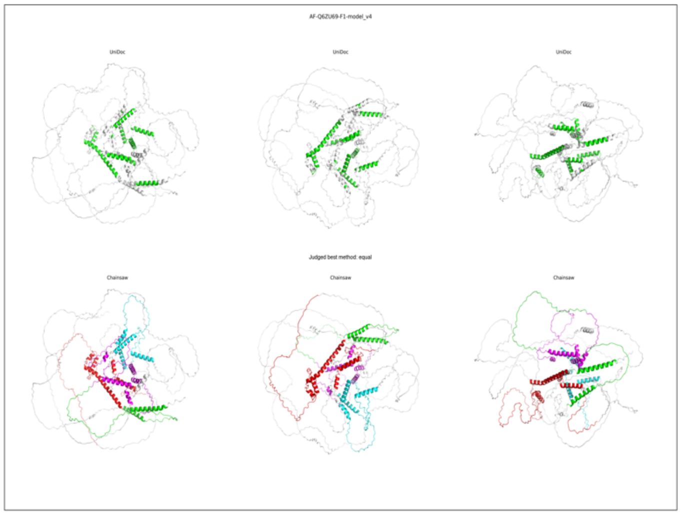
AF-Q6ZU69-F1-model_v4
UniDoc
UniDoc
UniDoc
Judged best method: equal
Chainsaw
Chainsaw
Chainsaw

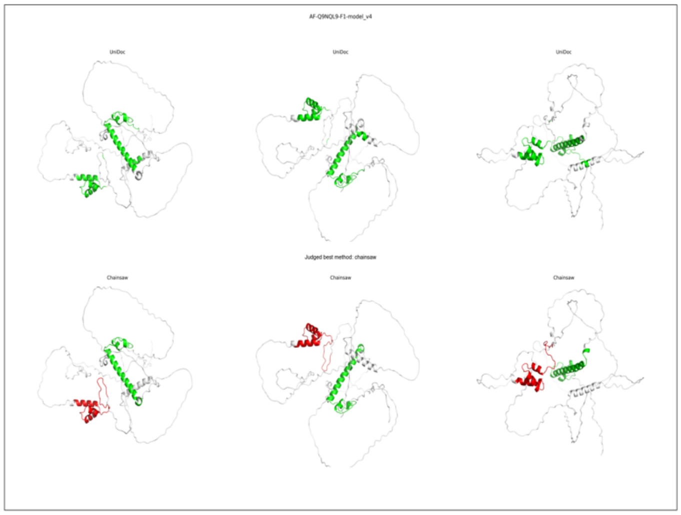
AF-Q9NQL9-F1-model_v4
UniDoc
UniDoc
UniDoc
Judged best method: chainsaw
Chainsaw
Chainsaw
Chainsaw

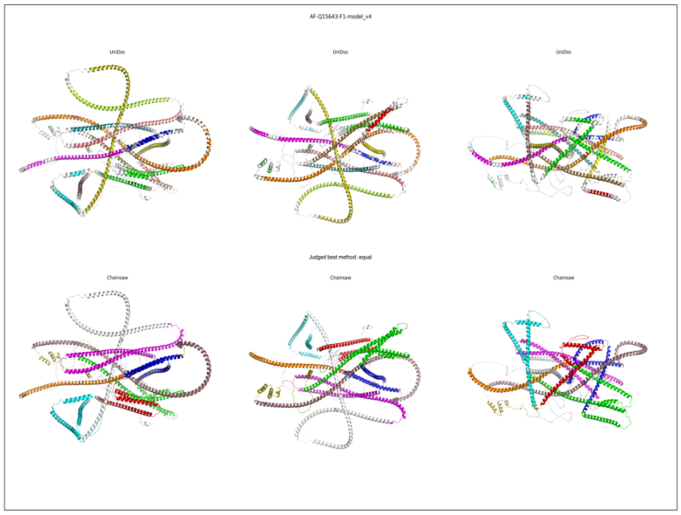
AF-Q15643-F1-model_v4
UniDoc
UniDoc
UniDoc
Judged best method: equal
Chainsaw
Chainsaw
Chainsaw

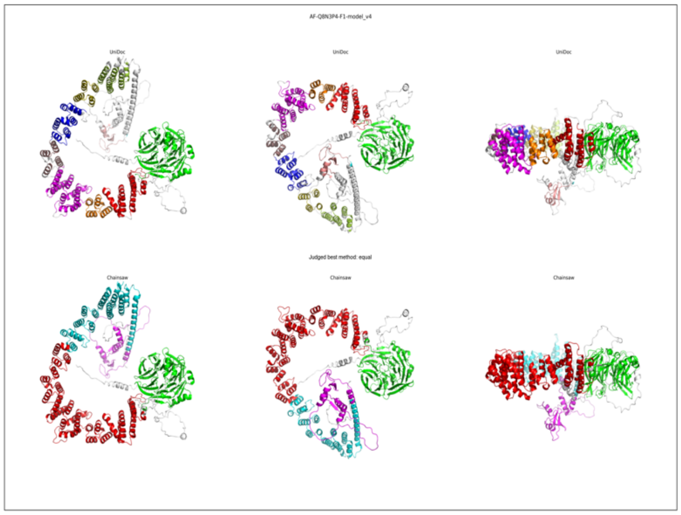
AF-Q8N3P4-F1-model_v4
UniDoc
UniDoc
UniDoc
Judged best method: equal
Chainsaw
Chainsaw
Chainsaw

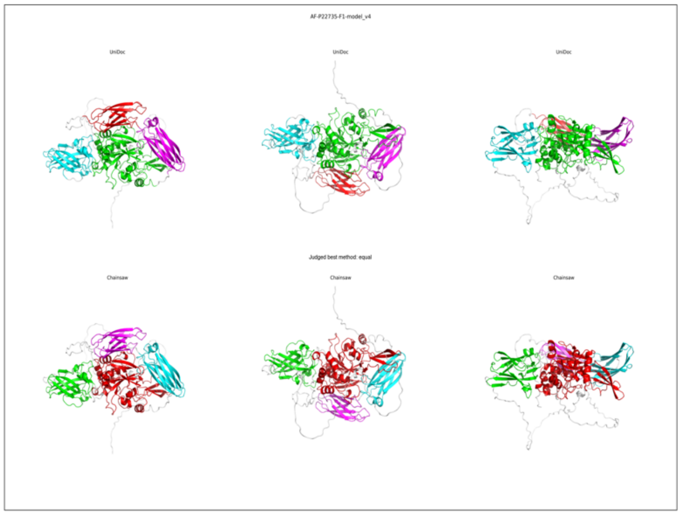
AF-P22735-F1-model_v4
UniDoc
UniDoc
UniDoc
Judged best method: equal
Chainsaw
Chainsaw
Chainsaw

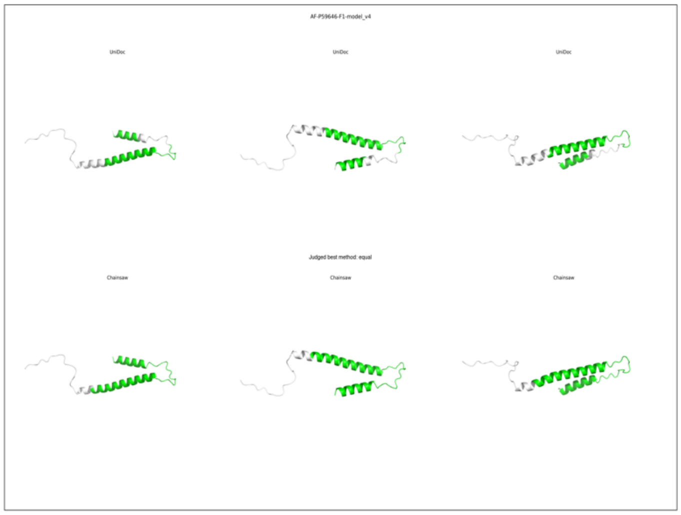
AF-P59646-F1-model_v4
UniDoc
UniDoc
UniDoc
Judged best method: equal
Chainsaw
Chainsaw
Chainsaw

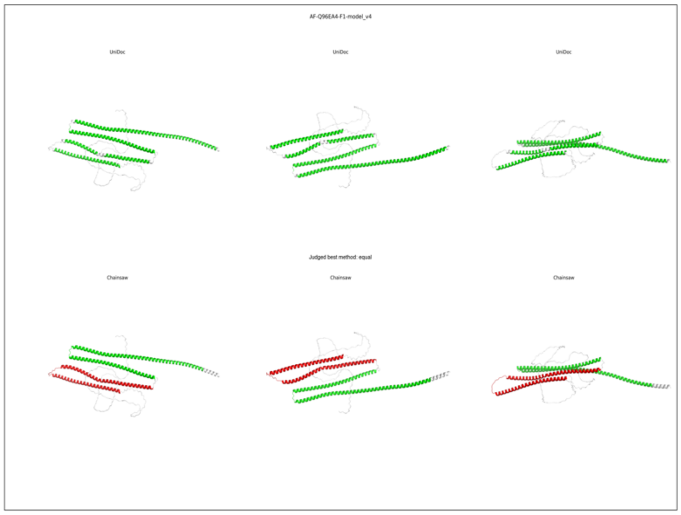
AF-Q96EA4-F1-model_v4
UniDoc
UniDoc
UniDoc
Judged best method: equal
Chainsaw
Chainsaw
Chainsaw

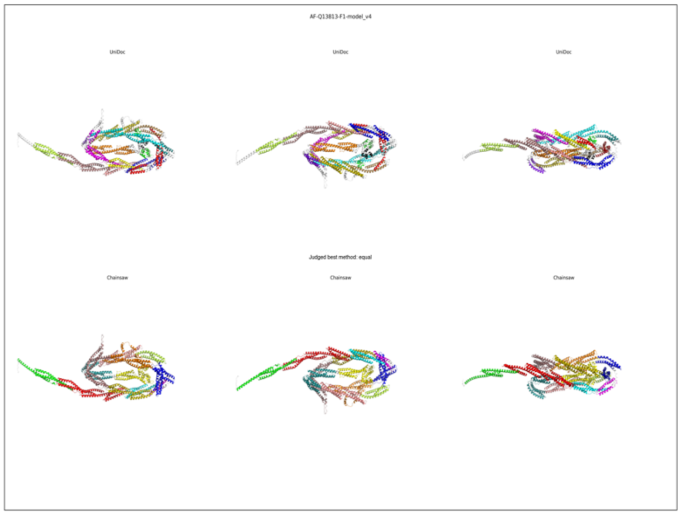
AF-Q13813-F1-model_v4
UniDoc
UniDoc
UniDoc
Judged best method: equal
Chainsaw
Chainsaw
Chainsaw

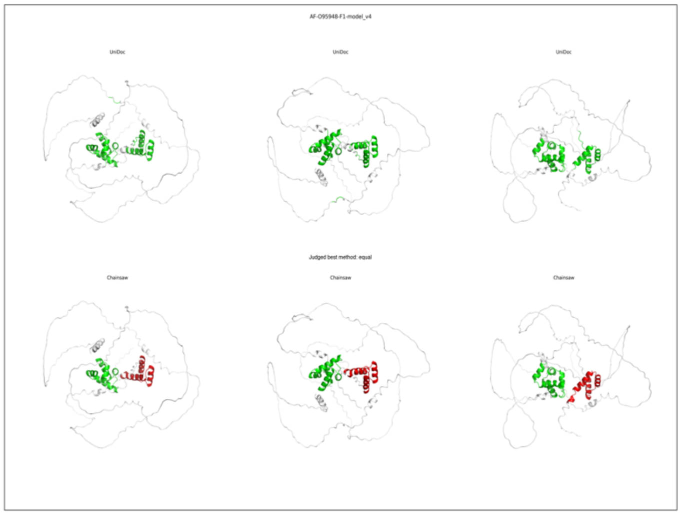
AF-O95948-F1-model_v4
UniDoc
UniDoc
UniDoc
Judged best method: equal
Chainsaw
Chainsaw
Chainsaw

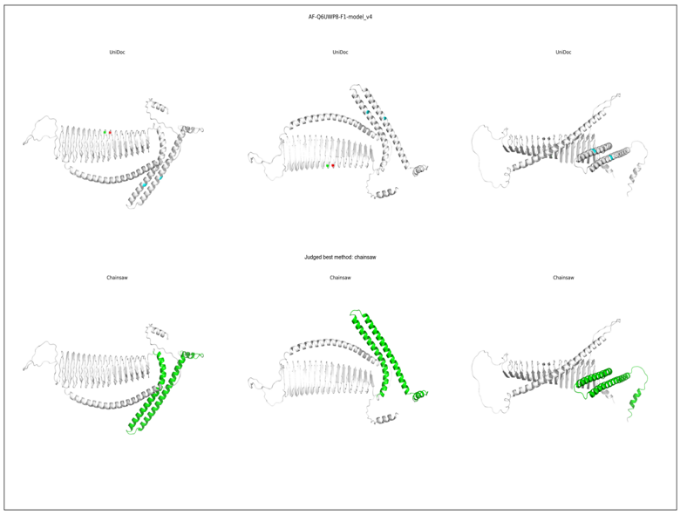
AF-Q6UWP8-F1-model_v4
UniDoc
UniDoc
UniDoc
Judged best method: chainsaw
Chainsaw
Chainsaw
Chainsaw

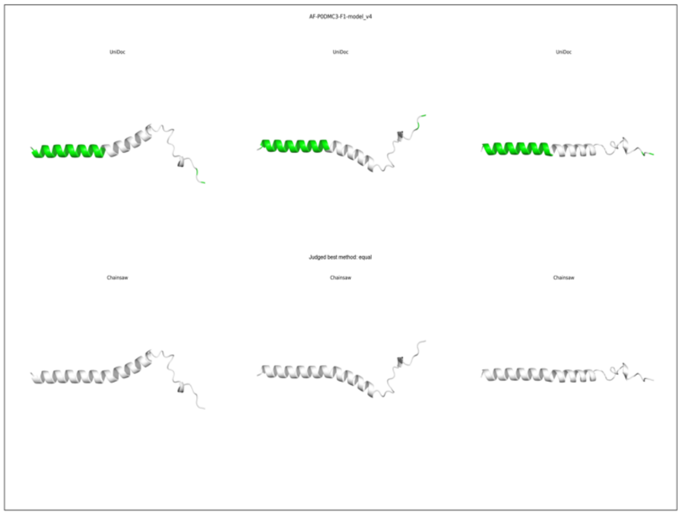
AF-P0DMC3-F1-model_v4
UniDoc
UniDoc
UniDoc
Judged best method: equal
Chainsaw
Chainsaw
Chainsaw

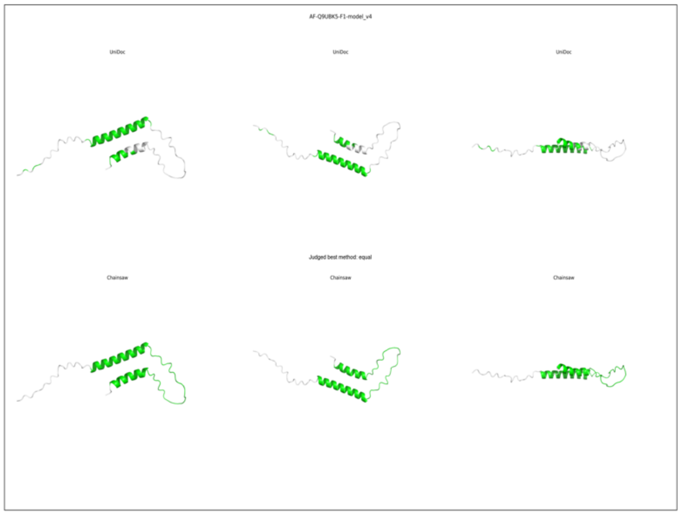
AF-Q9UBK5-F1-model_v4
UniDoc
UniDoc
UniDoc
Judged best method: equal
Chainsaw
Chainsaw
Chainsaw

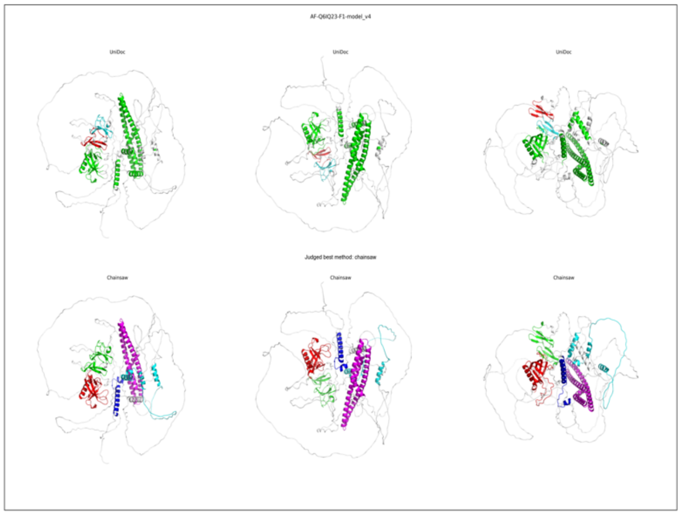
AF-Q6IQ23-F1-model_v4
UniDoc
UniDoc
UniDoc
Judged best method: chainsaw
Chainsaw
Chainsaw
Chainsaw

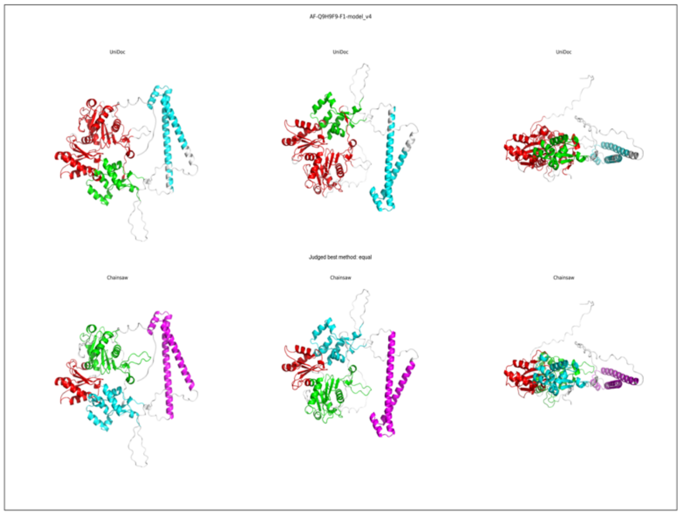
AF-Q9H9F9-F1-model_v4
UniDoc
UniDoc
UniDoc
Judged best method: equal
Chainsaw
Chainsaw
Chainsaw

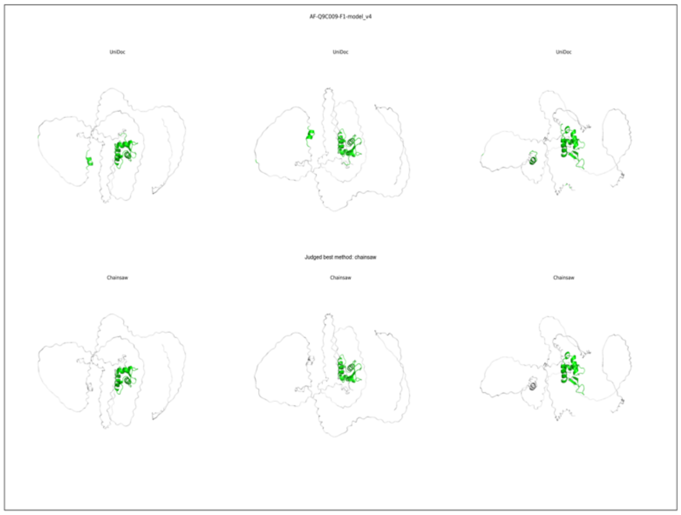
AF-Q9C009-F1-model_v4
UniDoc
UniDoc
UniDoc
Judged best method: chainsaw
Chainsaw
Chainsaw
Chainsaw

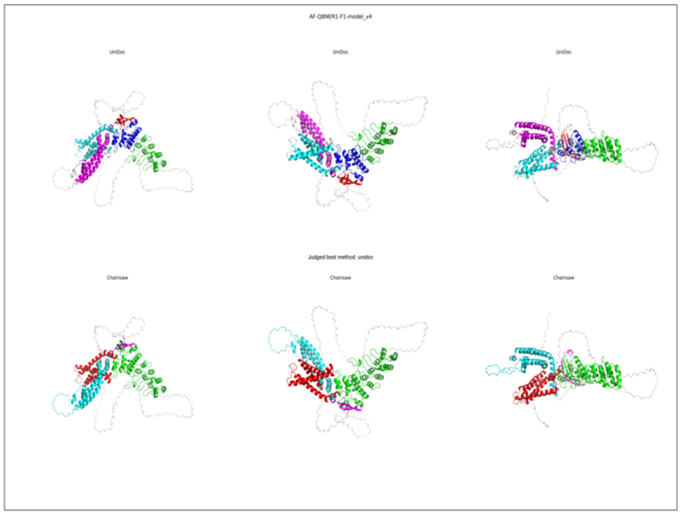
AF-Q8NER1-F1-model_v4
UniDoc
UniDoc
UniDoc
Judged best method: unidoc
Chainsaw
Chainsaw
Chainsaw

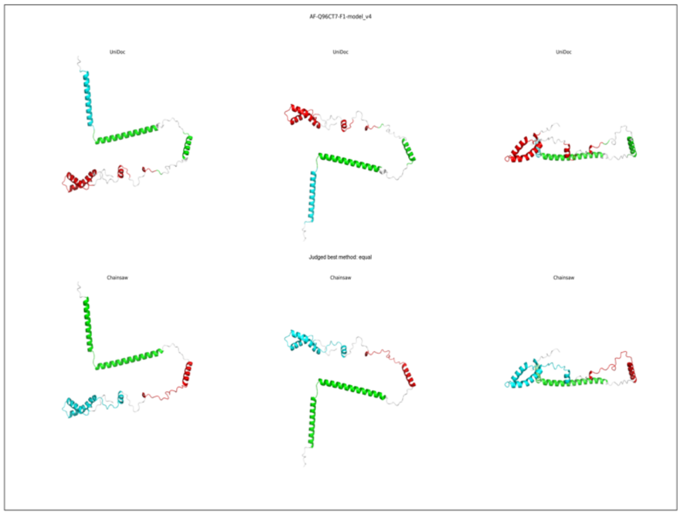
AF-Q96CT7-F1-model_v4
UniDoc
UniDoc
UniDoc
Judged best method: equal
Chainsaw
Chainsaw
Chainsaw

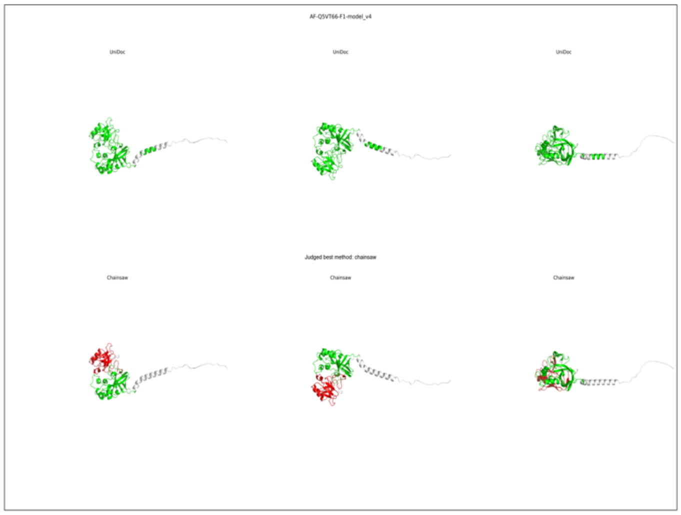
AF-Q5VT66-F1-model_v4
UniDoc
UniDoc
UniDoc
Judged best method: chainsaw
Chainsaw
Chainsaw
Chainsaw

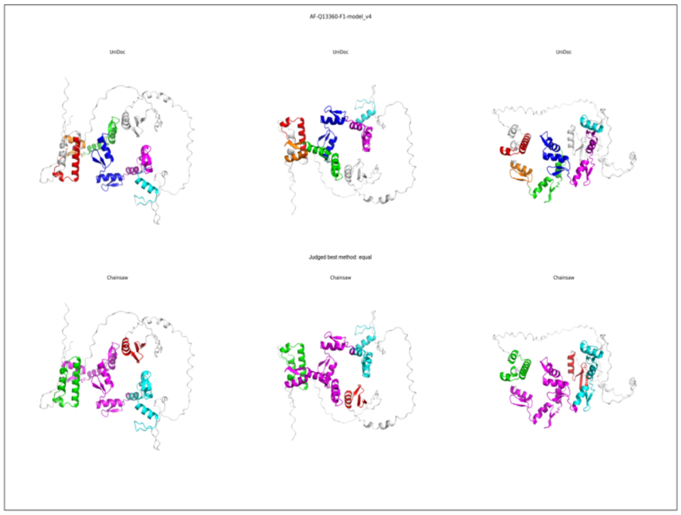
AF-Q13360-F1-model_v4
UniDoc
UniDoc
UniDoc
Judged best method: equal
Chainsaw
Chainsaw
Chainsaw

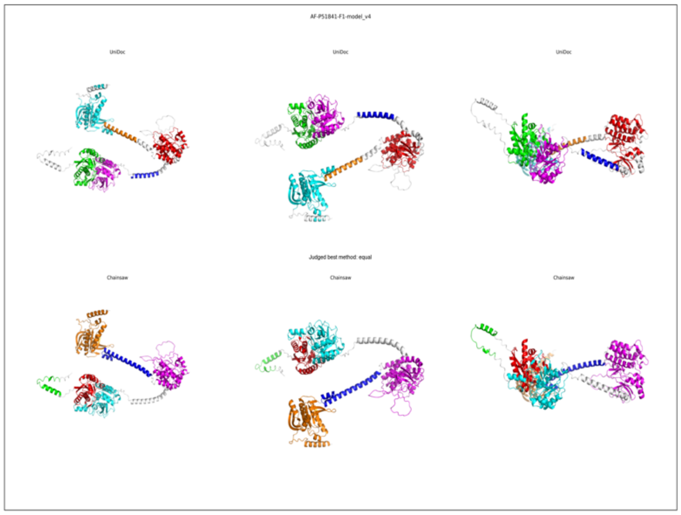
AF-P51841-F1-model_v4
UniDoc
UniDoc
UniDoc
Judged best method: equal
Chainsaw
Chainsaw
Chainsaw

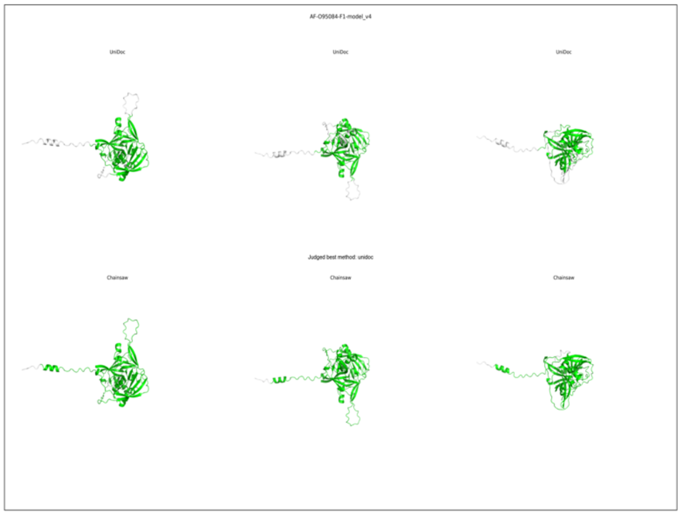
AF-O95084-F1-model_v4
UniDoc
UniDoc
UniDoc
Judged best method: unidoc
Chainsaw
Chainsaw
Chainsaw

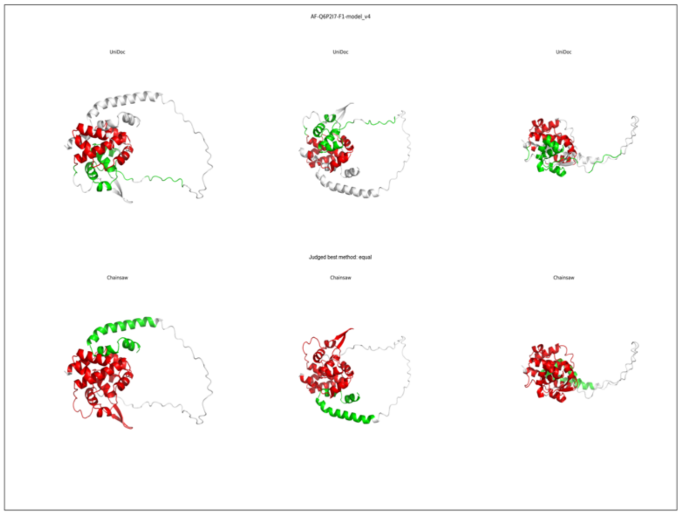
AF-Q6P2I7-F1-model_v4
UniDoc
UniDoc
UniDoc
Judged best method: equal
Chainsaw
Chainsaw
Chainsaw

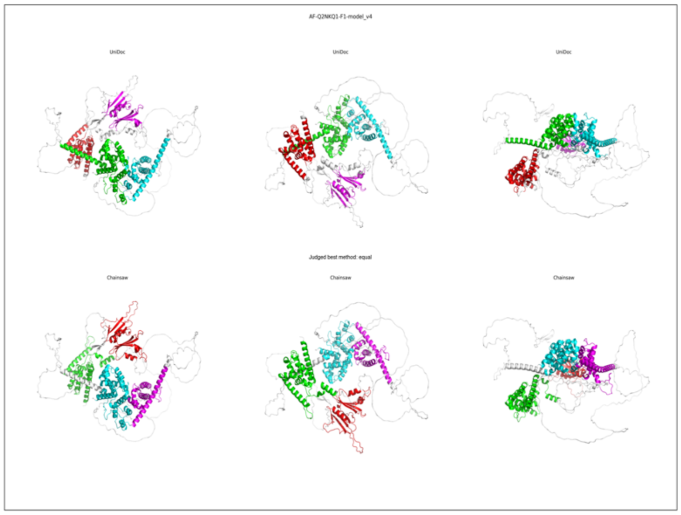
AF-Q2NKQ1-F1-model_v4
UniDoc
UniDoc
UniDoc
Judged best method: equal
Chainsaw
Chainsaw
Chainsaw

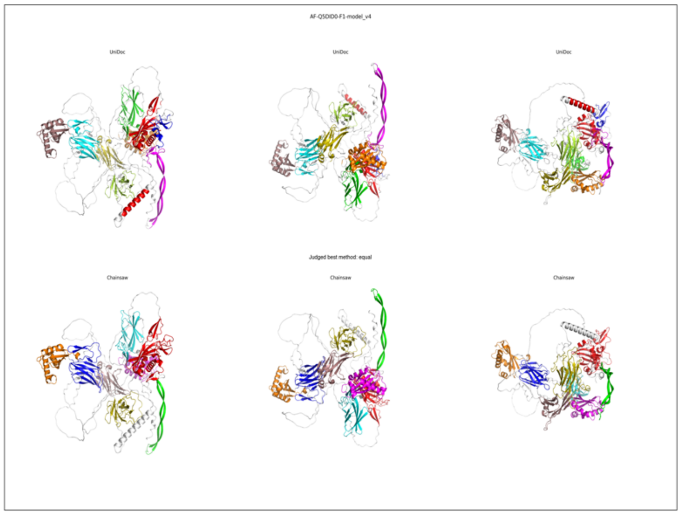
AF-Q5DID0-F1-model_v4
UniDoc
UniDoc
UniDoc
Judged best method: equal
Chainsaw
Chainsaw
Chainsaw

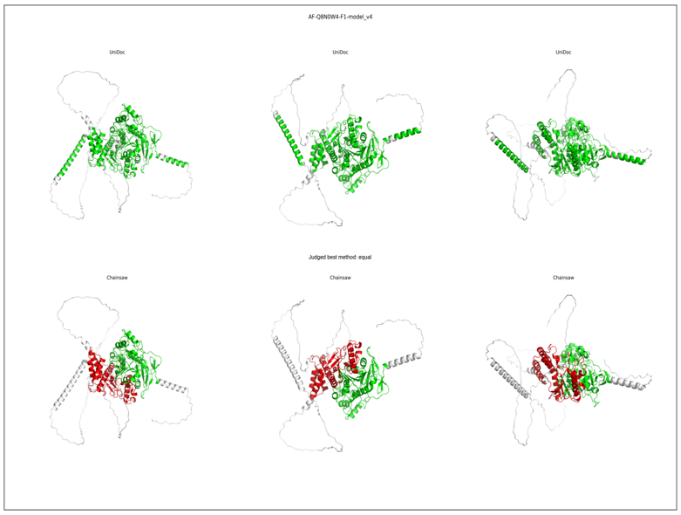
AF-Q8N0W4-F1-model_v4
UniDoc
UniDoc
UniDoc
Judged best method: equal
Chainsaw
Chainsaw
Chainsaw

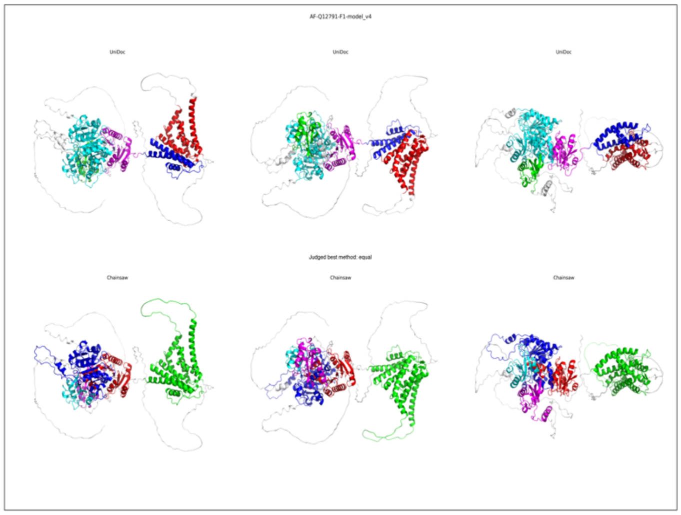
AF-Q12791-F1-model_v4
UniDoc
UniDoc
UniDoc
Judged best method: equal
Chainsaw
Chainsaw
Chainsaw

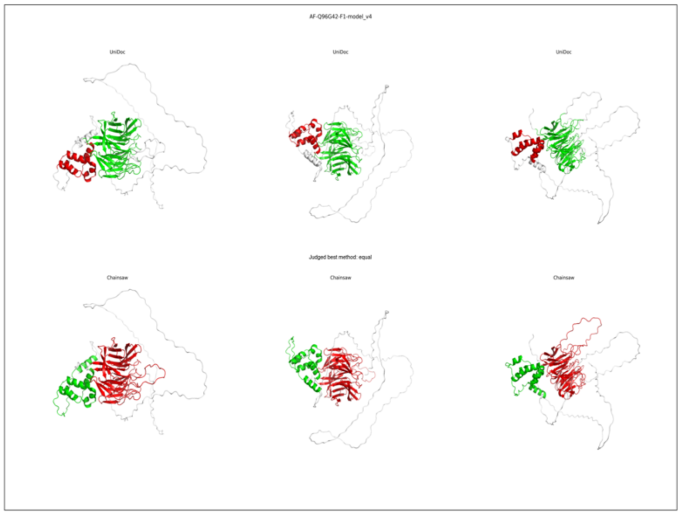
AF-Q96G42-F1-model_v4
UniDoc
UniDoc
UniDoc
Judged best method: equal
Chainsaw
Chainsaw
Chainsaw

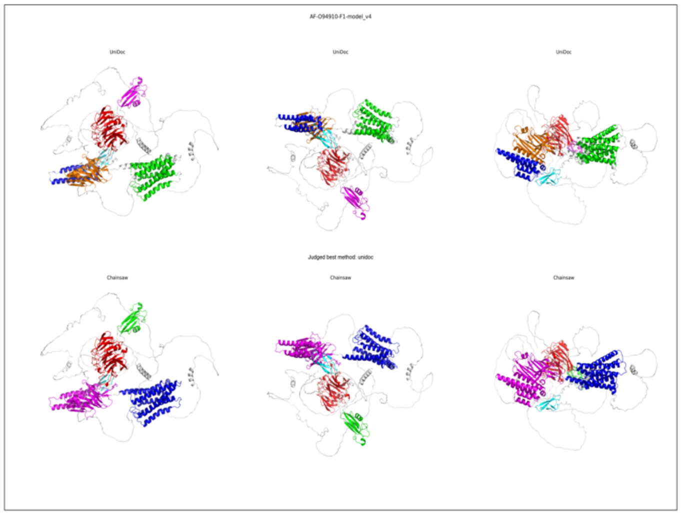
AF-O94910-F1-model_v4
UniDoc
UniDoc
UniDoc
Judged best method: unidoc
Chainsaw
Chainsaw
Chainsaw

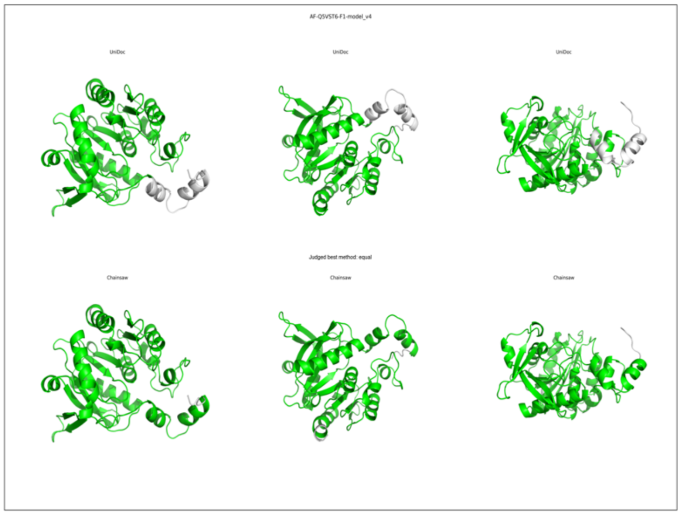

AF-Q5VST6-F1-model_v4
UniDoc
UniDoc
UniDoc
Judged best method: equal
Chainsaw
Chainsaw
Chainsaw

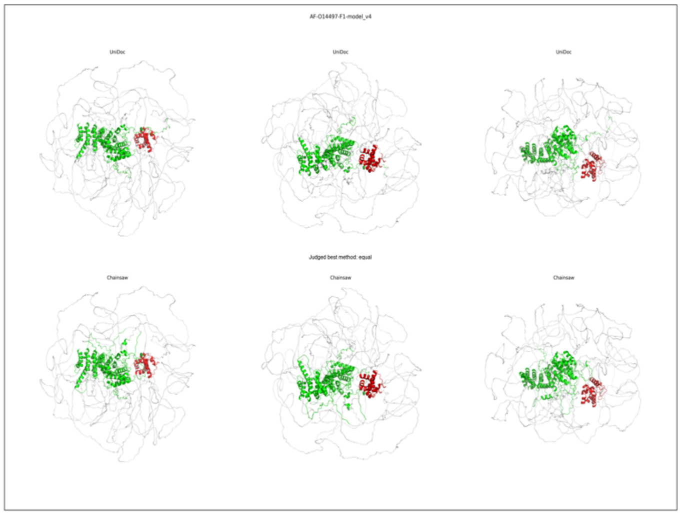

AF-O14497-F1-model_v4
UniDoc
UniDoc
UniDoc
Judged best method: equal
Chainsaw
Chainsaw
Chainsaw

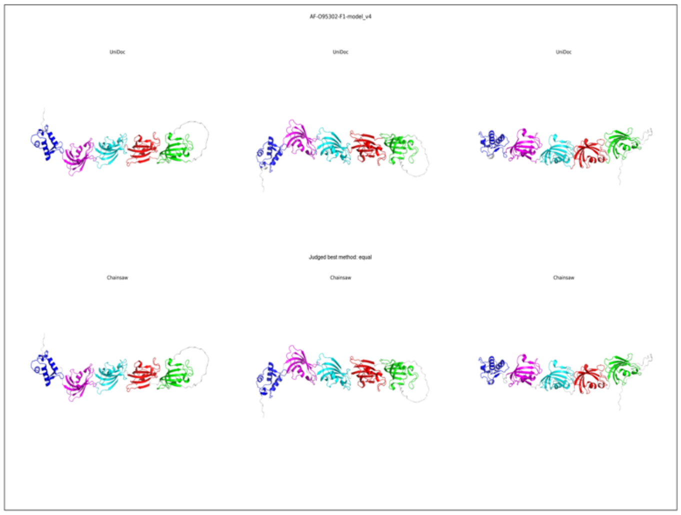

AF-O95302-F1-model_v4
UniDoc
UniDoc
UniDoc
Judged best method: equal
Chainsaw
Chainsaw
Chainsaw

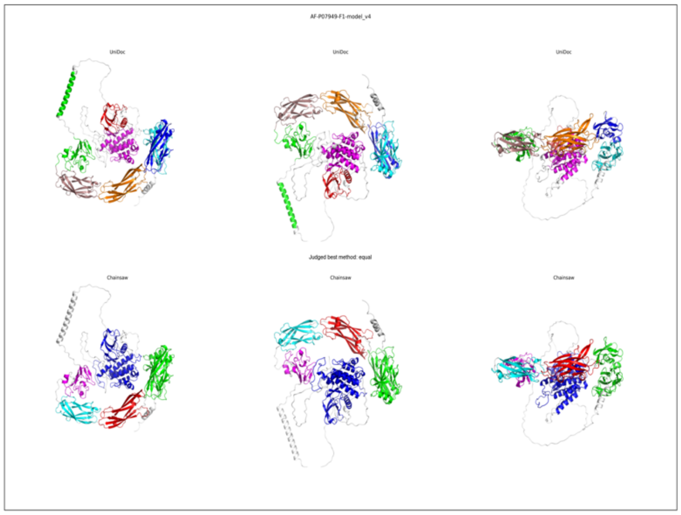

AF-P07949-F1-model_v4
UniDoc
UniDoc
UniDoc
Judged best method: equal
Chainsaw
Chainsaw
Chainsaw

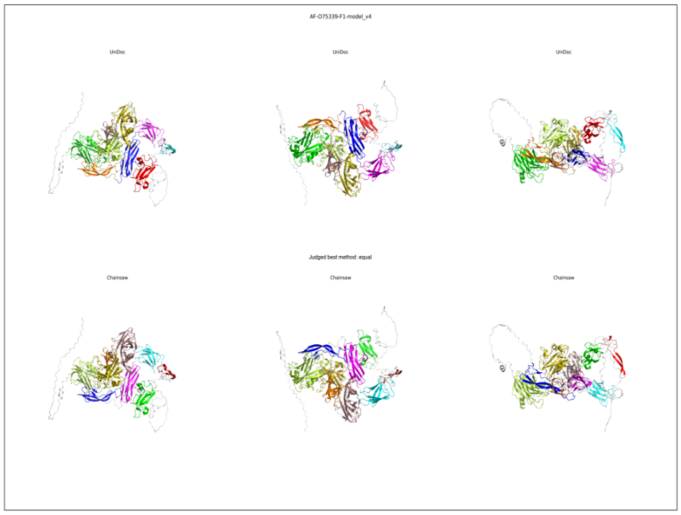

AF-O75339-F1-model_v4
UniDoc
UniDoc
UniDoc
Judged best method: equal
Chainsaw
Chainsaw
Chainsaw

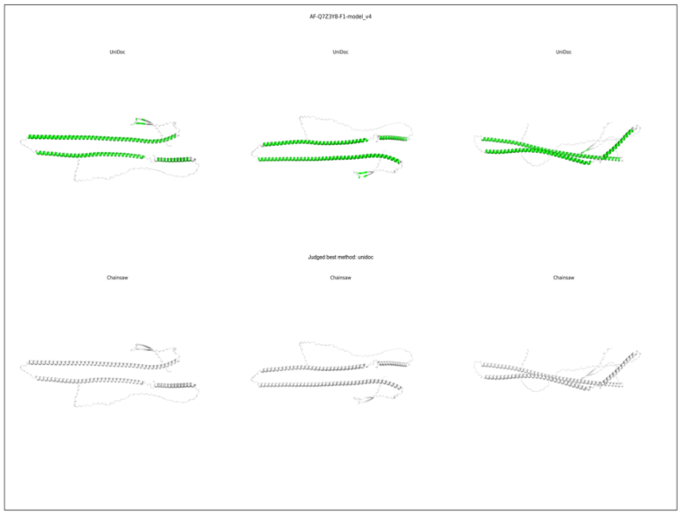

AF-Q7Z3Y8-F1-model_v4
UniDoc
UniDoc
UniDoc
Judged best method: unidoc
Chainsaw
Chainsaw
Chainsaw

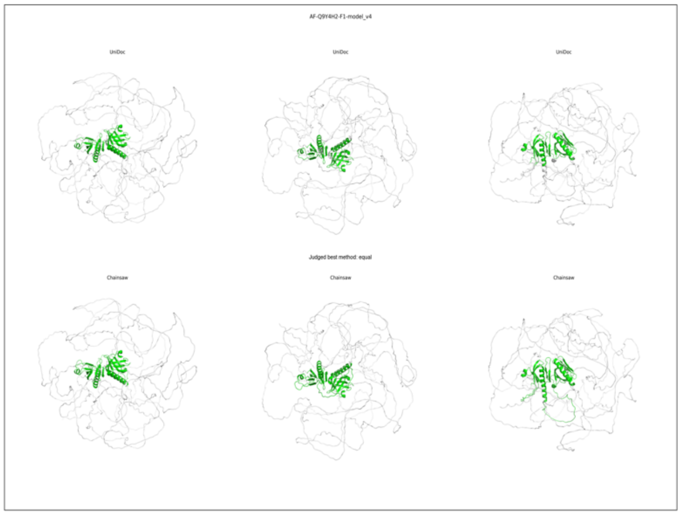
AF-Q9Y4H2-F1-model_v4
UniDoc
UniDoc
UniDoc
Judged best method: equal
Chainsaw
Chainsaw
Chainsaw

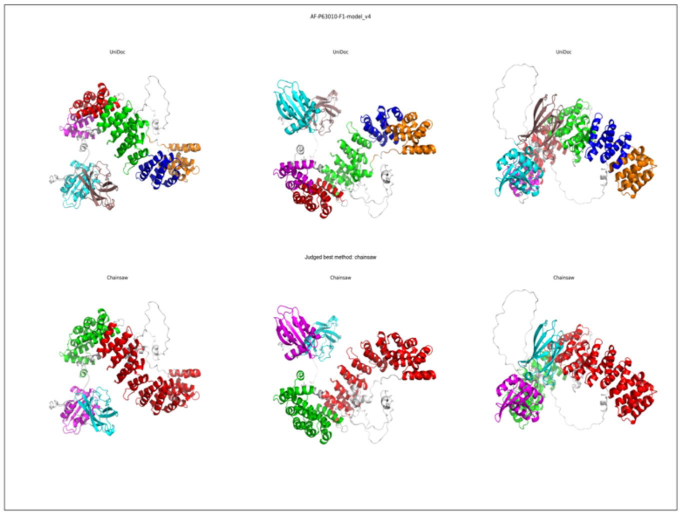
AF-P63010-F1-model_v4
UniDoc
UniDoc
UniDoc
Judged best method: chainsaw
Chainsaw
Chainsaw
Chainsaw

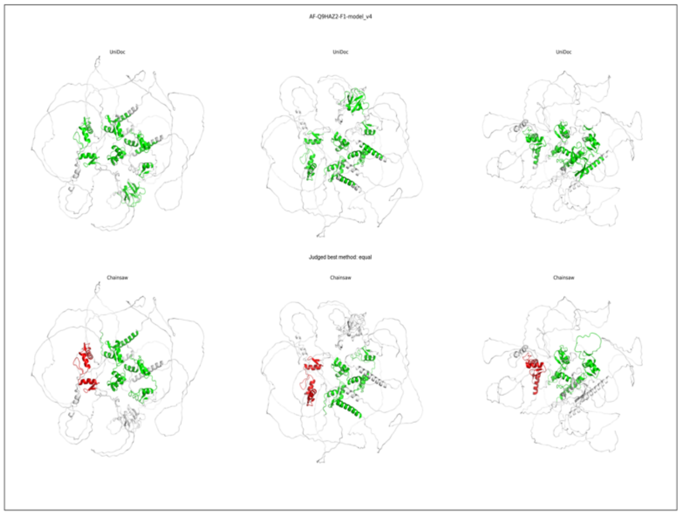
AF-Q9HAZ2-F1-model_v4
UniDoc
UniDoc
UniDoc
Judged best method: equal
Chainsaw
Chainsaw
Chainsaw

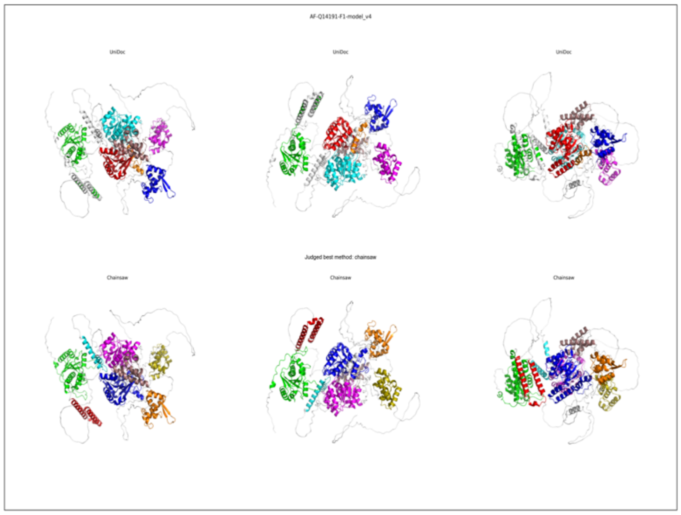
AF-Q14191-F1-model_v4
UniDoc
UniDoc
UniDoc
Judged best method: chainsaw
Chainsaw
Chainsaw
Chainsaw

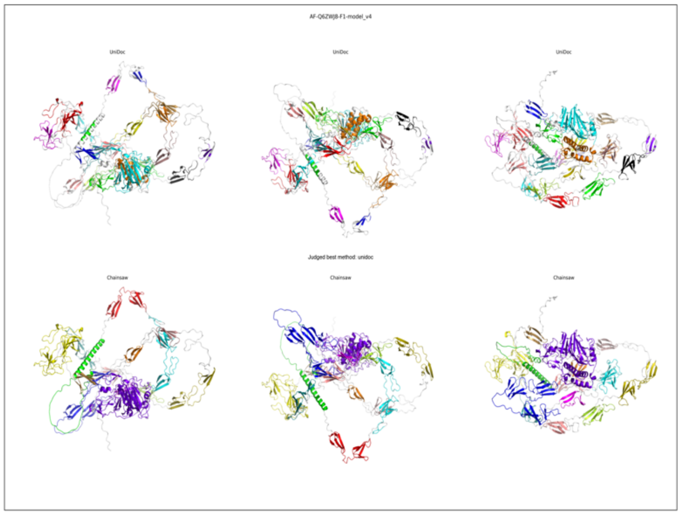
AF-Q6ZWJ8-F1-model_v4
UniDoc
UniDoc
UniDoc
Judged best method: unidoc
Chainsaw
Chainsaw
Chainsaw

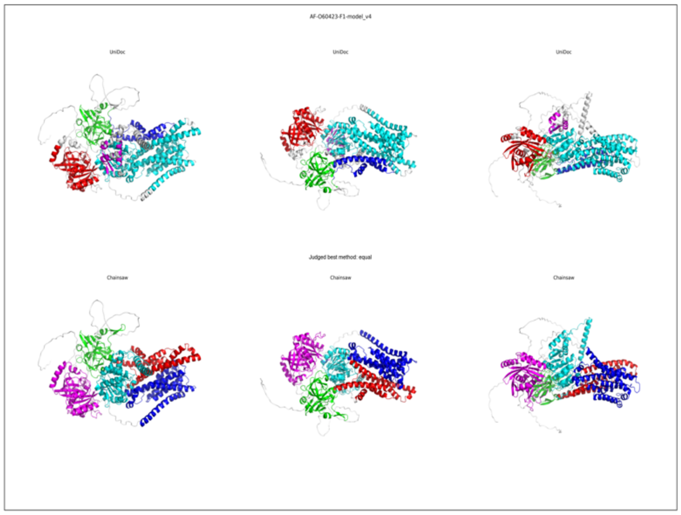
AF-O60423-F1-model_v4
UniDoc
UniDoc
UniDoc
Judged best method: equal
Chainsaw
Chainsaw
Chainsaw

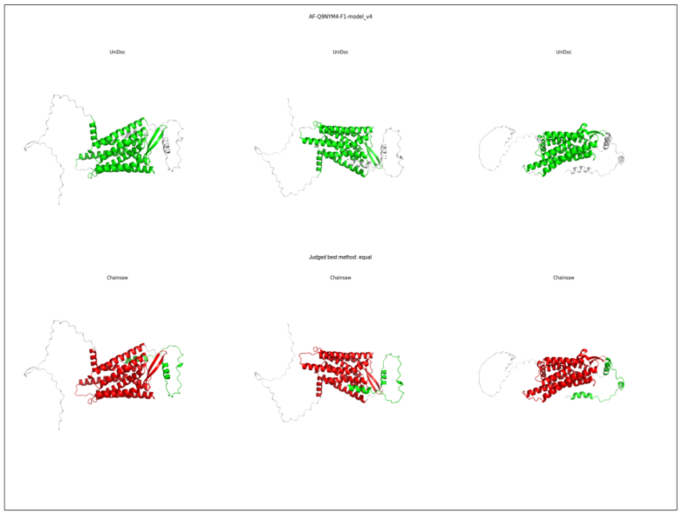
AF-Q9NYM4-F1-model_v4
UniDoc
UniDoc
UniDoc
Judged best method: equal
Chainsaw
Chainsaw
Chainsaw

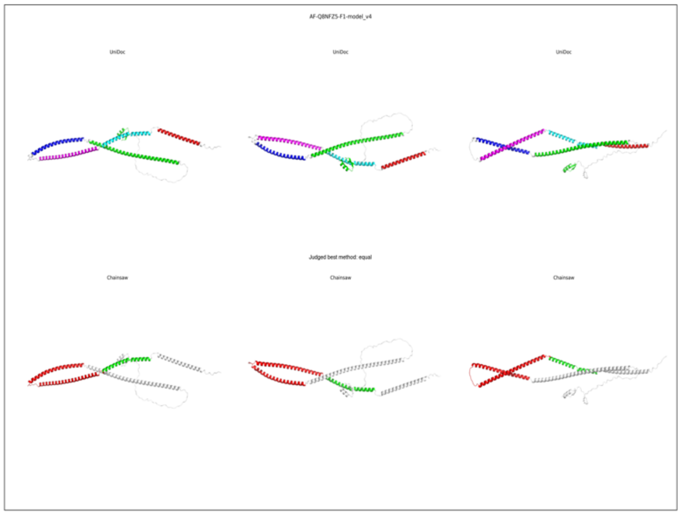
AF-Q8NFZ5-F1-model_v4
UniDoc
UniDoc
UniDoc
Judged best method: equal
Chainsaw
Chainsaw
Chainsaw

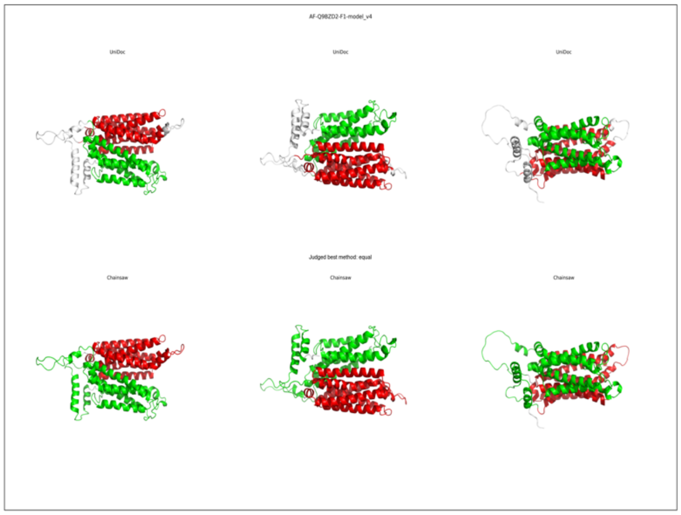
AF-Q9BZD2-F1-model_v4
UniDoc
UniDoc
UniDoc
Judged best method: equal
Chainsaw
Chainsaw
Chainsaw

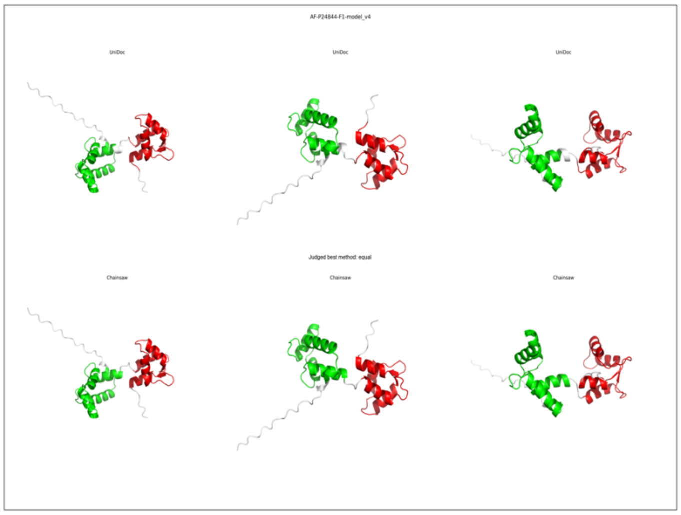
AF-P24844-F1-model_v4
UniDoc
UniDoc
UniDoc
Judged best method: equal
Chainsaw
Chainsaw
Chainsaw

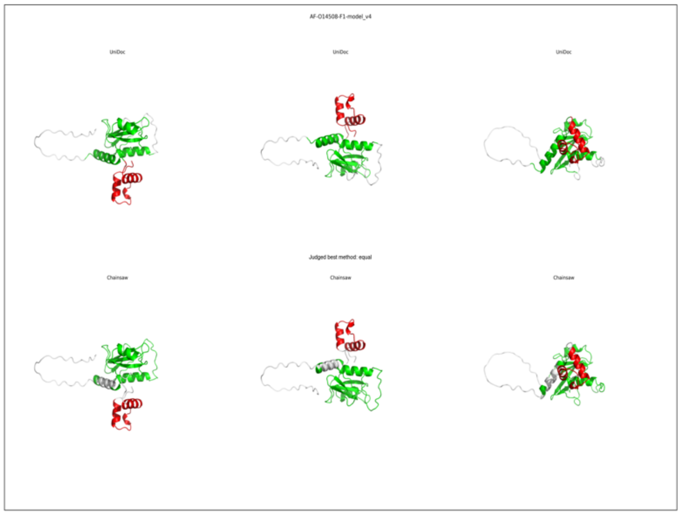
AF-O14508-F1-model_v4
UniDoc
UniDoc
UniDoc
Judged best method: equal
Chainsaw
Chainsaw
Chainsaw

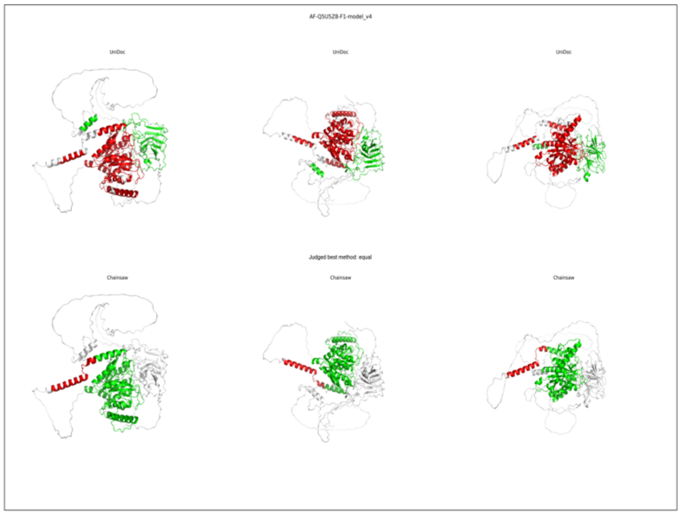
AF-Q5U5Z8-F1-model_v4
UniDoc
UniDoc
UniDoc
Judged best method: equal
Chainsaw
Chainsaw
Chainsaw

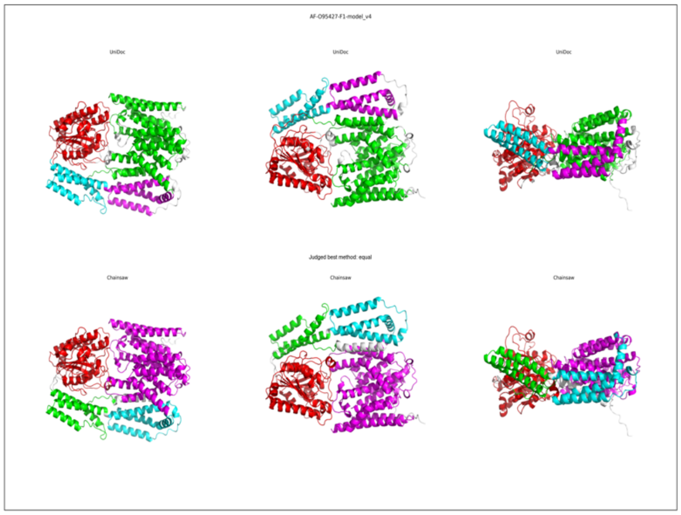
AF-O95427-F1-model_v4
UniDoc
UniDoc
UniDoc
Judged best method: equal
Chainsaw
Chainsaw
Chainsaw

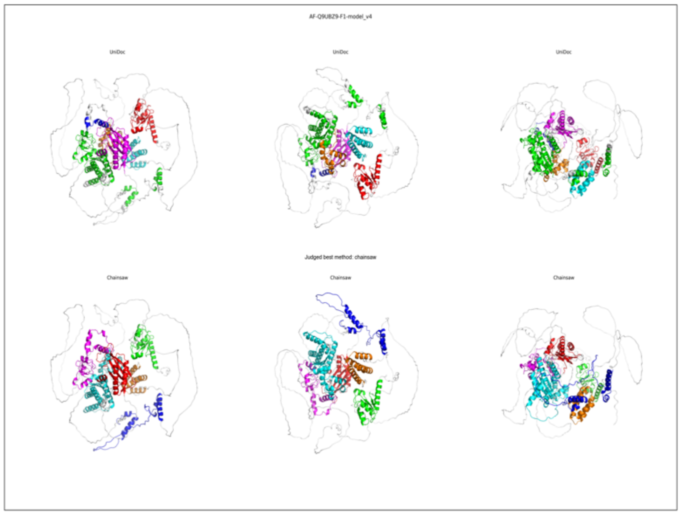
AF-Q9UBZ9-F1-model_v4
UniDoc
UniDoc
UniDoc
Judged best method: chainsaw
Chainsaw
Chainsaw
Chainsaw

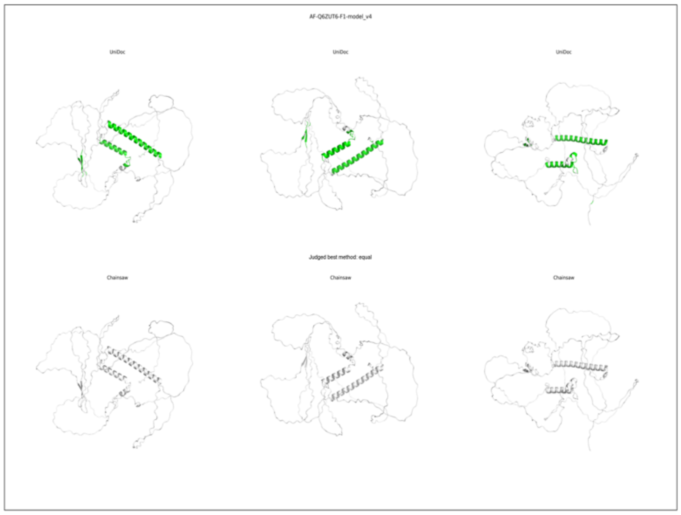
AF-Q6ZUT6-F1-model_v4
UniDoc
UniDoc
UniDoc
Judged best method: equal
Chainsaw
Chainsaw
Chainsaw

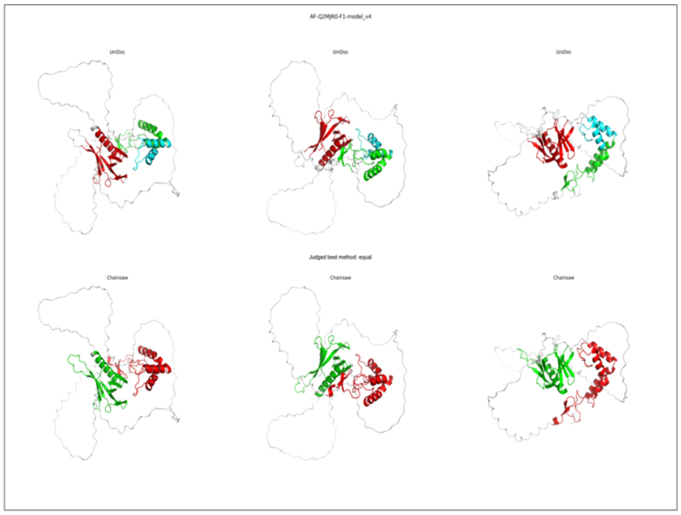
AF-Q2MJR0-F1-model_v4
UniDoc
UniDoc
UniDoc
Judged best method: equal
Chainsaw
Chainsaw
Chainsaw

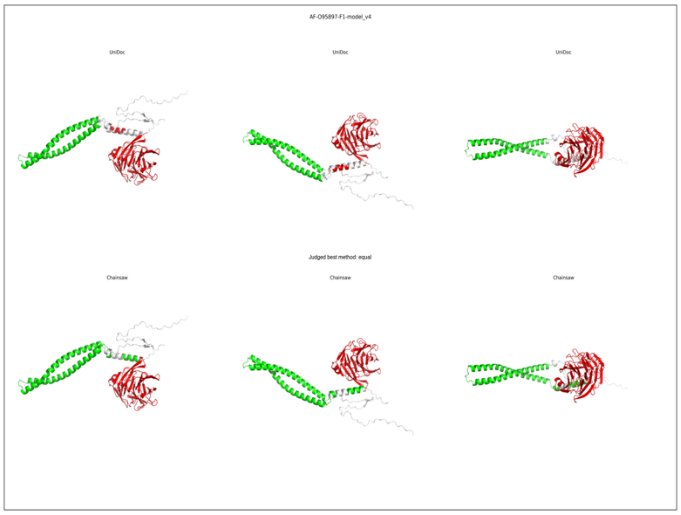
AF-O95897-F1-model_v4
UniDoc
UniDoc
UniDoc
Judged best method: equal
Chainsaw
Chainsaw
Chainsaw

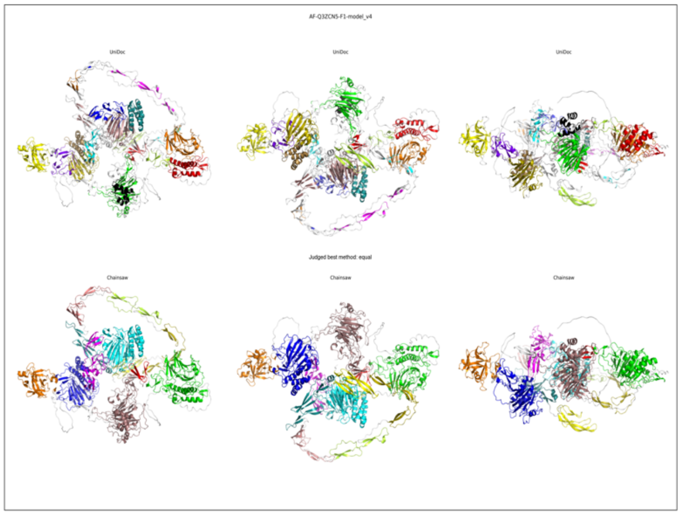
AF-Q3ZCN5-F1-model_v4
UniDoc
UniDoc
UniDoc
Judged best method: equal
Chainsaw
Chainsaw
Chainsaw

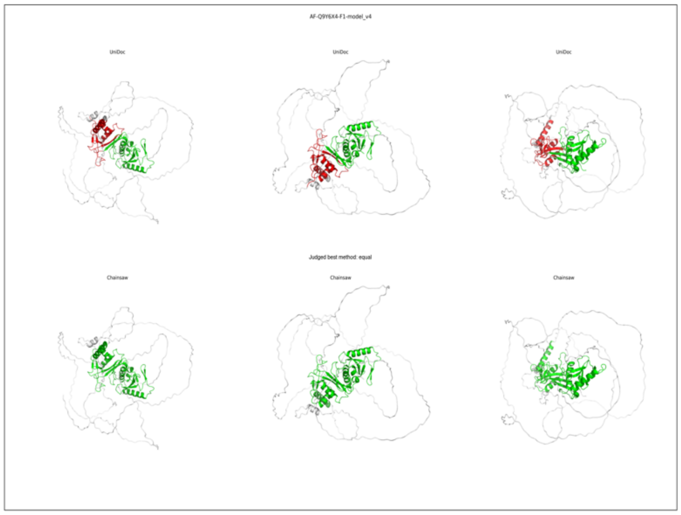
AF-Q9Y6X4-F1-model_v4
UniDoc
UniDoc
UniDoc
Judged best method: equal
Chainsaw
Chainsaw
Chainsaw

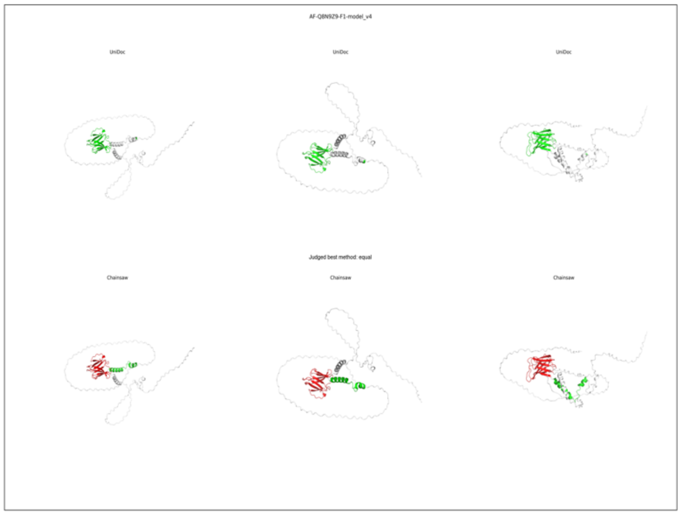
AF-Q8N9Z9-F1-model_v4
UniDoc
UniDoc
UniDoc
Judged best method: equal
Chainsaw
Chainsaw
Chainsaw

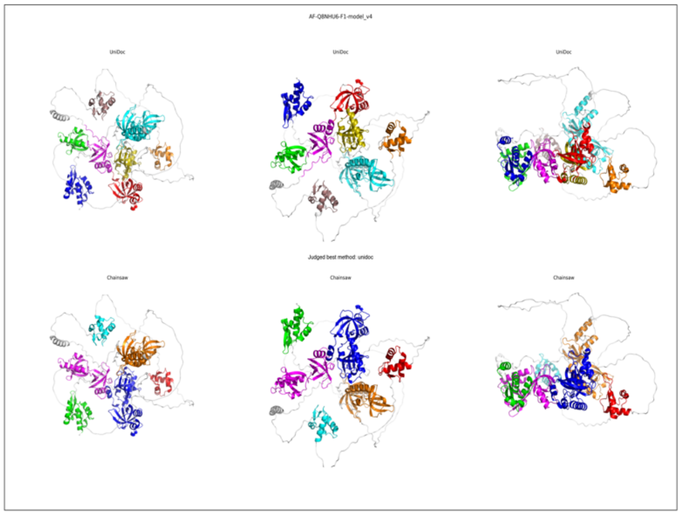
AF-Q8NHU6-F1-model_v4
UniDoc
UniDoc
UniDoc
Judged best method: unidoc
Chainsaw
Chainsaw
Chainsaw

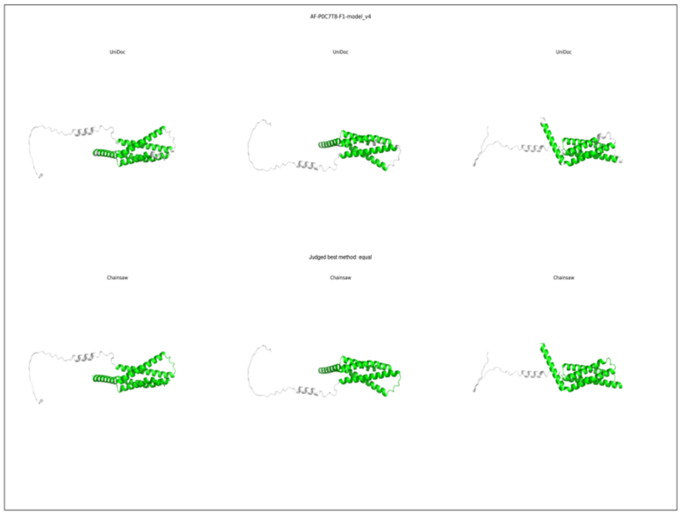
AF-P0C7T8-F1-model_v4
UniDoc
UniDoc
UniDoc
Judged best method: equal
Chainsaw
Chainsaw
Chainsaw

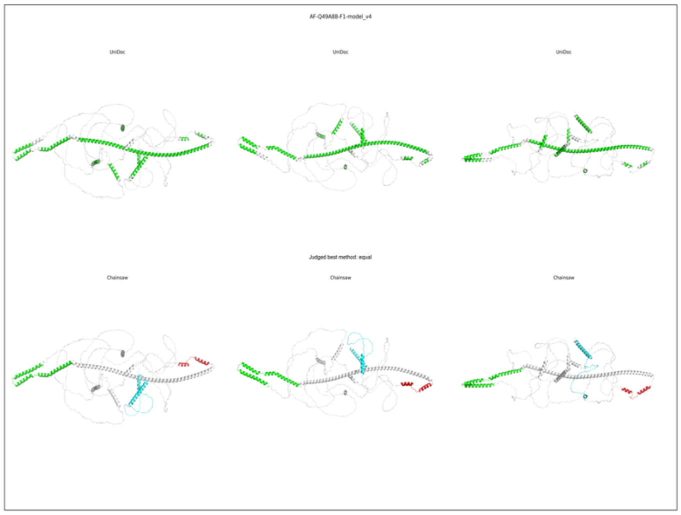
AF-Q49A88-F1-model_v4
UniDoc
UniDoc
UniDoc
Judged best method: equal
Chainsaw
Chainsaw
Chainsaw

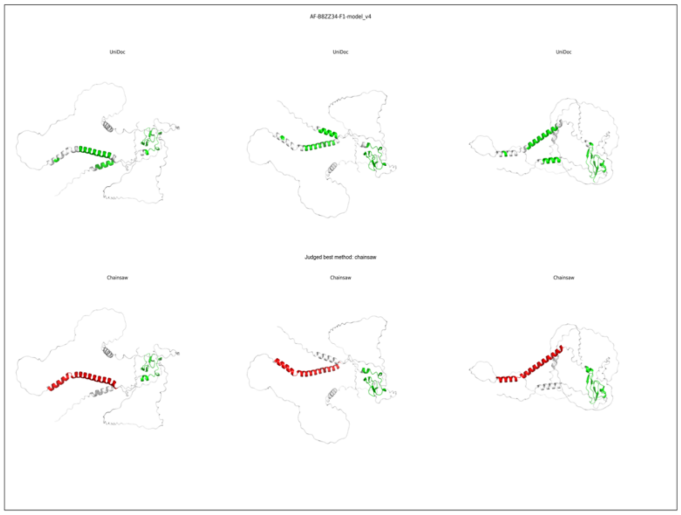
AF-B8ZZ34-F1-model_v4
UniDoc
UniDoc
UniDoc
Judged best method: chainsaw
Chainsaw
Chainsaw
Chainsaw

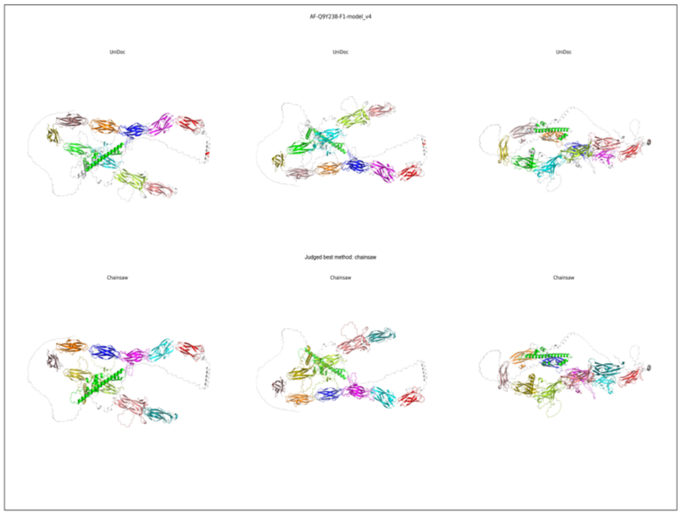
AF-Q9Y238-F1-model_v4
UniDoc
UniDoc
UniDoc
Judged best method: chainsaw
Chainsaw
Chainsaw
Chainsaw

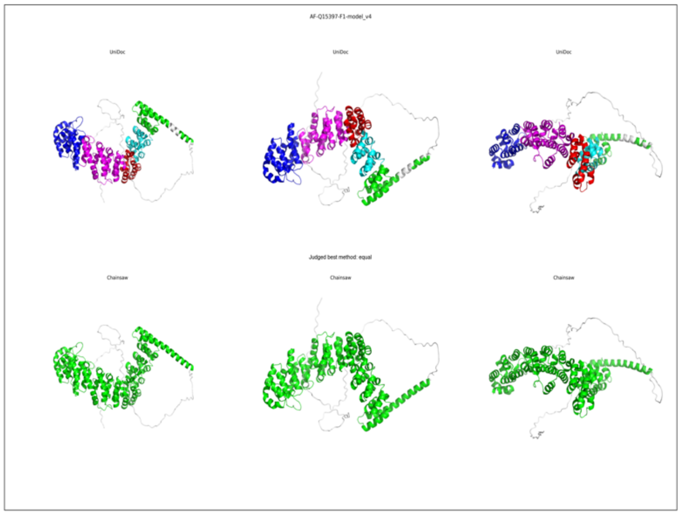
AF-Q15397-F1-model_v4
UniDoc
UniDoc
UniDoc
Judged best method: equal
Chainsaw
Chainsaw
Chainsaw

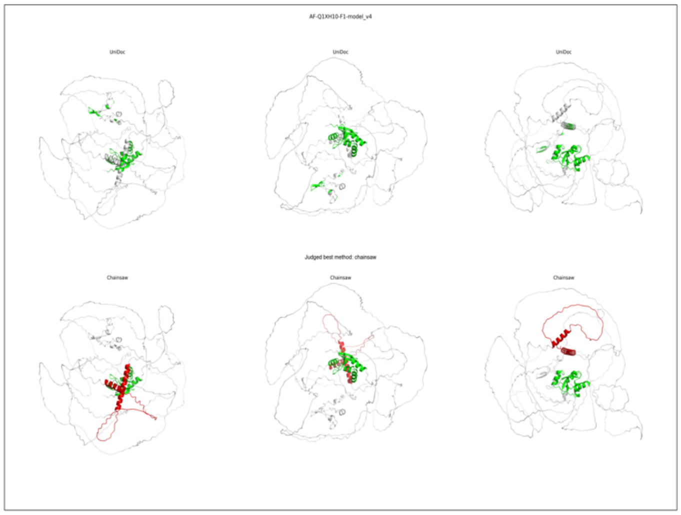
AF-Q1XH10-F1-model_v4
UniDoc
UniDoc
UniDoc
Judged best method: chainsaw
Chainsaw
Chainsaw
Chainsaw

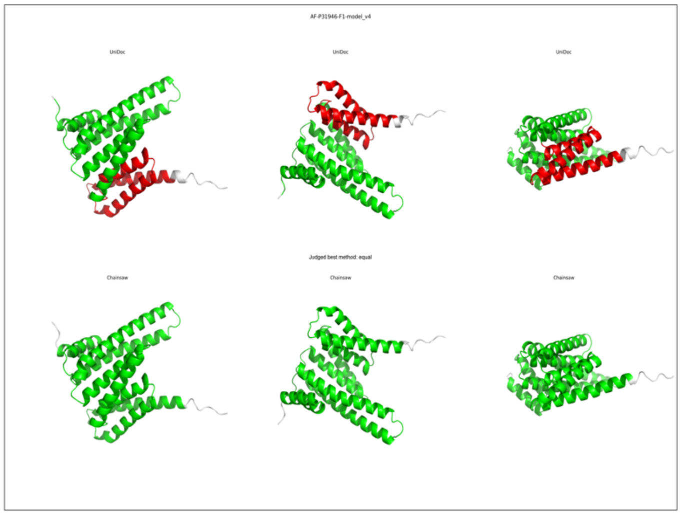
AF-P31946-F1-model_v4
UniDoc
UniDoc
UniDoc
Judged best method: equal
Chainsaw
Chainsaw
Chainsaw

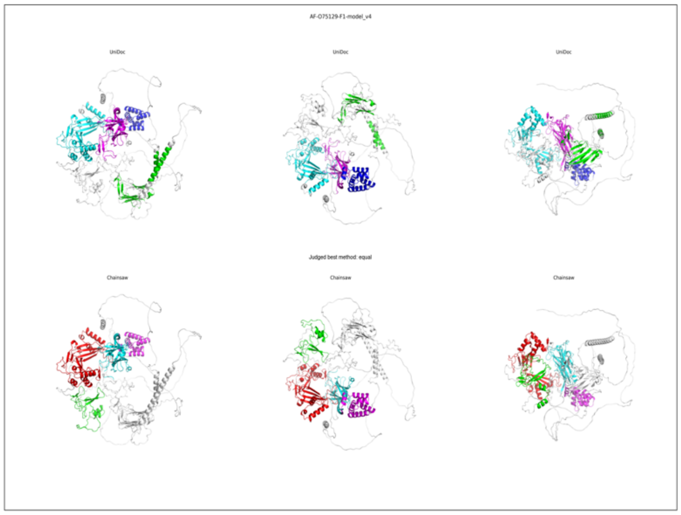
AF-O75129-F1-model_v4
UniDoc
UniDoc
UniDoc
Judged best method: equal
Chainsaw
Chainsaw
Chainsaw

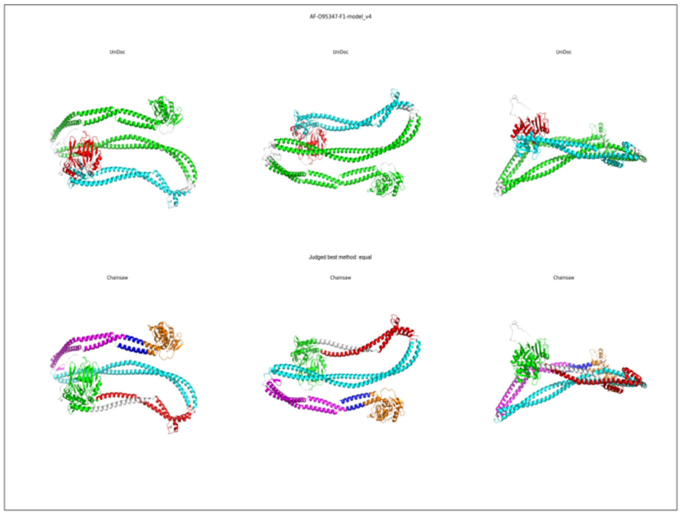
AF-O95347-F1-model_v4
UniDoc
UniDoc
UniDoc
Judged best method: equal
Chainsaw
Chainsaw
Chainsaw

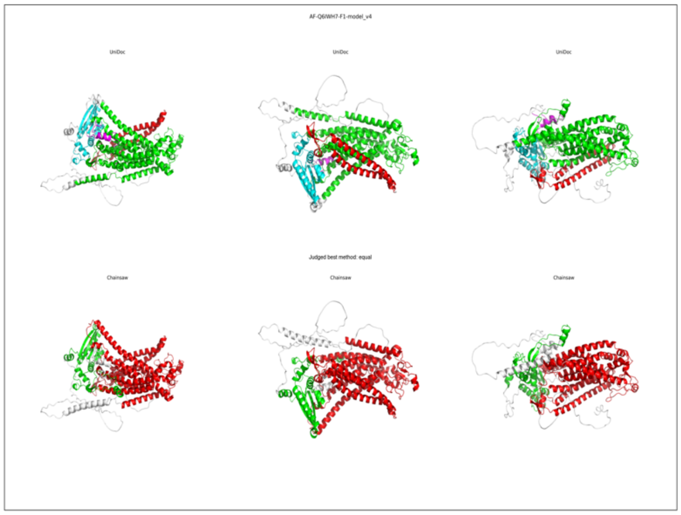
AF-Q6IWH7-F1-model_v4
UniDoc
UniDoc
UniDoc
Judged best method: equal
Chainsaw
Chainsaw
Chainsaw

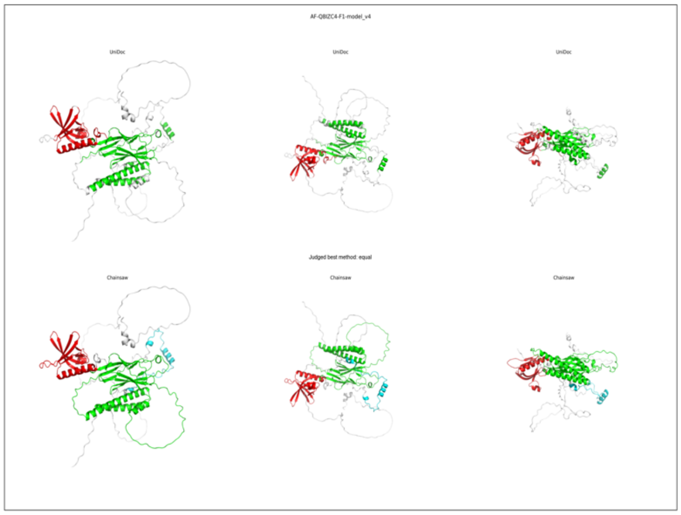
AF-Q8IZC4-F1-model_v4
UniDoc
UniDoc
UniDoc
Judged best method: equal
Chainsaw
Chainsaw
Chainsaw

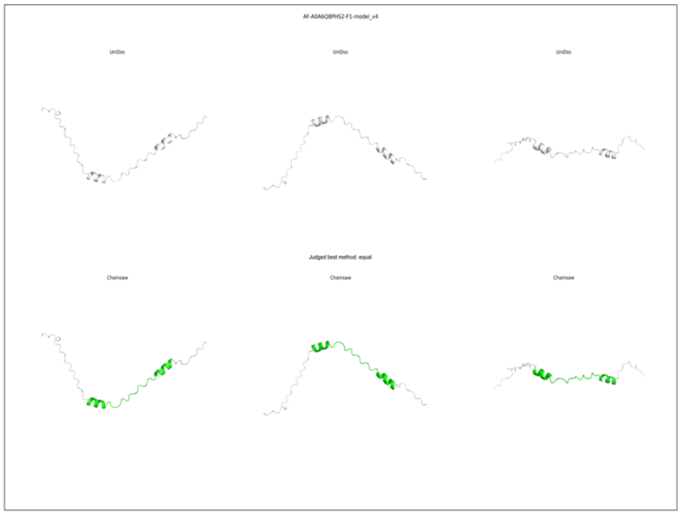
AF-A0A6Q8PHS2-F1-model_v4
UniDoc
UniDoc
UniDoc
Judged best method: equal
Chainsaw
Chainsaw
Chainsaw

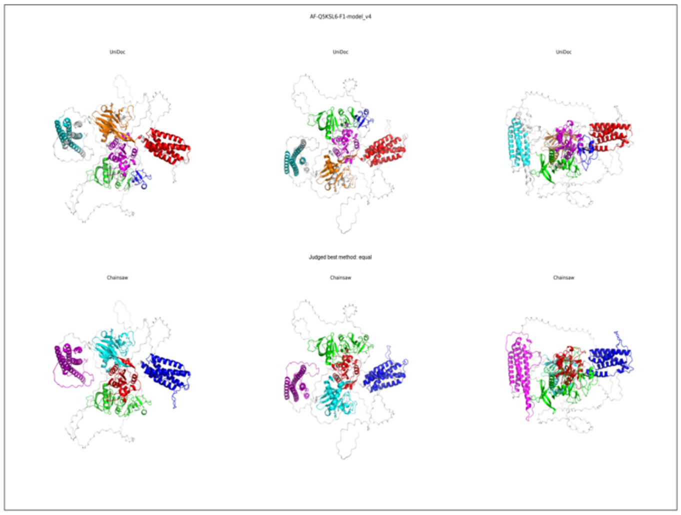
AF-Q5KSL6-F1-model_v4
UniDoc
UniDoc
UniDoc
Judged best method: equal
Chainsaw
Chainsaw
Chainsaw

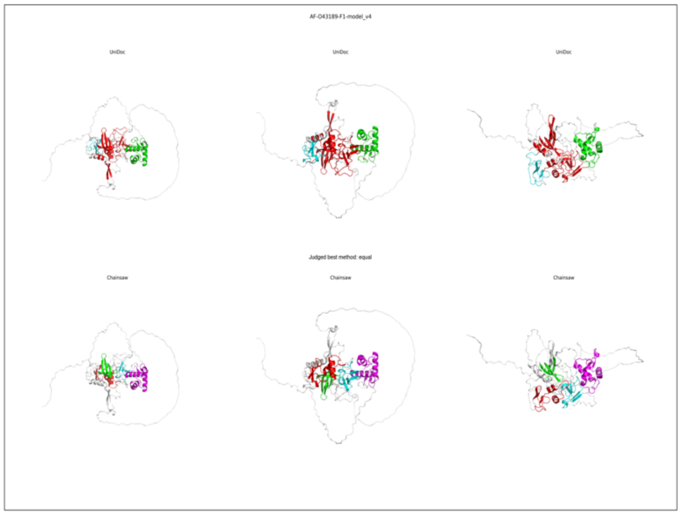
AF-O43189-F1-model_v4
UniDoc
UniDoc
UniDoc
Judged best method: equal
Chainsaw
Chainsaw
Chainsaw

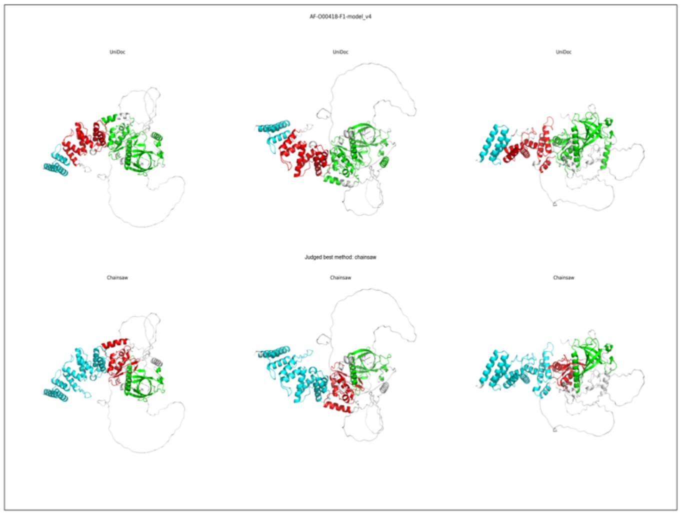
AF-O00418-F1-model_v4
UniDoc
UniDoc
UniDoc
Judged best method: chainsaw
Chainsaw
Chainsaw
Chainsaw

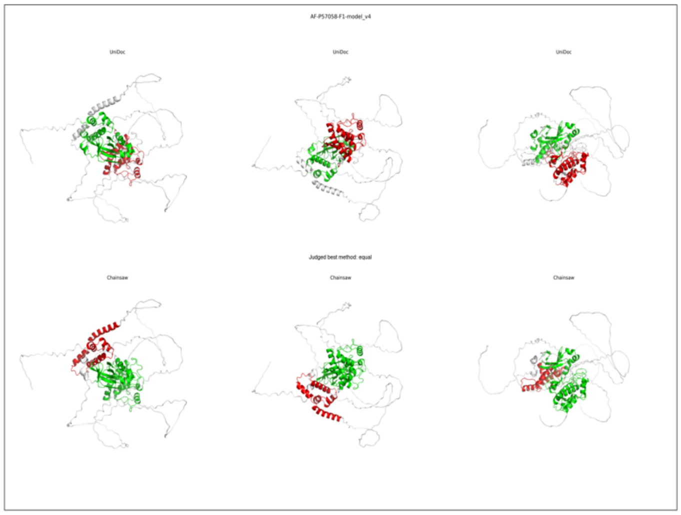
AF-P57058-F1-model_v4
UniDoc
UniDoc
UniDoc
Judged best method: equal
Chainsaw
Chainsaw
Chainsaw

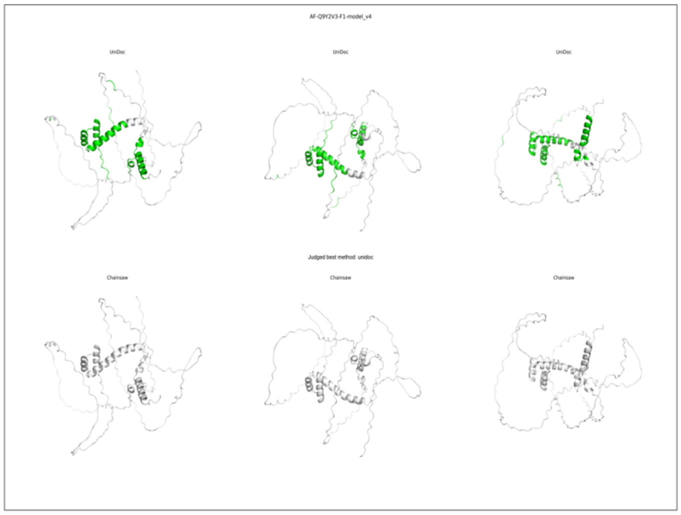
AF-Q9Y2V3-F1-model_v4
UniDoc
UniDoc
UniDoc
Judged best method: unidoc
Chainsaw
Chainsaw
Chainsaw

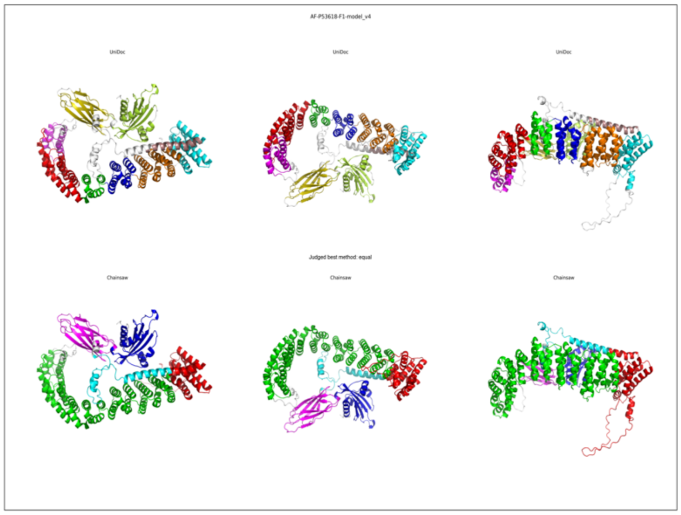
AF-P53618-F1-model_v4
UniDoc
UniDoc
UniDoc
Judged best method: equal
Chainsaw
Chainsaw
Chainsaw

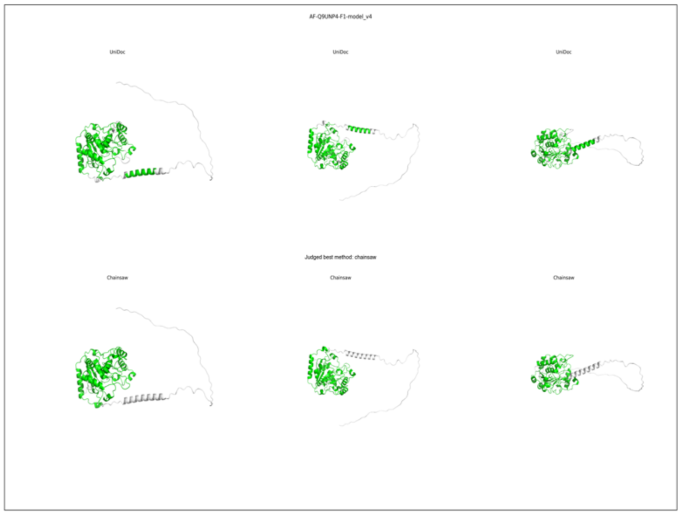
AF-Q9UNP4-F1-model_v4
UniDoc
UniDoc
UniDoc
Judged best method: chainsaw
Chainsaw
Chainsaw
Chainsaw

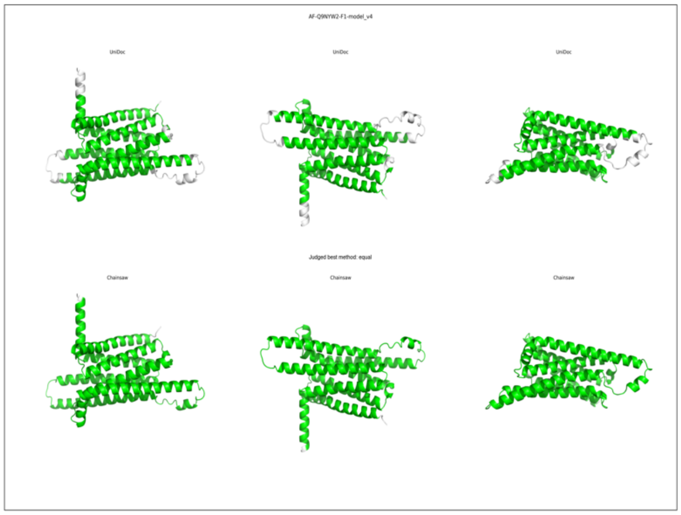
AF-Q9NYW2-F1-model_v4
UniDoc
UniDoc
UniDoc
Judged best method: equal
Chainsaw
Chainsaw
Chainsaw

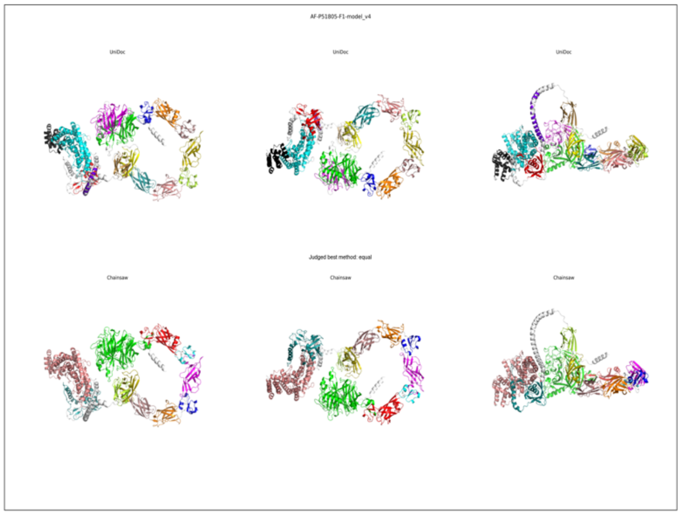
AF-P51805-F1-model_v4
UniDoc
UniDoc
UniDoc
Judged best method: equal
Chainsaw
Chainsaw
Chainsaw

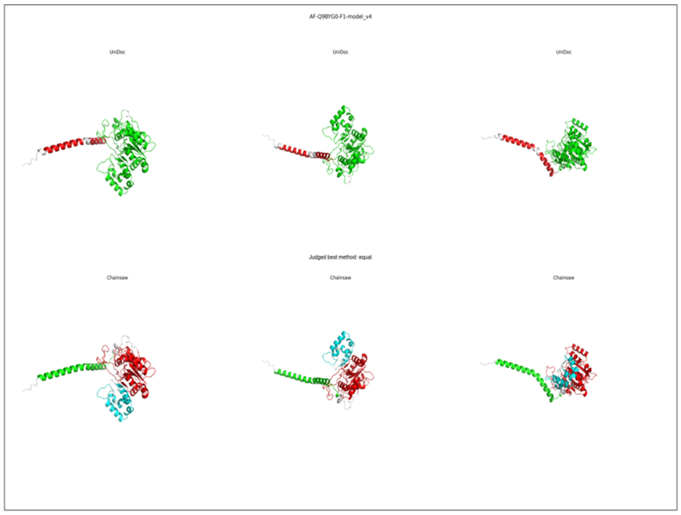
AF-Q9BYG0-F1-model_v4
UniDoc
UniDoc
UniDoc
Judged best method: equal
Chainsaw
Chainsaw
Chainsaw

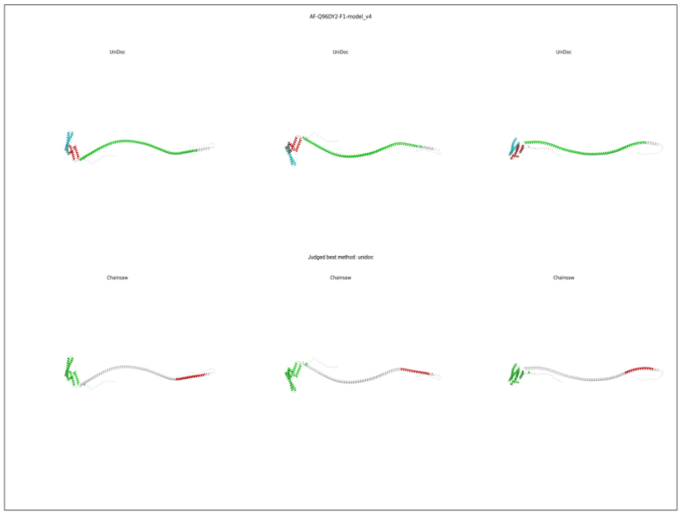
AF-Q96DY2-F1-model_v4
UniDoc
UniDoc
UniDoc
Judged best method: unidoc
Chainsaw
Chainsaw
Chainsaw

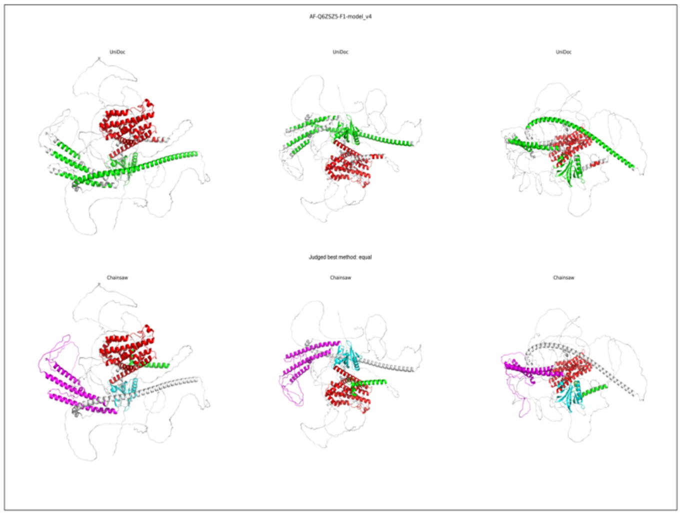
AF-Q6ZSZ5-F1-model_v4
UniDoc
UniDoc
UniDoc
Judged best method: equal
Chainsaw
Chainsaw
Chainsaw

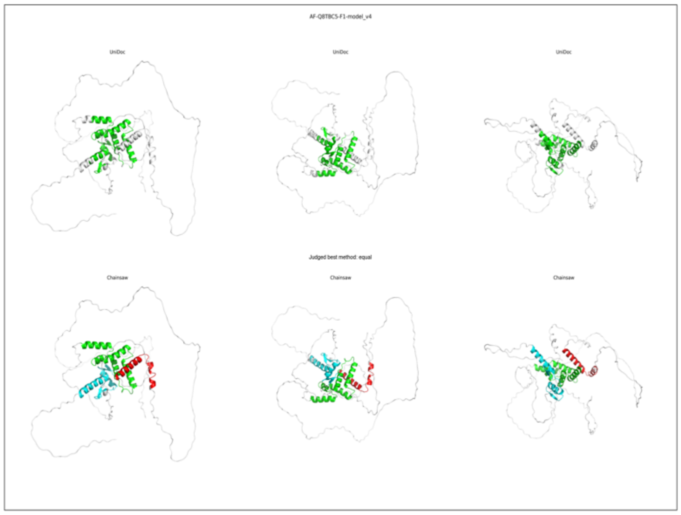
AF-Q8TBC5-F1-model_v4
UniDoc
UniDoc
UniDoc
Judged best method: equal
Chainsaw
Chainsaw
Chainsaw

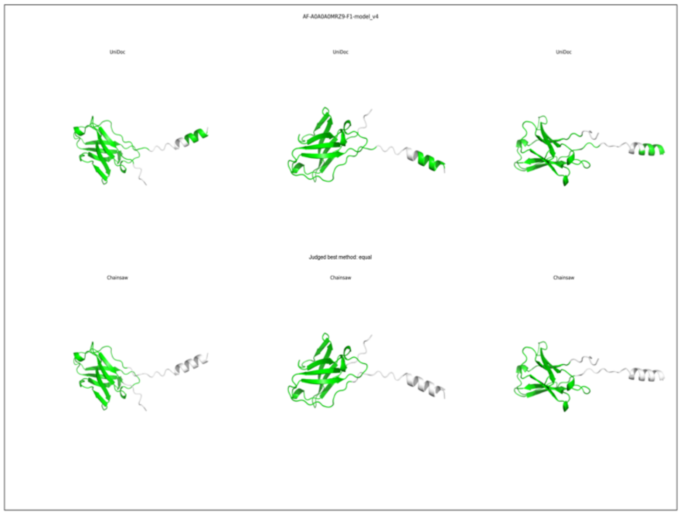
AF-A0A0A0MRZ9-F1-model_v4
UniDoc
UniDoc
UniDoc
Judged best method: equal
Chainsaw
Chainsaw
Chainsaw

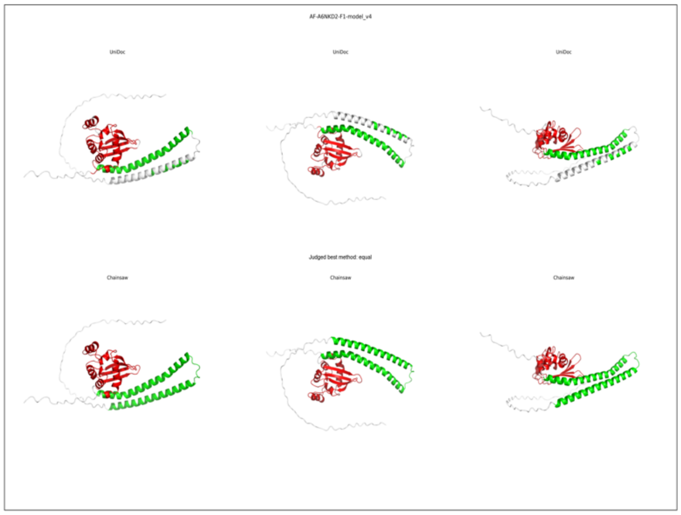
AF-A6NKD2-F1-model_v4
UniDoc
UniDoc
UniDoc
Judged best method: equal
Chainsaw
Chainsaw
Chainsaw

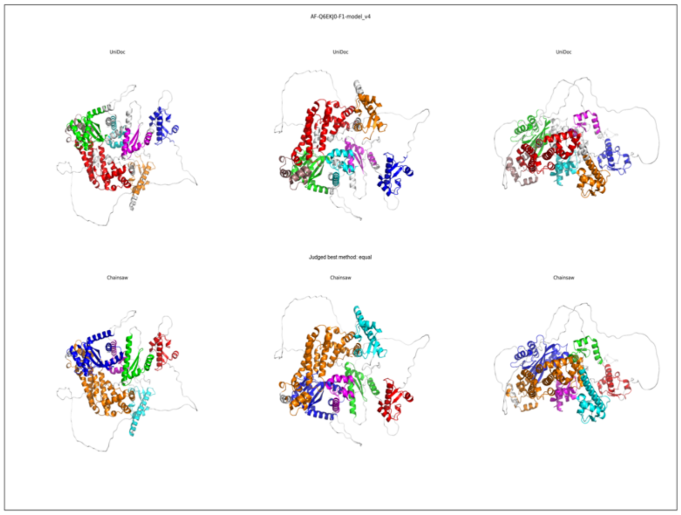

AF-Q6EKJ0-F1-model_v4
UniDoc
UniDoc
UniDoc
Judged best method: equal
Chainsaw
Chainsaw
Chainsaw

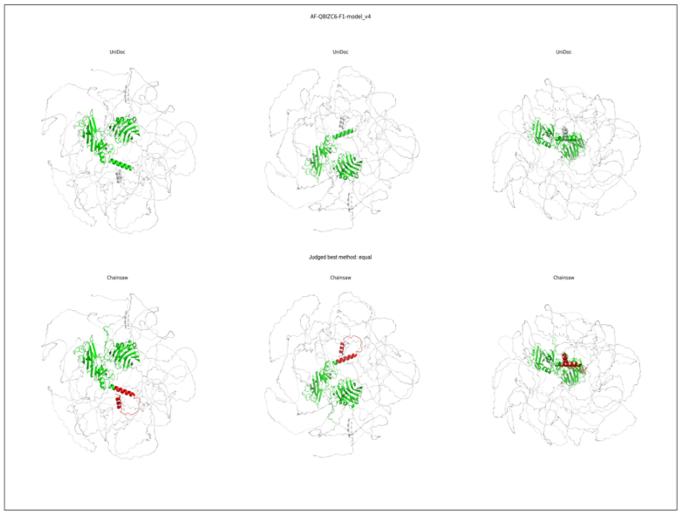

AF-Q8IZC6-F1-model_v4
UniDoc
UniDoc
UniDoc
Judged best method: equal
Chainsaw
Chainsaw
Chainsaw

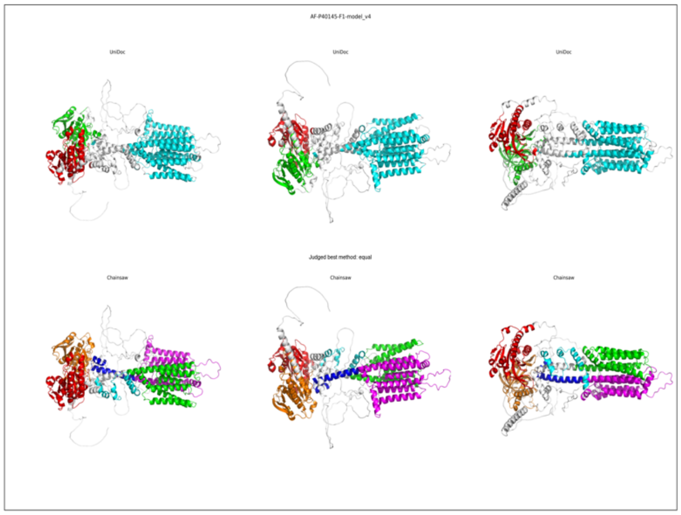

AF-P40145-F1-model_v4
UniDoc
UniDoc
UniDoc
Judged best method: equal
Chainsaw
Chainsaw
Chainsaw

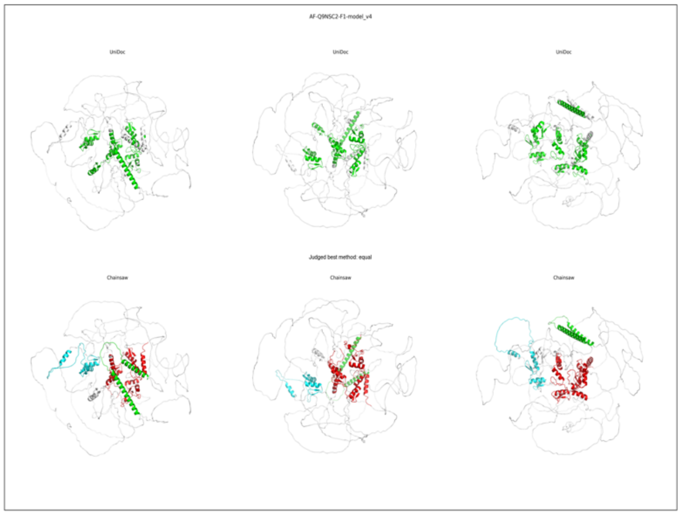

AF-Q9NSC2-F1-model_v4
UniDoc
UniDoc
UniDoc
Judged best method: equal
Chainsaw
Chainsaw
Chainsaw

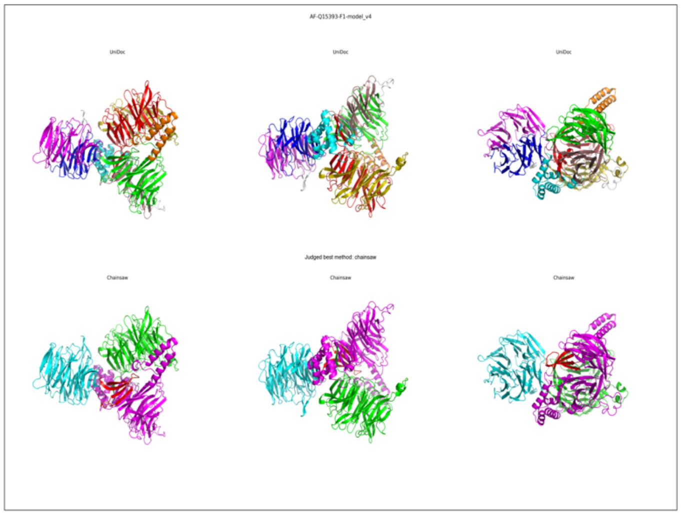

AF-Q15393-F1-model_v4
UniDoc
UniDoc
UniDoc
Judged best method: chainsaw
Chainsaw
Chainsaw
Chainsaw

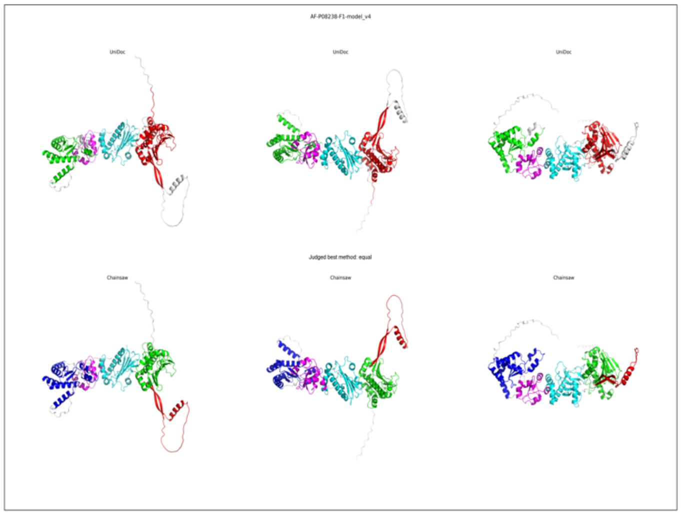

AF-P08238-F1-model_v4
UniDoc
UniDoc
UniDoc
Judged best method: equal
Chainsaw
Chainsaw
Chainsaw

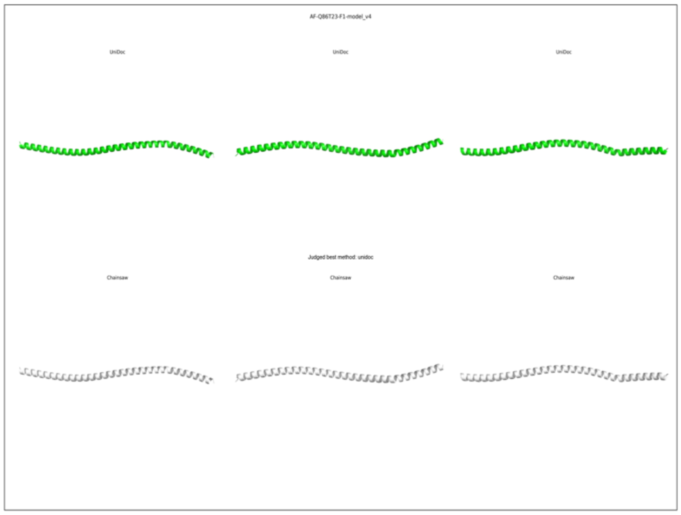
AF-Q86T23-F1-model_v4
UniDoc
UniDoc
UniDoc
Judged best method: unidoc
Chainsaw
Chainsaw
Chainsaw

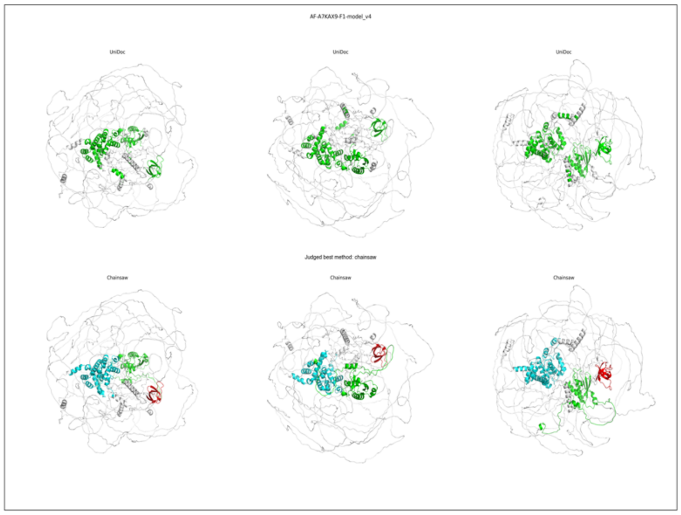
AF-A7KAX9-F1-model_v4
UniDoc
UniDoc
UniDoc
Judged best method: chainsaw
Chainsaw
Chainsaw
Chainsaw

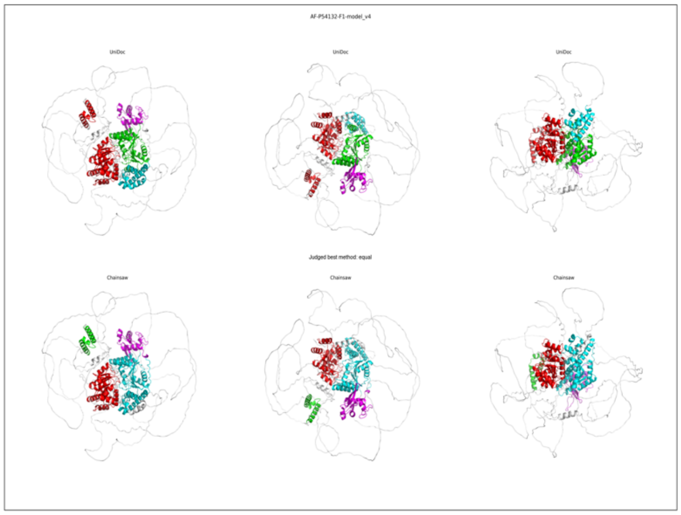
AF-P54132-F1-model_v4
UniDoc
UniDoc
UniDoc
Judged best method: equal
Chainsaw
Chainsaw
Chainsaw

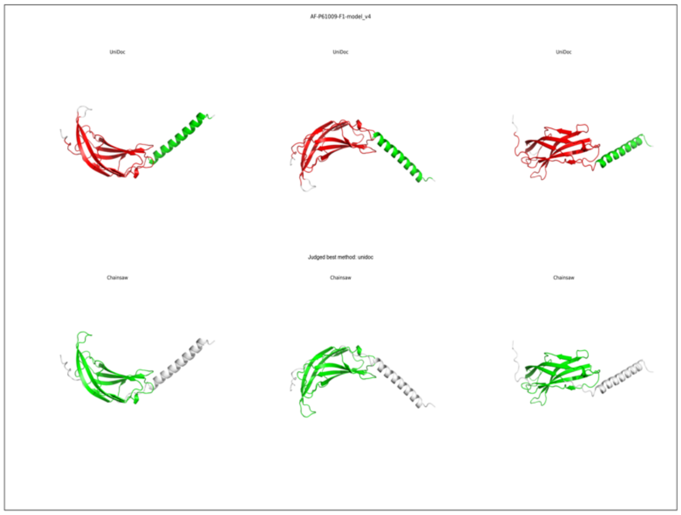
AF-P61009-F1-model_v4
UniDoc
UniDoc
UniDoc
Judged best method: unidoc
Chainsaw
Chainsaw
Chainsaw

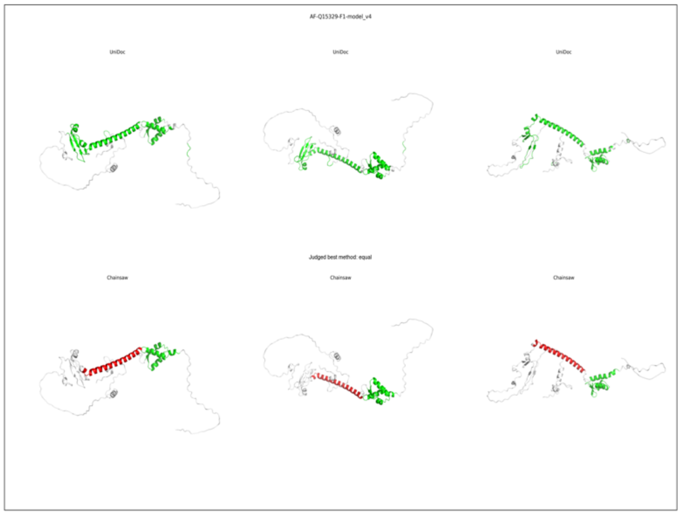
AF-Q15329-F1-model_v4
UniDoc
UniDoc
UniDoc
Judged best method: equal
Chainsaw
Chainsaw
Chainsaw

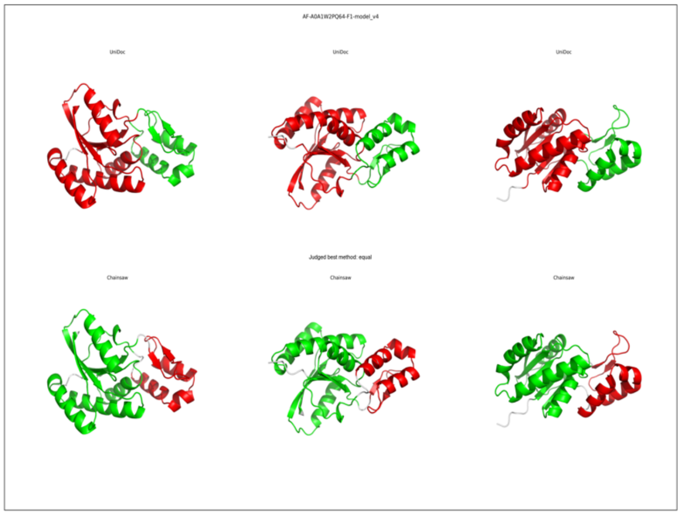
AF-A0A1W2PQ64-F1-model_v4
UniDoc
UniDoc
UniDoc
Judged best method: equal
Chainsaw
Chainsaw
Chainsaw

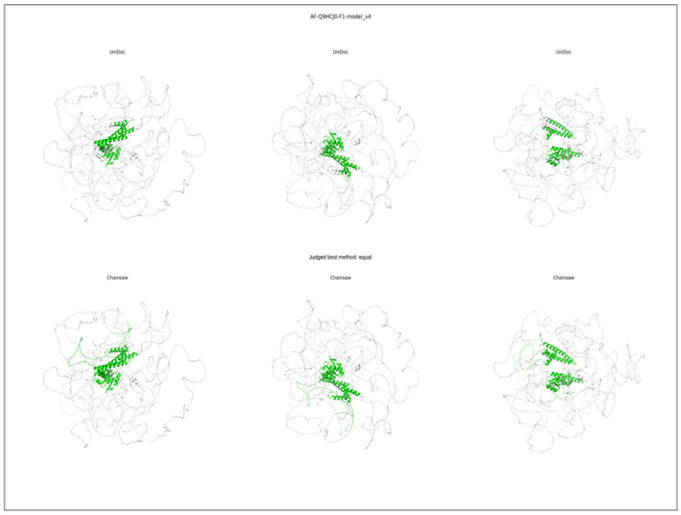
AF-Q9HCJ0-F1-model_v4
UniDoc
UniDoc
UniDoc
Judged best method: equal
Chainsaw
Chainsaw
Chainsaw

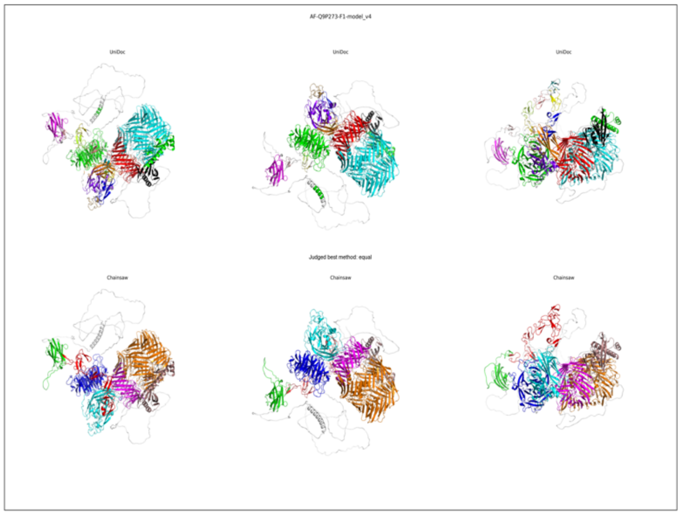
AF-Q9P273-F1-model_v4
UniDoc
UniDoc
UniDoc
Judged best method: equal
Chainsaw
Chainsaw
Chainsaw
